# Syntheses, crystal structures, and comparisons of rare-earth oxyapatites Ca_2_ *RE*_8_(SiO_4_)_6_O_2_ (*RE* = La, Nd, Sm, Eu, or Yb) and NaLa_9_(SiO_4_)_6_O_2_

**DOI:** 10.1107/S2056989019008442

**Published:** 2019-06-21

**Authors:** Jarrod V. Crum, Saehwa Chong, Jacob A. Peterson, Brian J. Riley

**Affiliations:** a Pacific Northwest National Laboratory, Richland, WA 99352, USA

**Keywords:** oxyapatite, lanthanide oxyapatite, rare-earth oxyapatite, powder diffraction

## Abstract

Six different rare-earth oxyapatites, including Ca_2_ *RE*_8_(SiO_4_)_6_O_2_ (*RE* = La, Nd, Sm, Eu, or Yb) and NaLa_9_(SiO_4_)_6_O_2_, were synthesized using solution-based processes followed by cold pressing and sinter­ing. The crystal structures of the synthesized oxyapatites were determined from powder X-ray diffraction (P-XRD) and their chemistries verified with electron probe microanalysis (EPMA). All the oxyapatites were isostructural within the hexa­gonal space group *P*6_3_/*m* and showed similar unit-cell parameters. The isolated [SiO_4_]^4−^ tetra­hedra in each crystal are linked by the cations at the 4*f* and 6*h* sites occupied by *RE* ^3+^ and Ca^2+^ in Ca_2_ *RE*_8_(SiO_4_)_6_O_2_ or La^3+^ and Na^+^ in NaLa_9_(SiO_4_)_6_O_2_. The lattice parameters, cell volumes, and densities of the synthesized oxyapatites fit well to the trendlines calculated from literature values.

## Abstract 2

X-ray powder diffraction was used to determine the structures of six different rare-earth oxyapatites that were shown to be isostructural.

## Chemical context   

For immobilization of the radionuclides in the high-level waste (HLW) raffinate stream following aqueous reprocessing of used nuclear fuel, glass-ceramic waste forms are being developed as an alternative to borosilicate glass (Crum *et al.*, 2012 ▸, 2014 ▸, 2016 ▸). As a result of the chemical diversity in the HLW raffinate stream, several crystalline phases including powellite [(*AE*)MoO_4_], rare-earth borosilicate [(*RE*)_3_BSi_2_O_10_], cerian­ite (Zr_*x*_Ce_1-*x*_O_2_), and oxyapatite [(*AE*)_2_(*RE*)_8_(SiO_4_)_6_O_2_], where *AE* and *RE* are alkaline earth and rare-earth metals, respectively, crystallize from the glass matrix upon slow cooling inside the waste canister during the waste form fabrication process (Crum *et al.*, 2012 ▸, 2014 ▸, 2016 ▸). Understanding the crystal chemistry and formation of these phases is important in the development of the glass-ceramic waste forms. In the actual waste form, each crystalline phase containing *RE* elements contains a distribution matching that within the waste stream. However, for characterization purposes, simplified versions of these phases were synthesized so that the individual contributions towards the chemical durability of the overall waste form could be evaluated (Neeway *et al.*, 2019 ▸). In this work, we report the synthesis method and crystal structures of oxyapatites, Ca_2_ *RE*_8_(SiO_4_)_6_O_2_ (*RE* = La, Nd, Sm, Eu, Yb) and NaLa_9_(SiO_4_)_6_O_2_. Additional information on the synthesis can be found in our previous paper (Peterson *et al.*, 2018 ▸). The crystal structures of Ca_2_ *RE*_8_(SiO_4_)_6_O_2_ (*RE* = La, Ce, Nd; Schroeder & Mathew, 1978 ▸; Fahey *et al.*, 1985 ▸; Massoni *et al.*, 2018 ▸) were studied in detail previously as in Inorganic Crystal Structure Database (ICSD) entries 5268, 92041, and 62174 for La, Ce, and Nd, respectively, but the crystal structures of Ca_2_ *RE*_8_(SiO_4_)_6_O_2_ (*RE* = Sm, Eu, Yb) and NaLa_9_(SiO_4_)_6_O_2_ have never been reported in detail before. The oxyapatites with La and Nd in this study are re-refined structures and are reported to compare with previously reported structures. We compare the general structural parameters of these isostructural oxyapatites with different *RE* cations.

## Structural commentary   

The general formula of silicate oxyapatites containing rare-earth metals along with alkalis or alkaline earths (without OH^−^) is *A*_1+_ *_x_RE*_9–*x*_(SiO_4_)_6_O_2_ [0 ≤ *x* ≤ 1; *A* = Li (Setoguchi, 1990 ▸; Ito, 1968 ▸), Na (Setoguchi, 1990 ▸; Ito, 1968 ▸), Ca (Fahey *et al.*, 1985 ▸; Lambert *et al.*, 2006 ▸; Ito, 1968 ▸), Ba (Lambert *et al.*, 2006 ▸; Ito, 1968 ▸), Sr (Lambert *et al.*, 2006 ▸; Ito, 1968 ▸; Latshaw *et al.*, 2016 ▸), Mg (Ito, 1968 ▸), Mn (Ito, 1968 ▸), Pb (Ito, 1968 ▸); *Ln* = La (Lambert *et al.*, 2006 ▸; Ito, 1968 ▸), Ce (Massoni *et al.*, 2018 ▸), Pr (Leu *et al.*, 2011 ▸; Sakakura *et al.*, 2010 ▸), Nd (Fahey *et al.*, 1985 ▸; Setoguchi, 1990 ▸; Ito, 1968 ▸; Latshaw *et al.*, 2016 ▸), Sm (Ito, 1968 ▸), Eu (Setoguchi, 1990 ▸), Gd (Ito, 1968 ▸), Tb (Leu *et al.*, 2011 ▸), Dy (Ito, 1968 ▸), Er (Ito, 1968 ▸), Tm (Leu *et al.*, 2011 ▸), Lu (Ito, 1968 ▸), Yb (Latshaw *et al.*, 2016 ▸), and Y (Ito, 1968 ▸)]. In this work, oxyaptites with different *A* and *RE* are abbreviated as *A*-*RE* [*e.g.* Ca-La denotes Ca_2_La_8_(SiO_4_)_6_O_2_]. These apatites generally crystallize in space group *P*6_3_/*m*; however, *P*6_3_ (Lambert *et al.*, 2006 ▸) and *P* 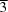 (Sansom *et al.*, 2004 ▸) space-group symmetries have been reported as well.

In a study by Lambert *et al.* (2006 ▸), the structural models of La_9.33_(SiO_4_)_6_O_2_, La_9_Ba(SiO_4_)6O_2+δ_, La_9_Sr(SiO_4_)_6_O_2+δ_, and La_9_Ca(SiO_4_)_6_O_2+δ_ were refined against neutron powder diffraction data within the *P*6_3_/*m*, *P*6_3_, and *P* 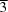 space groups, and the best results were obtained using the *P*6_3_ symmetry. They found that a symmetry difference by *m* ^[001]^ between *P*6_3_/*m* and *P*6_3_ allows two independent sites for lanthanum for *P*6_3_ symmetry, and their occupancies are uncorrelated. However, they mention that the framework shows a pseudo-symmetry to *P*6_3_/*m* as the symmetry breaking by *m* ^[001]^ is very small. In the study by Sansom *et al.* (2004 ▸), the neutron powder diffraction data of Ga-doped La_9.67_Si_5_GaO_26_ and La_10_Si_4_Ga_2_O_26_ were fit within the *P*6_3_/*m*, *P*6_3_, and *P* 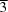 space groups, and both *P* 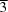 and *P*6_3_ symmetries resulted in better fits than *P*6_3_/*m* for the cation-deficient La_9.67_Si_5_GaO_26_ compound, whereas all three space groups gave similar fitting results for the stoichiometric La_10_Si_4_Ga_2_O_26_ compound. They chose the *P*6_3_/*m* space group for the La_10_Si_4_Ga_2_O_26_ compound as it is the highest symmetry space group and concluded that lowering of the space group from *P*6_3_/*m* to *P*6_3_ allows for variation in occupancy for the lanthanum site(s) as the La1 site becomes two with *P*6_3_ symmetry, whereas there is only one site for La1 in *P*6_3_/*m*; this resulted in a better fit for cation-deficient La_9.67_Si_5_GaO_26_ in *P*6_3_ symmetry. However, an *a posteriori* symmetry analysis using *SUPERFLIP* (Palatinus & van der Lee, 2008 ▸), shows that oxyapatites in this study crystallize in the *P*6_3_/*m* space group.

**Figure 1:**
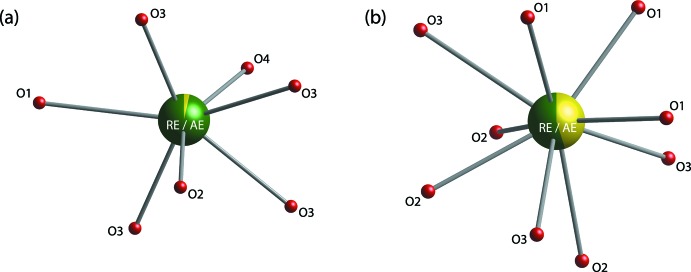
(*a*) Seven-coordinated and (*b*) nine-coordinated oxygen atoms around *RE*/*AE* cations for Ca_2_ *RE*_8_(SiO_4_)_6_O_2_.

**Figure 2:**
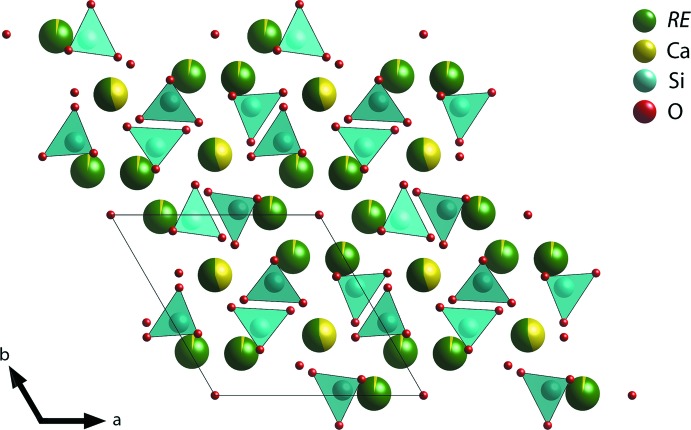
Crystal structure of Ca_2_ *RE*_8_(SiO_4_)_6_O_2_.

The cations in the *P*6_3_/*m* space group occupy Wyckoff positions of 4*f* and 6*h*. The 4*f* site is occupied by *RE* and *A* atoms coordinated by nine oxygen atoms whereas the 6*h* site is mostly occupied by *RE* coordinated by seven oxygen atoms (Fig. 1 ▸), and the isolated [SiO_4_]^4−^ tetra­hedra are linked by the cations (Fig. 2 ▸). Detailed atomic coordinates, bond lengths, and angles are given in the supporting information.

**Figure 3:**
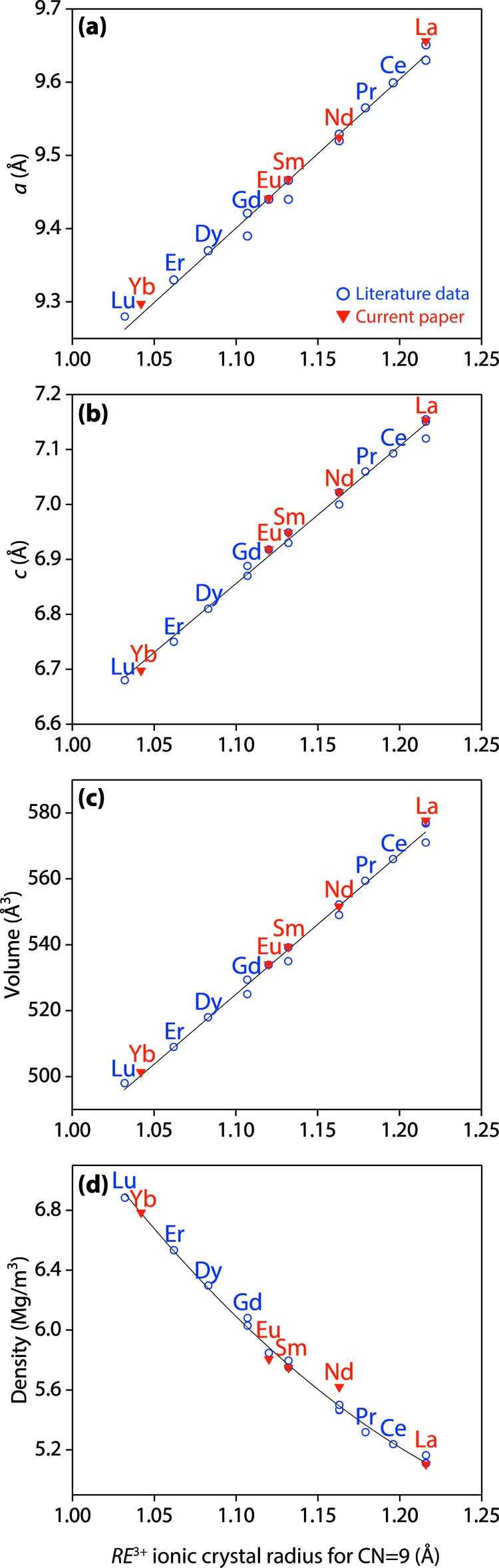
(*a*, *b*) Unit-cell parameters, (*c*) volumes, and (*d*) densities of synthesized oxyapatites compared to literature values, relative to ionic radii of nine-coordinated *RE* ^3+^ cations. Details are provided in Table 1 ▸.

The unit-cell parameters, unit-cell volumes, and densities of the synthesized Ca_2_ *RE*_8_(SiO_4_)_6_O_2_ (*RE* = La, Nd, Sm, Eu, Yb) compounds were well fit to the trendlines calculated from the previously reported values of *RE* oxyapatites (Fig. 3 ▸), and details of unit-cell parameters, cell volumes, and densities of Ca_2_ *RE*_8_(SiO_4_)_6_O_2_ from the literature and this work are provided in Table 1 ▸. The unit-cell parameters and unit-cell volumes increase linearly with increases in the ionic crystal radii (Shannon, 1976 ▸) of nine-coordinated *RE* ^3+^ cations whereas the density decreases non-linearly with the ionic crystal radii of larger nine-coordinated *RE* ^3+^ cations. The parameters of Ca-Yb oxyapatite are reported for the first time, and they match closely to predicted values shown by trendlines based on the literature data.

## Synthesis and crystallization   

The following chemicals were used as-received: Ca(NO_3_)_2_·4H_2_O (Sigma–Aldrich, ≥99%), NaNO_3_ (Sigma–Aldrich, 99.995%), tetra­ethyl orthosilicate [TEOS; Si(OC_2_H_5_)_4_; Sigma–Aldrich, ≥99%], La(NO_3_)_3_·6H_2_O (Noah Technologies, 99.9%), Nd(NO_3_)_3_·6H_2_O (Alfa Aesar, 99.9%), Sm(NO_3_)_3_·6H_2_O (Alfa Aesar, 99.9%), Eu(NO_3_)_3_·6H_2_O (Noah Technologies, 99.9%), Yb(NO_3_)_3_·6H_2_O (Alfa Aesar, 99.9%), and glacial acetic acid (CH_3_COOH; Sigma–Aldrich, 99.7%)]. For the synthesis of Ca-Nd oxyapatite, 0.06 moles of Ca(NO_3_)_2_·4H_2_O, 0.24 moles of Nd(NO_3_)_3_·6H_2_O, 80 mL of ethanol, and 80 mL of glacial acetic acid were stirred in a Pyrex beaker until the solution became clear, and then 40 mL of TEOS was added and mixed for 24 h. After 24 h of mixing, the solution was dried at 353 K for 6 d, and the dried product was heat treated at 473 K for 1 h and milled, calcined at 873 K for 4 h, then at 1273 K for 1 h and milled, and pressed into 2-cm diameter pellets using a cold isostatic press at 110 MPa. Finally, the pellets were fired at 1823 K for 8 h, and pure Ca-Nd oxyapatite was crystallized. More details of synthesis are provided elsewhere (Peterson *et al.*, 2018 ▸). For all other oxyapatites, the same procedures were used with 0.2× qu­anti­ties, and for Na-La oxyapatite, the molar ratio of Na:La in the precursors was 1:9.

**Figure 4:**
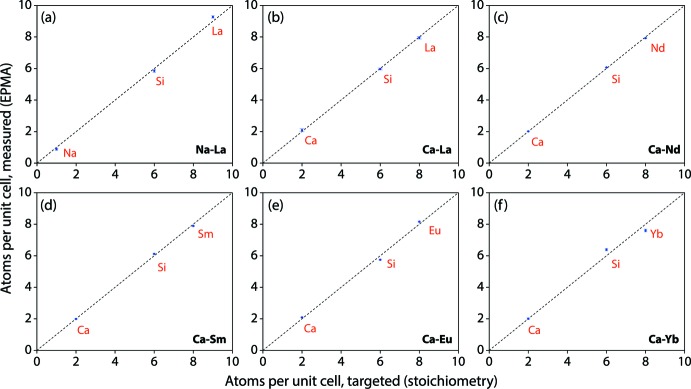
Comparison of the number of atoms per unit cell between stoichiometric and measured *A-RE* oxyapatites. Note that error bars are shown for measured values but are too small to see and, in most cases, are smaller than the size of the datapoints.

The P-XRD analysis was performed on the synthesized oxyapatites using a Bruker D8 Advance diffractometer (see Table 2 ▸ for collection parameters). The P-XRD results showed the samples to be pure oxyapatites except for the Ca-Yb compound, which also contained some Yb_2_O_3_ and Yb_2_(SiO_4_)O phases. The elemental compositions of each oxyapatite sample were measured with a JEOL 8530 Hyperprobe EPMA. Each sample was analyzed at five to eight different locations and the averages and standard deviations of these measurements are given in Table 3 ▸, on an elemental mass% basis. Because of the inaccuracy of measuring oxygen directly, it was calculated indirectly, based on target stoichiometry with the cations, using direct measurements of the cations (*i.e*., *AE*, *A*, *RE*, and Si). Table 3 ▸ also gives the molar elemental ratios of each element normalized to total atoms in the oxyapatite structure per unit cell = 42 atoms. Fig. 4 ▸ shows that the EPMA measurements confirm that samples were all on target to the batched compositions.

## Refinement   

**Figure 5:**
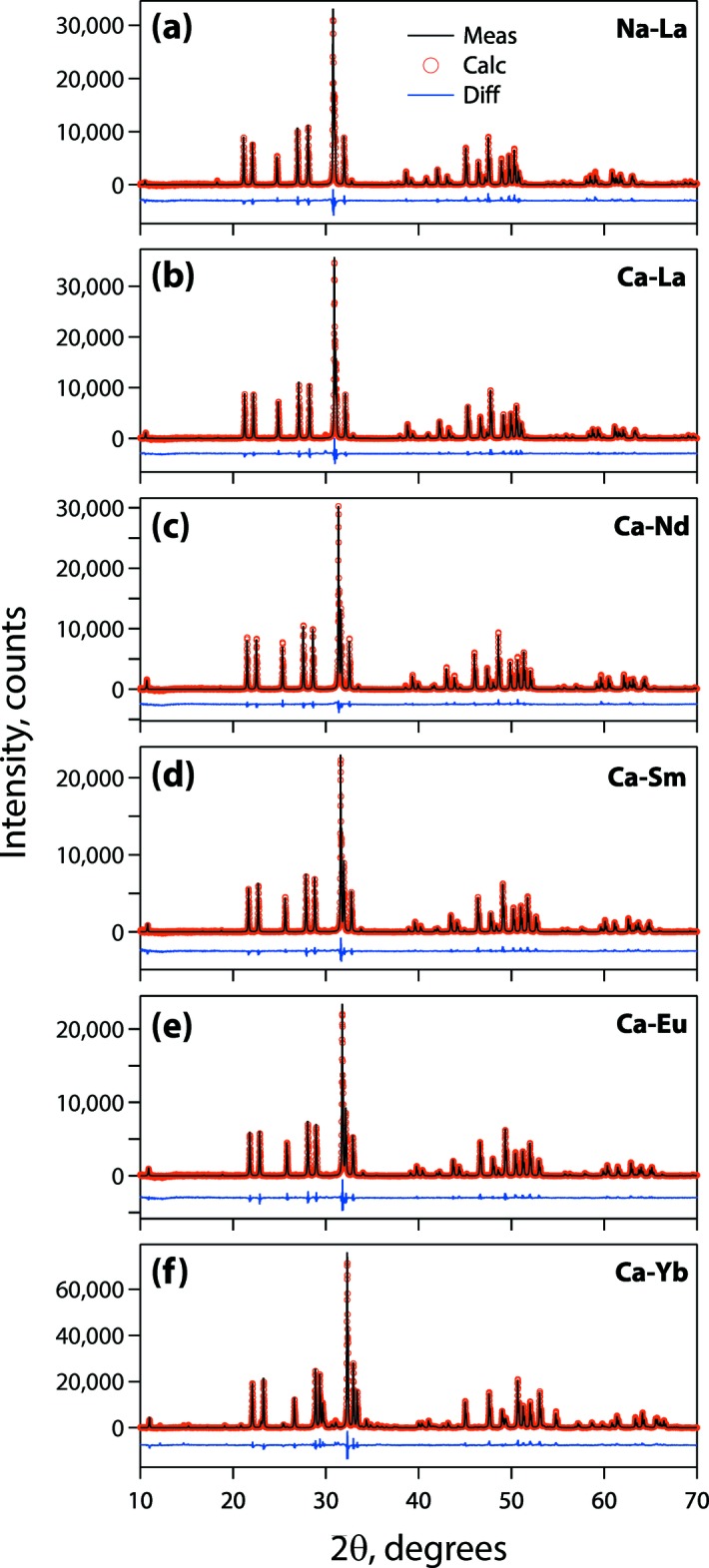
Measured, calculated, and difference XRD profiles of (*a*) NaLa_9_(SiO_4_)_6_O_2_, (*b*) Ca_2_La_8_(SiO_4_)_6_O_2_, (*c*) Ca_2_Nd_8_(SiO_4_)_6_O_2_, (*d*) Ca_2_Sm_8_(SiO_4_)_6_O_2_, (*e*) Ca_2_Eu_8_(SiO_4_)_6_O_2_, and (*f*) Ca_2_Yb_8_(SiO_4_)_6_O_2_.

Crystal data, data collection, and structure refinement details are summarized in Table 2 ▸. The Rietveld plots are shown in Fig. 5 ▸. The structures of Ca_2_ *RE*_8_(SiO_4_)_6_O_2_ (*RE* = La, Nd, Sm, Eu, Yb) and NaLa_9_(SiO_4_)_6_O_2_ were refined using the Rietveld method with *TOPAS* (version 4.2; Bruker, 2009 ▸) using the reference patterns with similar XRD profiles and chemistries as starting models. XRD patterns of the synthesized oxyapatites were similar with slight differences in peak positions and relative intensities (Fig. 5 ▸), and the reference patterns of Ca_2_Nd_8_(SiO_4_)_6_O_2_ (ICSD 62174) and NaNd_9_(SiO_4_)_6_O_2_ (ICSD 187846) were used as starting models for Ca_2_ *RE*_8_(SiO_4_)_6_O_2_ (*RE* = La, Nd, Sm, Eu, Yb) and NaLa_9_(SiO_4_)_6_O_2_, respectively. The Nd atoms in ICSD 62174 and 187846 were replaced with appropriate *RE* atoms, and the atomic positions of *RE*, Si, O, Ca, and Na atoms were refined. The occupancies of Na, Ca, and *RE* atoms at the 4*f* and 6*h* sites were not refined (values were left unchanged from the original CIF); large negative and positive densities on the cations suggest that our fixed occupancy factors are not completely correct; however, refining the occupancy factors resulted in smaller *RE*:*AE* ratio (2.4–3) or La:Na (8.4) with a slight change in *R*_wp_ (0.014–0.203%), and our EPMA analysis (Fig. 3 ▸) showed that the ratio should be 4 for *RE*:*AE* and 9 for Na-La. Therefore, we fixed the occupancy factors of the cations during the refinements. The displacement parameters were not refined and fixed to 1 Å^2^ to avoid large standard uncertainty values when refined. In addition, parameters for unit cell, scale factors, microstructure effects, and preferred orientation with spherical harmonic function (Järvinen, 1993 ▸) were refined, and the background was fitted with a Chebychev polynomial.

## Supplementary Material

Crystal structure: contains datablock(s) global, Ca-La, Ca-Nd, Ca-Sm, Ca-Eu, Ca-Yb, Na-La. DOI: 10.1107/S2056989019008442/vn2147sup1.cif

Rietveld powder data: contains datablock(s) Ca-La. DOI: 10.1107/S2056989019008442/vn2147Ca-Lasup2.rtv

Structure factors: contains datablock(s) Ca-La. DOI: 10.1107/S2056989019008442/vn2147Ca-Lasup8.hkl

Rietveld powder data: contains datablock(s) Ca-Nd. DOI: 10.1107/S2056989019008442/vn2147Ca-Ndsup3.rtv

Structure factors: contains datablock(s) Ca-Nd. DOI: 10.1107/S2056989019008442/vn2147Ca-Ndsup9.hkl

Structure factors: contains datablock(s) Ca-Sm. DOI: 10.1107/S2056989019008442/vn2147Ca-Smsup10.hkl

Rietveld powder data: contains datablock(s) Ca-Sm. DOI: 10.1107/S2056989019008442/vn2147Ca-Smsup4.rtv

Structure factors: contains datablock(s) Ca-Eu. DOI: 10.1107/S2056989019008442/vn2147Ca-Eusup11.hkl

Rietveld powder data: contains datablock(s) Ca-Eu. DOI: 10.1107/S2056989019008442/vn2147Ca-Eusup5.rtv

Structure factors: contains datablock(s) Ca-Yb. DOI: 10.1107/S2056989019008442/vn2147Ca-Ybsup12.hkl

Rietveld powder data: contains datablock(s) Ca-Yb. DOI: 10.1107/S2056989019008442/vn2147Ca-Ybsup6.rtv

Structure factors: contains datablock(s) Na-La. DOI: 10.1107/S2056989019008442/vn2147Na-Lasup13.hkl

Rietveld powder data: contains datablock(s) Na-La. DOI: 10.1107/S2056989019008442/vn2147Na-Lasup7.rtv

CCDC references: 1923003, 1923002, 1923001, 1923000, 1922999, 1922998

Additional supporting information:  crystallographic information; 3D view; checkCIF report

## Figures and Tables

**Table 1:** Summary of data on Ca_2_ *RE*_8_(SiO_4_)_6_O_2_ (*RE* = La, Ce, Pr, Nd, Sm, Eu, Gd, Dy, Er, Yb, Lu) from the current study and literature

Chemistry	*a* (Å)	*c* (Å)	Volume (Å^3^)	Density (Mg m^−3^)	Reference
Ca_2_La_8_(SiO_4_)_6_O_2_	9.6556	7.1532	578	5.105	Current study
Ca_2_La_8_(SiO_4_)_6_O_2_	9.651	7.151	577	5.112	PDF 00–029-0337
Ca_2_La_8_(SiO_4_)_6_O_2_	9.651	7.155	577	5.110	(Schroeder & Mathew, 1978 ▸)
Ca_2_La_8_(SiO_4_)_6_O_2_	9.63	7.12	571	5.165	(Ito, 1968 ▸)
Ca_2_Ce_8_(SiO_4_)_6_O_2_	9.5991	7.0928	566	5.239	(Massoni *et al.*, 2018 ▸)
Ca_2_Pr_8_(SiO_4_)_6_O_2_	9.565	7.060	559	5.319	PDF 00–029-0362
Ca_2_Nd_8_(SiO_4_)_6_O_2_	9.5241	7.0221	552	5.474	Current study
Ca_2_Nd_8_(SiO_4_)_6_O_2_	9.529	7.022	552	5.466	PDF 00–028-0228
Ca_2_Nd_8_(SiO_4_)_6_O_2_	9.5291	7.0222	552	5.467	(Fahey *et al.*, 1985 ▸)
Ca_2_Nd_8_(SiO_4_)_6_O_2_	9.52	7.00	549	5.501	(Ito, 1968 ▸)
Ca_2_Sm_8_(SiO_4_)_6_O_2_	9.4669	6.9481	539	5.749	Current study
Ca_2_Sm_8_(SiO_4_)_6_O_2_	9.466	6.949	539	5.752	PDF 00–029-0365
Ca_2_Sm_8_(SiO_4_)_6_O_2_	9.44	6.93	535	5.797	(Ito, 1968 ▸)
Ca_2_Eu_8_(SiO_4_)_6_O_2_	9.4408	6.9180	534	5.846	Current study
Ca_2_Eu_8_(SiO_4_)_6_O_2_	9.440	6.918	534	5.848	PDF 00–029-0320
Ca_2_Gd_8_(SiO_4_)_6_O_2_	9.39	6.87	525	6.081	(Ito, 1968 ▸)
Ca_2_Gd_8_(SiO_4_)_6_O_2_	9.421	6.888	529	6.030	PDF 00–028-0212
Ca_2_Dy_8_(SiO_4_)_6_O_2_	9.37	6.81	518	6.298	(Ito, 1968 ▸)
Ca_2_Er_8_(SiO_4_)_6_O_2_	9.33	6.75	509	6.534	(Ito, 1968 ▸)
Ca_2_Yb_8_(SiO_4_)_6_O_2_	9.2974	6.6975	501	6.785	Current study
Ca_2_Lu_8_(SiO_4_)_6_O_2_	9.28	6.68	498	6.884	(Ito, 1968 ▸)

**Table:** 

	Ca-La	Ca-Nd	Ca-Sm
Crystal data			
Chemical formula	Ca_2_La_8_(SiO_4_)_6_O_2_	Ca_2_Nd_8_(SiO_4_)_6_O_2_	Ca_2_Sm_8_(SiO_4_)_6_O_2_
*M*_r_	1775.7	1818.4	1867.3
Crystal system, space group	Hexagonal, *P*6_3_/*m*	Hexagonal, *P*6_3_/*m*	Hexagonal, *P*6_3_/*m*
Temperature (K)	295	295	295
*a*, *c* (Å)	9.65568 (7), 7.15323 (6)	9.52414 (5), 7.02213 (5)	9.46696 (6), 6.94810 (5)
*V* (Å^3^)	577.56 (1)	551.63 (1)	539.28 (1)
*Z*	1	1	1
Radiation type	Cu *K*α, λ = 1.54188 Å	Cu *K*α, λ = 1.54188 Å	Cu *K*α, λ = 1.54188 Å
Specimen shape, size (mm)	Flat sheet, 25 × 25	Flat sheet, 25 × 25	Flat sheet, 25 × 25

Data collection			
Diffractometer	Bruker D8 Advance	Bruker D8 Advance	Bruker D8 Advance
Specimen mounting	Packed powder pellet	Packed powder pellet	Packed powder pellet
Data collection mode	Reflection	Reflection	Reflection
Scan method	Step	Step	Step
2θ values (°)	2θ_min_ = 10 2θ_max_ = 70 2θ_step_ = 0.009	2θ_min_ = 10 2θ_max_ = 70 2θ_step_ = 0.009	2θ_min_ = 10 2θ_max_ = 70 2θ_step_ = 0.009

Refinement			
*R* factors and goodness of fit	*R*_p_ = 0.05, *R*_wp_ = 0.07, *R*_exp_ = 0.03, *R*_Bragg_ = 0.03, χ^2^ = 5.617	*R*_p_ = 0.05, *R*_wp_ = 0.06, *R*_exp_ = 0.03, *R*_Bragg_ = 0.03, χ^2^ = 4.452	*R*_p_ = 0.04, *R*_wp_ = 0.06, *R*_exp_ = 0.03, *R*_Bragg_ = 0.04, χ^2^ = 3.386
No. of parameters	24	26	26

**Table:** 

	Ca-Eu	Ca-Yb	Na-La
Crystal data			
Chemical formula	Ca_2_Eu_8_(SiO_4_)_6_O_2_	Ca_2_Yb_8_(SiO_4_)_6_O_2_	NaLa_9_(SiO_4_)_6_O_2_
*M*_r_	1880.1	2048.7	1857.63
Crystal system, space group	Hexagonal, *P*6_3_/*m*	Hexagonal, *P*6_3_/*m*	Hexagonal, *P*6_3_/*m*
Temperature (K)	295	295	295
*a*, *c* (Å)	9.44082 (7), 6.91804 (6)	9.29743 (7), 6.69748 (6)	9.69061 (7), 7.18567 (6)
*V* (Å^3^)	533.99 (1)	501.38 (1)	584.39 (1)
*Z*	1	1	1
Radiation type	Cu *K*α, λ = 1.54188 Å	Cu *K*α, λ = 1.54188 Å	Cu *K*α, λ = 1.54188 Å
Specimen shape, size (mm)	Flat sheet, 25 × 25	Flat sheet, 25 × 25	Flat sheet, 25 × 25

Data collection			
Diffractometer	Bruker D8 Advance	Bruker D8 Advance	Bruker D8 Advance
Specimen mounting	Packed powder pellet	Packed powder pellet	Packed powder pellet
Data collection mode	Reflection	Reflection	Reflection
Scan method	Step	Step	Step
2θ values (°)	2θ_min_ = 10 2θ_max_ = 70 2θ_step_ = 0.009	2θ_min_ = 10 2θ_max_ = 70 2θ_step_ = 0.009	2θ_min_ = 10 2θ_max_ = 70 2θ_step_ = 0.009

Refinement			
*R* factors and goodness of fit	*R*_p_ = 0.04, *R*_wp_ = 0.06, *R*_exp_ = 0.03, *R*_Bragg_ = 0.03, χ^2^ = 3.842	*R*_p_ = 0.05, *R*_wp_ = 0.07, *R*_exp_ = 0.02, *R*_Bragg_ = 0.04, χ^2^ = 14.062	*R*_p_ = 0.04, *R*_wp_ = 0.06, *R*_exp_ = 0.03, *R*_Bragg_ = 0.05, χ^2^ = 6.150
No. of parameters	26	36	29

Computer programs: *XRD Commander* (Kienle & Jacob, 2003 ▸), *TOPAS* (Bruker, 2009 ▸), *VESTA* (Momma & Izumi, 2011 ▸) and *publCIF* (Westrip, 2010 ▸).

**Table 3:** EPMA measurements for oxyapatite samples *A*_*x*_ *RE*_10 − *x*_(SiO_4_)_6_O_2_ (*A* = Ca or Na, *RE* = La, Ce, Nd, Sm or Yb) synthesized in this study Measurements shown as mean with standard deviations in parenthesis of including both mass% and as atoms in the unit cell normalized to 42; oxygen was calculated based on stoichiometry in both cases.

Sample ID	Mean (std devn)	*A*	*RE*	Si	O (calc. stoch.)	Total
Ca_2_La_8_Si_6_O_26_	Mass %	4.74 (0.18)	62.45 (0.98)	9.48 (0.21)	23.48 (1/3)	100.15 (1.29)
	Atoms	2.09 (0.10)	7.96 (0.08	5.97 (0.05)	25.98 (0.03)	42
Ca_2_Nd_8_Si_6_O_26_	Mass %	4.47 (0.04)	63.34 (1/4)	9.44 (0.05)	23.08 (0.06)	100.33 (0.28)
	Atoms	2.01 (0.02)	7.92 (0.03)	6.06 (0.02)	26.01 (0.01)	42
Ca_2_Sm_8_Si_6_O_26_	Mass %	4.42 (0.03)	65.72 (0.34)	9.51 (0.05)	23.09 (0.05)	102.75 (0.34)
	Atoms	1.99 (0.01)	7.88 (0.04)	6.11 (0.03)	26.03 (0.01)	42
Ca_2_Eu_8_Si_6_O_26_	Mass %	4.38 (0.09)	63.31 (1.09)	8.35 (0.03)	21.26 (0.12)	97.29 (1.11)
	Atoms	2.13 (0.06)	8.13 (0.10)	5.80 (0.04)	25.93 (0.004)	42
Ca_2_Yb_8_Si_6_O_26_	Mass %	4.10 (0.10)	67.01 (1.15)	9.14 (0.17)	21.35 (0.29)	101.59 (1.45)
	Atoms	2.00 (0.05)	7.57 (0.08)	6.36 (0.07)	26.07 (0.02)	42
NaLa_9_Si_6_O_26_	Mass %	1.08 (0.08)	68.46 (0.41)	8.73 (0.16)	22.15 (0.15)	100.43 (0.37)
	Atoms	0.88 (0.07)	9.26 (0.08)	5.84 (0.08)	26.02 (0.04)	42

## supplementary crystallographic information

### Calcium lanthanum silicate oxyapatite (Ca-La) Crystal data

**Table:**

Ca_2_La_8_(SiO_4_)_6_O_2_	*Z* = 1
*M**_r_* = 1775.7	*D*_x_ = 5.105 Mg m^−^^3^
Hexagonal, *P*6_3_/*m*	Cu *K*α radiation, λ = 1.54188 Å
*a* = 9.65568 (7) Å	*T* = 295 K
*c* = 7.15323 (6) Å	white
*V* = 577.56 (1) Å^3^	flat_sheet, 25 × 25 mm

### Calcium lanthanum silicate oxyapatite (Ca-La) Data collection

**Table:**

Bruker D8 Advance diffractometer	Data collection mode: reflection
Radiation source: sealed X-ray tube	Scan method: step
Specimen mounting: packed powder pellet	2θ_min_ = 10°, 2θ_max_ = 70°, 2θ_step_ = 0.009°

### Calcium lanthanum silicate oxyapatite (Ca-La) Refinement

**Table:**

*R*_p_ = 0.05	Profile function: pseudo-Voigt
*R*_wp_ = 0.07	24 parameters
*R*_exp_ = 0.03	Weighting scheme based on measured s.u.'s
*R*_Bragg_ = 0.03	(Δ/σ)_max_ < 0.001
6994 data points	Background function: Chebychev

### Calcium lanthanum silicate oxyapatite (Ca-La) Special details

**Table:**

Refinement. Beq were fixed as 1Å squared during refinement as they result high standard uncertainties

### Calcium lanthanum silicate oxyapatite (Ca-La) Fractional atomic coordinates and isotropic or equivalent isotropic displacement parameters (Å^2^)

**Table:**

	*x*	*y*	*z*	*U*_iso_*/*U*_eq_	Occ. (<1)
Ca1	0.333333	0.666667	−0.0033 (4)	0.0127*	0.448
La1	0.333333	0.666667	−0.0033 (4)	0.0127*	0.552
Ca2	0.23167 (12)	−0.01384 (16)	0.25	0.0127*	0.035
La2	0.23167 (12)	−0.01384 (16)	0.25	0.0127*	0.965
Si1	0.4033 (6)	0.3747 (7)	0.25	0.0127*	
O1	0.3275 (10)	0.4848 (10)	0.25	0.0127*	
O2	0.5952 (12)	0.4633 (10)	0.25	0.0127*	
O3	0.3415 (6)	0.2571 (6)	0.0722 (7)	0.0127*	
O4	0	0	0.25	0.0127*	

### Calcium lanthanum silicate oxyapatite (Ca-La) Atomic displacement parameters (Å^2^)

**Table:**

	*U*^11^	*U*^22^	*U*^33^	*U*^12^	*U*^13^	*U*^23^
?	?	?	?	?	?	?

### Calcium lanthanum silicate oxyapatite (Ca-La) Geometric parameters (Å, º)

**Table:**

Ca1—Ca1^i^	3.529 (4)	La1—O2^vii^	2.456 (8)
Ca1—Ca1^ii^	3.624 (4)	La1—O2^viii^	2.456 (10)
Ca1—La1	0	Ca2—Ca2^iv^	3.995 (3)
Ca1—La1^i^	3.529 (4)	Ca2—Ca2^xi^	3.995 (2)
Ca1—La1^ii^	3.624 (4)	Ca2—La2	0
Ca1—Ca2^iii^	4.098 (2)	Ca2—La2^iv^	3.995 (3)
Ca1—Ca2^iv^	4.0978 (18)	Ca2—La2^xi^	3.995 (2)
Ca1—Ca2^v^	4.098 (2)	Ca2—Si1	3.256 (6)
Ca1—Si1^vi^	3.280 (6)	Ca2—O4	2.3067 (14)
Ca1—Si1^vii^	3.280 (8)	La2—O2^xii^	2.552 (9)
Ca1—Si1^viii^	3.280 (5)	La2—O3^xiii^	2.450 (6)
La1—La1^i^	3.529 (4)	La2—O3^xiv^	2.450 (6)
La1—La1^ii^	3.624 (4)	La2—O4	2.3067 (14)
La1—O1	2.504 (8)	Si1—O1	1.563 (14)
La1—O1^ix^	2.504 (6)	Si1—O2	1.606 (12)
La1—O1^x^	2.504 (10)	Si1—O3	1.608 (6)
La1—O2^vi^	2.456 (9)	Si1—O3^ii^	1.608 (6)
			
Ca1^i^—Ca1—Ca1^ii^	180	O2^vi^—La1—O2^vii^	74.1 (4)
Ca1^i^—Ca1—La1	0	O2^vi^—La1—O2^viii^	74.1 (4)
Ca1^i^—Ca1—La1^i^	0	O2^vii^—La1—O2^viii^	74.1 (3)
Ca1^i^—Ca1—La1^ii^	180	Ca1^xv^—Ca2—Ca1^xvi^	52.48 (5)
Ca1^i^—Ca1—Ca2^iii^	116.24 (4)	Ca1^xv^—Ca2—Ca2^iv^	150.60 (4)
Ca1^i^—Ca1—Ca2^iv^	116.24 (4)	Ca1^xv^—Ca2—Ca2^xi^	104.54 (3)
Ca1^i^—Ca1—Ca2^v^	116.24 (4)	Ca1^xv^—Ca2—La2	0
Ca1^i^—Ca1—Si1^vi^	57.45 (8)	Ca1^xv^—Ca2—La2^iv^	150.60 (4)
Ca1^i^—Ca1—Si1^vii^	57.45 (10)	Ca1^xv^—Ca2—La2^xi^	104.54 (3)
Ca1^i^—Ca1—Si1^viii^	57.45 (7)	Ca1^xv^—Ca2—Si1	133.78 (10)
Ca1^ii^—Ca1—La1	0	Ca1^xv^—Ca2—O4	130.38 (3)
Ca1^ii^—Ca1—La1^i^	180	Ca1^xvi^—Ca2—Ca2^iv^	150.60 (4)
Ca1^ii^—Ca1—La1^ii^	0	Ca1^xvi^—Ca2—Ca2^xi^	104.54 (3)
Ca1^ii^—Ca1—Ca2^iii^	63.76 (4)	Ca1^xvi^—Ca2—La2	0
Ca1^ii^—Ca1—Ca2^iv^	63.76 (4)	Ca1^xvi^—Ca2—La2^iv^	150.60 (4)
Ca1^ii^—Ca1—Ca2^v^	63.76 (4)	Ca1^xvi^—Ca2—La2^xi^	104.54 (3)
Ca1^ii^—Ca1—Si1^vi^	122.55 (8)	Ca1^xvi^—Ca2—Si1	133.78 (10)
Ca1^ii^—Ca1—Si1^vii^	122.55 (10)	Ca1^xvi^—Ca2—O4	130.38 (3)
Ca1^ii^—Ca1—Si1^viii^	122.55 (7)	Ca2^iv^—Ca2—Ca2^xi^	60.00 (3)
La1—Ca1—La1^i^	0	Ca2^iv^—Ca2—La2	0
La1—Ca1—La1^ii^	0	Ca2^iv^—Ca2—La2^iv^	0
La1—Ca1—Ca2^iii^	0	Ca2^iv^—Ca2—La2^xi^	60.00 (3)
La1—Ca1—Ca2^iv^	0	Ca2^iv^—Ca2—Si1	53.27 (12)
La1—Ca1—Ca2^v^	0	Ca2^iv^—Ca2—O4	30.00 (3)
La1—Ca1—Si1^vi^	0	Ca2^xi^—Ca2—La2	0
La1—Ca1—Si1^vii^	0	Ca2^xi^—Ca2—La2^iv^	60.00 (3)
La1—Ca1—Si1^viii^	0	Ca2^xi^—Ca2—La2^xi^	0
La1^i^—Ca1—La1^ii^	180	Ca2^xi^—Ca2—Si1	113.27 (12)
La1^i^—Ca1—Ca2^iii^	116.24 (4)	Ca2^xi^—Ca2—O4	30.00 (2)
La1^i^—Ca1—Ca2^iv^	116.24 (4)	La2—Ca2—La2^iv^	0
La1^i^—Ca1—Ca2^v^	116.24 (4)	La2—Ca2—La2^xi^	0
La1^i^—Ca1—Si1^vi^	57.45 (8)	La2—Ca2—Si1	0
La1^i^—Ca1—Si1^vii^	57.45 (10)	La2—Ca2—O4	0
La1^i^—Ca1—Si1^viii^	57.45 (7)	La2^iv^—Ca2—La2^xi^	60.00 (3)
La1^ii^—Ca1—Ca2^iii^	63.76 (4)	La2^iv^—Ca2—Si1	53.27 (12)
La1^ii^—Ca1—Ca2^iv^	63.76 (4)	La2^iv^—Ca2—O4	30.00 (3)
La1^ii^—Ca1—Ca2^v^	63.76 (4)	La2^xi^—Ca2—Si1	113.27 (12)
La1^ii^—Ca1—Si1^vi^	122.55 (8)	La2^xi^—Ca2—O4	30.00 (2)
La1^ii^—Ca1—Si1^vii^	122.55 (10)	Si1—Ca2—O4	83.27 (12)
La1^ii^—Ca1—Si1^viii^	122.55 (7)	Ca2—La2—Ca2^iv^	0
Ca2^iii^—Ca1—Ca2^iv^	101.93 (5)	Ca2—La2—Ca2^xi^	0
Ca2^iii^—Ca1—Ca2^v^	101.93 (5)	Ca2—La2—O2^xii^	0
Ca2^iii^—Ca1—Si1^vi^	145.27 (10)	Ca2—La2—O3^xiii^	0
Ca2^iii^—Ca1—Si1^vii^	61.97 (11)	Ca2—La2—O3^xiv^	0
Ca2^iii^—Ca1—Si1^viii^	111.22 (16)	Ca2—La2—O4	0
Ca2^iv^—Ca1—Ca2^v^	101.93 (5)	Ca2^iv^—La2—Ca2^xi^	60.00 (3)
Ca2^iv^—Ca1—Si1^vi^	111.22 (11)	Ca2^iv^—La2—O2^xii^	118.6 (4)
Ca2^iv^—Ca1—Si1^vii^	145.27 (11)	Ca2^iv^—La2—O3^xiii^	109.4 (2)
Ca2^iv^—Ca1—Si1^viii^	61.97 (5)	Ca2^iv^—La2—O3^xiv^	109.4 (2)
Ca2^v^—Ca1—Si1^vi^	61.97 (6)	Ca2^iv^—La2—O4	30.00 (3)
Ca2^v^—Ca1—Si1^vii^	111.22 (10)	Ca2^xi^—La2—O2^xii^	178.6 (4)
Ca2^v^—Ca1—Si1^viii^	145.27 (16)	Ca2^xi^—La2—O3^xiii^	95.92 (12)
Si1^vi^—Ca1—Si1^vii^	93.77 (17)	Ca2^xi^—La2—O3^xiv^	95.92 (12)
Si1^vi^—Ca1—Si1^viii^	93.77 (16)	Ca2^xi^—La2—O4	30.00 (2)
Si1^vii^—Ca1—Si1^viii^	93.77 (14)	O2^xii^—La2—O3^xiii^	84.54 (17)
Ca1—La1—Ca1^i^	0	O2^xii^—La2—O3^xiv^	84.54 (17)
Ca1—La1—Ca1^ii^	0	O2^xii^—La2—O4	148.6 (4)
Ca1—La1—La1^i^	0	O3^xiii^—La2—O3^xiv^	140.3 (3)
Ca1—La1—La1^ii^	0	O3^xiii^—La2—O4	104.54 (16)
Ca1—La1—O1	0	O3^xiv^—La2—O4	104.54 (16)
Ca1—La1—O1^ix^	0	Ca1^xvii^—Si1—Ca1^xviii^	65.10 (15)
Ca1—La1—O1^x^	0	Ca1^xvii^—Si1—Ca2	80.71 (16)
Ca1—La1—O2^vi^	0	Ca1^xvii^—Si1—O1	137.6 (2)
Ca1—La1—O2^vii^	0	Ca1^xvii^—Si1—O2	46.1 (3)
Ca1—La1—O2^viii^	0	Ca1^xvii^—Si1—O3	112.1 (4)
Ca1^i^—La1—Ca1^ii^	180	Ca1^xvii^—Si1—O3^ii^	61.6 (3)
Ca1^i^—La1—La1^i^	0	Ca1^xviii^—Si1—Ca2	80.71 (16)
Ca1^i^—La1—La1^ii^	180	Ca1^xviii^—Si1—O1	137.6 (2)
Ca1^i^—La1—O1	136.35 (18)	Ca1^xviii^—Si1—O2	46.1 (3)
Ca1^i^—La1—O1^ix^	136.35 (14)	Ca1^xviii^—Si1—O3	61.6 (3)
Ca1^i^—La1—O1^x^	136.3 (2)	Ca1^xviii^—Si1—O3^ii^	112.1 (4)
Ca1^i^—La1—O2^vi^	44.1 (2)	Ca2—Si1—O1	129.9 (3)
Ca1^i^—La1—O2^vii^	44.1 (2)	Ca2—Si1—O2	113.6 (5)
Ca1^i^—La1—O2^viii^	44.1 (2)	Ca2—Si1—O3	52.5 (3)
Ca1^ii^—La1—La1^i^	180	Ca2—Si1—O3^ii^	52.5 (3)
Ca1^ii^—La1—La1^ii^	0	O1—Si1—O2	116.5 (6)
Ca1^ii^—La1—O1	43.65 (18)	O1—Si1—O3	110.2 (4)
Ca1^ii^—La1—O1^ix^	43.65 (14)	O1—Si1—O3^ii^	110.2 (4)
Ca1^ii^—La1—O1^x^	43.7 (2)	O2—Si1—O3	107.3 (4)
Ca1^ii^—La1—O2^vi^	135.9 (2)	O2—Si1—O3^ii^	107.3 (4)
Ca1^ii^—La1—O2^vii^	135.9 (2)	O3—Si1—O3^ii^	104.6 (4)
Ca1^ii^—La1—O2^viii^	135.9 (2)	La1—O1—La1^ii^	92.7 (4)
La1^i^—La1—La1^ii^	180	La1—O1—Si1	128.5 (3)
La1^i^—La1—O1	136.35 (18)	La1^ii^—O1—Si1	128.5 (3)
La1^i^—La1—O1^ix^	136.35 (14)	La1^xvii^—O2—La1^xviii^	91.9 (4)
La1^i^—La1—O1^x^	136.3 (2)	La1^xvii^—O2—La2^v^	115.4 (2)
La1^i^—La1—O2^vi^	44.1 (2)	La1^xvii^—O2—Si1	105.8 (3)
La1^i^—La1—O2^vii^	44.1 (2)	La1^xviii^—O2—La2^v^	115.4 (2)
La1^i^—La1—O2^viii^	44.1 (2)	La1^xviii^—O2—Si1	105.8 (3)
La1^ii^—La1—O1	43.65 (18)	La2^v^—O2—Si1	118.9 (7)
La1^ii^—La1—O1^ix^	43.65 (14)	La2^viii^—O3—Si1	143.4 (4)
La1^ii^—La1—O1^x^	43.7 (2)	Ca2—O4—Ca2^iv^	120.00 (5)
La1^ii^—La1—O2^vi^	135.9 (2)	Ca2—O4—Ca2^xi^	120.00 (6)
La1^ii^—La1—O2^vii^	135.9 (2)	Ca2—O4—La2	0
La1^ii^—La1—O2^viii^	135.9 (2)	Ca2—O4—La2^iv^	120.00 (5)
O1—La1—O1^ix^	73.4 (3)	Ca2—O4—La2^xi^	120.00 (6)
O1—La1—O1^x^	73.4 (3)	Ca2^iv^—O4—Ca2^xi^	120.00 (6)
O1—La1—O2^vi^	94.3 (3)	Ca2^iv^—O4—La2	120.00 (5)
O1—La1—O2^vii^	154.0 (3)	Ca2^iv^—O4—La2^iv^	0
O1—La1—O2^viii^	125.9 (3)	Ca2^iv^—O4—La2^xi^	120.00 (6)
O1^ix^—La1—O1^x^	73.4 (3)	Ca2^xi^—O4—La2	120.00 (6)
O1^ix^—La1—O2^vi^	125.9 (4)	Ca2^xi^—O4—La2^iv^	120.00 (6)
O1^ix^—La1—O2^vii^	94.3 (2)	Ca2^xi^—O4—La2^xi^	0
O1^ix^—La1—O2^viii^	154.0 (5)	La2—O4—La2^iv^	120.00 (5)
O1^x^—La1—O2^vi^	154.0 (2)	La2—O4—La2^xi^	120.00 (6)
O1^x^—La1—O2^vii^	125.9 (4)	La2^iv^—O4—La2^xi^	120.00 (6)
O1^x^—La1—O2^viii^	94.3 (4)		

Symmetry codes: (i) *x*, *y*, −*z*−1/2; (ii) *x*, *y*, −*z*+1/2; (iii) *x*, *y*+1, *z*; (iv) −*y*, *x*−*y*, *z*; (v) −*x*+*y*+1, −*x*+1, *z*; (vi) −*x*+1, −*y*+1, *z*−1/2; (vii) *y*, −*x*+*y*+1, *z*−1/2; (viii) *x*−*y*, *x*, *z*−1/2; (ix) −*y*+1, *x*−*y*+1, *z*; (x) −*x*+*y*, −*x*+1, *z*; (xi) −*x*+*y*, −*x*, *z*; (xii) −*y*+1, *x*−*y*, *z*; (xiii) *y*, −*x*+*y*, *z*+1/2; (xiv) *y*, −*x*+*y*, −*z*; (xv) *x*, *y*−1, *z*; (xvi) *x*, *y*−1, −*z*+1/2; (xvii) −*x*+1, −*y*+1, *z*+1/2; (xviii) −*x*+1, −*y*+1, −*z*.

### Calcium neodymium silicate oxyapatite (Ca-Nd) Crystal data

**Table:**

Ca_2_Nd_8_(SiO_4_)_6_O_2_	*Z* = 1
*M**_r_* = 1818.4	*D*_x_ = 5.474 Mg m^−^^3^
Hexagonal, *P*6_3_/*m*	Cu *K*α radiation, λ = 1.54188 Å
*a* = 9.52414 (5) Å	*T* = 295 K
*c* = 7.02213 (5) Å	blue_violet
*V* = 551.63 (1) Å^3^	flat_sheet, 25 × 25 mm

### Calcium neodymium silicate oxyapatite (Ca-Nd) Data collection

**Table:**

Bruker D8 Advance diffractometer	Data collection mode: reflection
Radiation source: sealed X-ray tube	Scan method: step
Specimen mounting: packed powder pellet	2θ_min_ = 10°, 2θ_max_ = 70°, 2θ_step_ = 0.009°

### Calcium neodymium silicate oxyapatite (Ca-Nd) Refinement

**Table:**

*R*_p_ = 0.05	26 parameters
*R*_wp_ = 0.06	Weighting scheme based on measured s.u.'s
*R*_exp_ = 0.03	(Δ/σ)_max_ = 0.001
*R*_Bragg_ = 0.03	Background function: Chebychev
6994 data points	Preferred orientation correction: spherical harmonic
Profile function: pseudo-Voigt	

### Calcium neodymium silicate oxyapatite (Ca-Nd) Special details

**Table:**

Refinement. Beq were fixed as 1Å squared during refinement as they result high errors

### Calcium neodymium silicate oxyapatite (Ca-Nd) Fractional atomic coordinates and isotropic or equivalent isotropic displacement parameters (Å^2^)

**Table:**

	*x*	*y*	*z*	*U*_iso_*/*U*_eq_	Occ. (<1)
Ca1	0.333333	0.666667	−0.0015 (6)	0.0127*	0.448
Nd1	0.333333	0.666667	−0.0015 (6)	0.0127*	0.552
Ca2	0.23298 (13)	−0.0105 (2)	0.25	0.0127*	0.035
Nd2	0.23298 (13)	−0.0105 (2)	0.25	0.0127*	0.965
Si1	0.4017 (6)	0.3747 (7)	0.25	0.0127*	
O1	0.3225 (11)	0.4858 (11)	0.25	0.0127*	
O2	0.5984 (12)	0.4765 (11)	0.25	0.0127*	
O3	0.3343 (6)	0.2513 (7)	0.0773 (7)	0.0127*	
O4	0	0	0.25	0.0127*	

### Calcium neodymium silicate oxyapatite (Ca-Nd) Atomic displacement parameters (Å^2^)

**Table:**

	*U*^11^	*U*^22^	*U*^33^	*U*^12^	*U*^13^	*U*^23^
?	?	?	?	?	?	?

### Calcium neodymium silicate oxyapatite (Ca-Nd) Geometric parameters (Å, º)

**Table:**

Ca1—Ca1^i^	3.490 (6)	Ca2—Ca2^iv^	3.933 (3)
Ca1—Ca1^ii^	3.532 (6)	Ca2—Ca2^xi^	3.933 (2)
Ca1—Nd1	0	Ca2—Nd2	0
Ca1—Nd1^i^	3.490 (6)	Ca2—Nd2^iv^	3.933 (3)
Ca1—Nd1^ii^	3.532 (6)	Ca2—Nd2^xi^	3.933 (2)
Ca1—Ca2^iii^	4.053 (3)	Ca2—Si1	3.185 (6)
Ca1—Ca2^iv^	4.053 (2)	Ca2—Si1^xi^	3.284 (5)
Ca1—Ca2^v^	4.053 (3)	Ca2—O2^xii^	2.399 (9)
Ca1—Si1^vi^	3.250 (6)	Ca2—O3^xiii^	2.431 (6)
Ca1—Si1^vii^	3.250 (8)	Ca2—O3^xiv^	2.431 (6)
Ca1—Si1^viii^	3.250 (5)	Ca2—O4	2.2706 (16)
Ca1—O1	2.433 (8)	Nd2—O2^xii^	2.399 (9)
Ca1—O1^ix^	2.433 (7)	Nd2—O3^xiii^	2.431 (6)
Ca1—O1^x^	2.433 (11)	Nd2—O3^xiv^	2.431 (6)
Nd1—Nd1^i^	3.490 (6)	Nd2—O4	2.2706 (16)
Nd1—Nd1^ii^	3.532 (6)	Si1—O1	1.577 (15)
Nd1—O1	2.433 (8)	Si1—O2	1.623 (11)
Nd1—O1^ix^	2.433 (7)	Si1—O3	1.584 (6)
Nd1—O1^x^	2.433 (11)	Si1—O3^ii^	1.584 (6)
			
Ca1^i^—Ca1—Ca1^ii^	180	Ca1^xvi^—Ca2—O3^xiii^	45.2 (2)
Ca1^i^—Ca1—Nd1	0	Ca1^xvi^—Ca2—O3^xiv^	96.8 (2)
Ca1^i^—Ca1—Nd1^i^	0	Ca1^xvi^—Ca2—O4	129.78 (4)
Ca1^i^—Ca1—Nd1^ii^	180	Ca2^iv^—Ca2—Ca2^xi^	60.00 (4)
Ca1^i^—Ca1—Ca2^iii^	115.83 (6)	Ca2^iv^—Ca2—Nd2	0
Ca1^i^—Ca1—Ca2^iv^	115.83 (5)	Ca2^iv^—Ca2—Nd2^iv^	0
Ca1^i^—Ca1—Ca2^v^	115.83 (6)	Ca2^iv^—Ca2—Nd2^xi^	60.00 (4)
Ca1^i^—Ca1—Si1^vi^	57.53 (9)	Ca2^iv^—Ca2—Si1	53.72 (12)
Ca1^i^—Ca1—Si1^vii^	57.53 (11)	Ca2^iv^—Ca2—Si1^xi^	111.43 (17)
Ca1^i^—Ca1—Si1^viii^	57.53 (8)	Ca2^iv^—Ca2—O2^xii^	120.7 (4)
Ca1^i^—Ca1—O1	136.5 (2)	Ca2^iv^—Ca2—O3^xiii^	108.2 (2)
Ca1^i^—Ca1—O1^ix^	136.54 (17)	Ca2^iv^—Ca2—O3^xiv^	108.2 (2)
Ca1^i^—Ca1—O1^x^	136.5 (3)	Ca2^iv^—Ca2—O4	30.00 (4)
Ca1^ii^—Ca1—Nd1	0	Ca2^xi^—Ca2—Nd2	0
Ca1^ii^—Ca1—Nd1^i^	180	Ca2^xi^—Ca2—Nd2^iv^	60.00 (4)
Ca1^ii^—Ca1—Nd1^ii^	0	Ca2^xi^—Ca2—Nd2^xi^	0
Ca1^ii^—Ca1—Ca2^iii^	64.17 (6)	Ca2^xi^—Ca2—Si1	113.72 (13)
Ca1^ii^—Ca1—Ca2^iv^	64.17 (5)	Ca2^xi^—Ca2—Si1^xi^	51.43 (17)
Ca1^ii^—Ca1—Ca2^v^	64.17 (6)	Ca2^xi^—Ca2—O2^xii^	179.3 (4)
Ca1^ii^—Ca1—Si1^vi^	122.47 (9)	Ca2^xi^—Ca2—O3^xiii^	94.26 (14)
Ca1^ii^—Ca1—Si1^vii^	122.47 (11)	Ca2^xi^—Ca2—O3^xiv^	94.26 (14)
Ca1^ii^—Ca1—Si1^viii^	122.47 (8)	Ca2^xi^—Ca2—O4	30.00 (3)
Ca1^ii^—Ca1—O1	43.5 (2)	Nd2—Ca2—Nd2^iv^	0
Ca1^ii^—Ca1—O1^ix^	43.46 (17)	Nd2—Ca2—Nd2^xi^	0
Ca1^ii^—Ca1—O1^x^	43.5 (3)	Nd2—Ca2—Si1	0
Nd1—Ca1—Nd1^i^	0	Nd2—Ca2—Si1^xi^	0
Nd1—Ca1—Nd1^ii^	0	Nd2—Ca2—O2^xii^	0
Nd1—Ca1—Ca2^iii^	0	Nd2—Ca2—O3^xiii^	0
Nd1—Ca1—Ca2^iv^	0	Nd2—Ca2—O3^xiv^	0
Nd1—Ca1—Ca2^v^	0	Nd2—Ca2—O4	0
Nd1—Ca1—Si1^vi^	0	Nd2^iv^—Ca2—Nd2^xi^	60.00 (4)
Nd1—Ca1—Si1^vii^	0	Nd2^iv^—Ca2—Si1	53.72 (12)
Nd1—Ca1—Si1^viii^	0	Nd2^iv^—Ca2—Si1^xi^	111.43 (17)
Nd1—Ca1—O1	0	Nd2^iv^—Ca2—O2^xii^	120.7 (4)
Nd1—Ca1—O1^ix^	0	Nd2^iv^—Ca2—O3^xiii^	108.2 (2)
Nd1—Ca1—O1^x^	0	Nd2^iv^—Ca2—O3^xiv^	108.2 (2)
Nd1^i^—Ca1—Nd1^ii^	180	Nd2^iv^—Ca2—O4	30.00 (4)
Nd1^i^—Ca1—Ca2^iii^	115.83 (6)	Nd2^xi^—Ca2—Si1	113.72 (13)
Nd1^i^—Ca1—Ca2^iv^	115.83 (5)	Nd2^xi^—Ca2—Si1^xi^	51.43 (17)
Nd1^i^—Ca1—Ca2^v^	115.83 (6)	Nd2^xi^—Ca2—O2^xii^	179.3 (4)
Nd1^i^—Ca1—Si1^vi^	57.53 (9)	Nd2^xi^—Ca2—O3^xiii^	94.26 (14)
Nd1^i^—Ca1—Si1^vii^	57.53 (11)	Nd2^xi^—Ca2—O3^xiv^	94.26 (14)
Nd1^i^—Ca1—Si1^viii^	57.53 (8)	Nd2^xi^—Ca2—O4	30.00 (3)
Nd1^i^—Ca1—O1	136.5 (2)	Si1—Ca2—Si1^xi^	165.1 (2)
Nd1^i^—Ca1—O1^ix^	136.54 (17)	Si1—Ca2—O2^xii^	67.0 (4)
Nd1^i^—Ca1—O1^x^	136.5 (3)	Si1—Ca2—O3^xiii^	105.11 (18)
Nd1^ii^—Ca1—Ca2^iii^	64.17 (6)	Si1—Ca2—O3^xiv^	105.11 (18)
Nd1^ii^—Ca1—Ca2^iv^	64.17 (5)	Si1—Ca2—O4	83.72 (13)
Nd1^ii^—Ca1—Ca2^v^	64.17 (6)	Si1^xi^—Ca2—O2^xii^	127.8 (4)
Nd1^ii^—Ca1—Si1^vi^	122.47 (9)	Si1^xi^—Ca2—O3^xiii^	78.36 (16)
Nd1^ii^—Ca1—Si1^vii^	122.47 (11)	Si1^xi^—Ca2—O3^xiv^	78.36 (16)
Nd1^ii^—Ca1—Si1^viii^	122.47 (8)	Si1^xi^—Ca2—O4	81.43 (17)
Nd1^ii^—Ca1—O1	43.5 (2)	O2^xii^—Ca2—O3^xiii^	85.50 (19)
Nd1^ii^—Ca1—O1^ix^	43.46 (17)	O2^xii^—Ca2—O3^xiv^	85.50 (19)
Nd1^ii^—Ca1—O1^x^	43.5 (3)	O2^xii^—Ca2—O4	150.7 (4)
Ca2^iii^—Ca1—Ca2^iv^	102.43 (7)	O3^xiii^—Ca2—O3^xiv^	142.0 (3)
Ca2^iii^—Ca1—Ca2^v^	102.43 (7)	O3^xiii^—Ca2—O4	102.89 (19)
Ca2^iii^—Ca1—Si1^vi^	144.87 (10)	O3^xiv^—Ca2—O4	102.89 (19)
Ca2^iii^—Ca1—Si1^vii^	61.42 (11)	Ca2—Nd2—Ca2^iv^	0
Ca2^iii^—Ca1—Si1^viii^	111.25 (16)	Ca2—Nd2—Ca2^xi^	0
Ca2^iii^—Ca1—O1	106.2 (3)	Ca2—Nd2—O2^xii^	0
Ca2^iii^—Ca1—O1^ix^	62.3 (4)	Ca2—Nd2—O3^xiii^	0
Ca2^iii^—Ca1—O1^x^	40.3 (2)	Ca2—Nd2—O3^xiv^	0
Ca2^iv^—Ca1—Ca2^v^	102.42 (7)	Ca2—Nd2—O4	0
Ca2^iv^—Ca1—Si1^vi^	111.25 (11)	Ca2^iv^—Nd2—Ca2^xi^	60.00 (4)
Ca2^iv^—Ca1—Si1^vii^	144.87 (12)	Ca2^iv^—Nd2—O2^xii^	120.7 (4)
Ca2^iv^—Ca1—Si1^viii^	61.42 (5)	Ca2^iv^—Nd2—O3^xiii^	108.2 (2)
Ca2^iv^—Ca1—O1	40.3 (2)	Ca2^iv^—Nd2—O3^xiv^	108.2 (2)
Ca2^iv^—Ca1—O1^ix^	106.2 (2)	Ca2^iv^—Nd2—O4	30.00 (4)
Ca2^iv^—Ca1—O1^x^	62.3 (2)	Ca2^xi^—Nd2—O2^xii^	179.3 (4)
Ca2^v^—Ca1—Si1^vi^	61.42 (6)	Ca2^xi^—Nd2—O3^xiii^	94.26 (14)
Ca2^v^—Ca1—Si1^vii^	111.25 (10)	Ca2^xi^—Nd2—O3^xiv^	94.26 (14)
Ca2^v^—Ca1—Si1^viii^	144.87 (16)	Ca2^xi^—Nd2—O4	30.00 (3)
Ca2^v^—Ca1—O1	62.3 (2)	O2^xii^—Nd2—O3^xiii^	85.50 (19)
Ca2^v^—Ca1—O1^ix^	40.3 (4)	O2^xii^—Nd2—O3^xiv^	85.50 (19)
Ca2^v^—Ca1—O1^x^	106.2 (3)	O2^xii^—Nd2—O4	150.7 (4)
Si1^vi^—Ca1—Si1^vii^	93.88 (18)	O3^xiii^—Nd2—O3^xiv^	142.0 (3)
Si1^vi^—Ca1—Si1^viii^	93.88 (17)	O3^xiii^—Nd2—O4	102.89 (19)
Si1^vi^—Ca1—O1	93.7 (3)	O3^xiv^—Nd2—O4	102.89 (19)
Si1^vi^—Ca1—O1^ix^	97.8 (3)	Ca1^xvii^—Si1—Ca1^xviii^	64.95 (16)
Si1^vi^—Ca1—O1^x^	165.6 (3)	Ca1^xvii^—Si1—Ca2	80.53 (16)
Si1^vii^—Ca1—Si1^viii^	93.88 (15)	Ca1^xvii^—Si1—Ca2^iv^	139.09 (12)
Si1^vii^—Ca1—O1	165.6 (2)	Ca1^xvii^—Si1—O1	137.9 (2)
Si1^vii^—Ca1—O1^ix^	93.7 (3)	Ca1^xvii^—Si1—O2	48.5 (3)
Si1^vii^—Ca1—O1^x^	97.8 (3)	Ca1^xvii^—Si1—O3	111.9 (4)
Si1^viii^—Ca1—O1	97.83 (18)	Ca1^xvii^—Si1—O3^ii^	63.3 (3)
Si1^viii^—Ca1—O1^ix^	165.6 (2)	Ca1^xviii^—Si1—Ca2	80.53 (16)
Si1^viii^—Ca1—O1^x^	93.7 (2)	Ca1^xviii^—Si1—Ca2^iv^	139.09 (12)
O1—Ca1—O1^ix^	73.1 (3)	Ca1^xviii^—Si1—O1	137.9 (2)
O1—Ca1—O1^x^	73.1 (4)	Ca1^xviii^—Si1—O2	48.5 (3)
O1^ix^—Ca1—O1^x^	73.1 (3)	Ca1^xviii^—Si1—O3	63.3 (3)
Ca1—Nd1—Ca1^i^	0	Ca1^xviii^—Si1—O3^ii^	111.9 (4)
Ca1—Nd1—Ca1^ii^	0	Ca2—Si1—Ca2^iv^	74.85 (11)
Ca1—Nd1—Nd1^i^	0	Ca2—Si1—O1	129.6 (4)
Ca1—Nd1—Nd1^ii^	0	Ca2—Si1—O2	117.1 (6)
Ca1—Nd1—O1	0	Ca2—Si1—O3	50.3 (3)
Ca1—Nd1—O1^ix^	0	Ca2—Si1—O3^ii^	50.3 (3)
Ca1—Nd1—O1^x^	0	Ca2^iv^—Si1—O1	54.8 (3)
Ca1^i^—Nd1—Ca1^ii^	180	Ca2^iv^—Si1—O2	168.1 (6)
Ca1^i^—Nd1—Nd1^i^	0	Ca2^iv^—Si1—O3	75.9 (3)
Ca1^i^—Nd1—Nd1^ii^	180	Ca2^iv^—Si1—O3^ii^	75.9 (3)
Ca1^i^—Nd1—O1	136.5 (2)	O1—Si1—O2	113.3 (6)
Ca1^i^—Nd1—O1^ix^	136.54 (17)	O1—Si1—O3	110.2 (4)
Ca1^i^—Nd1—O1^x^	136.5 (3)	O1—Si1—O3^ii^	110.2 (4)
Ca1^ii^—Nd1—Nd1^i^	180	O2—Si1—O3	111.2 (4)
Ca1^ii^—Nd1—Nd1^ii^	0	O2—Si1—O3^ii^	111.2 (4)
Ca1^ii^—Nd1—O1	43.5 (2)	O3—Si1—O3^ii^	99.9 (4)
Ca1^ii^—Nd1—O1^ix^	43.46 (17)	Ca1—O1—Ca1^ii^	93.1 (4)
Ca1^ii^—Nd1—O1^x^	43.5 (3)	Ca1—O1—Nd1	0
Nd1^i^—Nd1—Nd1^ii^	180	Ca1—O1—Nd1^ii^	93.1 (4)
Nd1^i^—Nd1—O1	136.5 (2)	Ca1—O1—Si1	127.6 (3)
Nd1^i^—Nd1—O1^ix^	136.54 (17)	Ca1^ii^—O1—Nd1	93.1 (4)
Nd1^i^—Nd1—O1^x^	136.5 (3)	Ca1^ii^—O1—Nd1^ii^	0
Nd1^ii^—Nd1—O1	43.5 (2)	Ca1^ii^—O1—Si1	127.6 (3)
Nd1^ii^—Nd1—O1^ix^	43.46 (17)	Nd1—O1—Nd1^ii^	93.1 (4)
Nd1^ii^—Nd1—O1^x^	43.5 (3)	Nd1—O1—Si1	127.6 (3)
O1—Nd1—O1^ix^	73.1 (3)	Nd1^ii^—O1—Si1	127.6 (3)
O1—Nd1—O1^x^	73.1 (4)	Ca2^v^—O2—Nd2^v^	0
O1^ix^—Nd1—O1^x^	73.1 (3)	Ca2^v^—O2—Si1	124.1 (7)
Ca1^xv^—Ca2—Ca1^xvi^	51.67 (8)	Nd2^v^—O2—Si1	124.1 (7)
Ca1^xv^—Ca2—Ca2^iv^	150.53 (5)	Ca2^viii^—O3—Nd2^viii^	0
Ca1^xv^—Ca2—Ca2^xi^	103.74 (4)	Ca2^viii^—O3—Si1	140.8 (5)
Ca1^xv^—Ca2—Nd2	0	Nd2^viii^—O3—Si1	140.8 (5)
Ca1^xv^—Ca2—Nd2^iv^	150.53 (5)	Ca2—O4—Ca2^iv^	120.00 (6)
Ca1^xv^—Ca2—Nd2^xi^	103.74 (4)	Ca2—O4—Ca2^xi^	120.00 (7)
Ca1^xv^—Ca2—Si1	134.37 (10)	Ca2—O4—Nd2	0
Ca1^xv^—Ca2—Si1^xi^	57.95 (14)	Ca2—O4—Nd2^iv^	120.00 (6)
Ca1^xv^—Ca2—O2^xii^	75.6 (3)	Ca2—O4—Nd2^xi^	120.00 (7)
Ca1^xv^—Ca2—O3^xiii^	96.8 (2)	Ca2^iv^—O4—Ca2^xi^	120.00 (7)
Ca1^xv^—Ca2—O3^xiv^	45.2 (2)	Ca2^iv^—O4—Nd2	120.00 (6)
Ca1^xv^—Ca2—O4	129.78 (4)	Ca2^iv^—O4—Nd2^iv^	0
Ca1^xvi^—Ca2—Ca2^iv^	150.53 (5)	Ca2^iv^—O4—Nd2^xi^	120.00 (7)
Ca1^xvi^—Ca2—Ca2^xi^	103.74 (4)	Ca2^xi^—O4—Nd2	120.00 (7)
Ca1^xvi^—Ca2—Nd2	0	Ca2^xi^—O4—Nd2^iv^	120.00 (7)
Ca1^xvi^—Ca2—Nd2^iv^	150.53 (5)	Ca2^xi^—O4—Nd2^xi^	0
Ca1^xvi^—Ca2—Nd2^xi^	103.74 (4)	Nd2—O4—Nd2^iv^	120.00 (6)
Ca1^xvi^—Ca2—Si1	134.37 (10)	Nd2—O4—Nd2^xi^	120.00 (7)
Ca1^xvi^—Ca2—Si1^xi^	57.95 (14)	Nd2^iv^—O4—Nd2^xi^	120.00 (7)
Ca1^xvi^—Ca2—O2^xii^	75.6 (3)		

Symmetry codes: (i) *x*, *y*, −*z*−1/2; (ii) *x*, *y*, −*z*+1/2; (iii) *x*, *y*+1, *z*; (iv) −*y*, *x*−*y*, *z*; (v) −*x*+*y*+1, −*x*+1, *z*; (vi) −*x*+1, −*y*+1, *z*−1/2; (vii) *y*, −*x*+*y*+1, *z*−1/2; (viii) *x*−*y*, *x*, *z*−1/2; (ix) −*y*+1, *x*−*y*+1, *z*; (x) −*x*+*y*, −*x*+1, *z*; (xi) −*x*+*y*, −*x*, *z*; (xii) −*y*+1, *x*−*y*, *z*; (xiii) *y*, −*x*+*y*, *z*+1/2; (xiv) *y*, −*x*+*y*, −*z*; (xv) *x*, *y*−1, *z*; (xvi) *x*, *y*−1, −*z*+1/2; (xvii) −*x*+1, −*y*+1, *z*+1/2; (xviii) −*x*+1, −*y*+1, −*z*.

### Calcium samarium silicate oxyapatite (Ca-Sm) Crystal data

**Table:**

Ca_2_Sm_8_(SiO_4_)_6_O_2_	*Z* = 1
*M**_r_* = 1867.3	*D*_x_ = 5.750 Mg m^−^^3^
Hexagonal, *P*6_3_/*m*	Cu *K*α radiation, λ = 1.54188 Å
*a* = 9.46696 (6) Å	*T* = 295 K
*c* = 6.94810 (5) Å	yellow_gray
*V* = 539.28 (1) Å^3^	flat_sheet, 25 × 25 mm

### Calcium samarium silicate oxyapatite (Ca-Sm) Data collection

**Table:**

Bruker D8 Advance diffractometer	Data collection mode: reflection
Radiation source: sealed X-ray tube	Scan method: step
Specimen mounting: packed powder pellet	2θ_min_ = 10°, 2θ_max_ = 70°, 2θ_step_ = 0.009°

### Calcium samarium silicate oxyapatite (Ca-Sm) Refinement

**Table:**

*R*_p_ = 0.04	26 parameters
*R*_wp_ = 0.06	Weighting scheme based on measured s.u.'s
*R*_exp_ = 0.03	(Δ/σ)_max_ = 0.013
*R*_Bragg_ = 0.04	Background function: Chebychev
6994 data points	Preferred orientation correction: spherical harmonic
Profile function: pseudo-Voigt	

### Calcium samarium silicate oxyapatite (Ca-Sm) Special details

**Table:**

Refinement. Beq were fixed as 1Å squared during refinement as they result high errors

### Calcium samarium silicate oxyapatite (Ca-Sm) Fractional atomic coordinates and isotropic or equivalent isotropic displacement parameters (Å^2^)

**Table:**

	*x*	*y*	*z*	*U*_iso_*/*U*_eq_	Occ. (<1)
Ca1	0.333333	0.666667	−0.0007 (7)	0.0127*	0.448
Sm1	0.333333	0.666667	−0.0007 (7)	0.0127*	0.552
Ca2	0.23285 (16)	−0.0082 (3)	0.25	0.0127*	0.035
Sm2	0.23285 (16)	−0.0082 (3)	0.25	0.0127*	0.965
Si1	0.3996 (7)	0.3727 (8)	0.25	0.0127*	
O1	0.3267 (12)	0.4885 (12)	0.25	0.0127*	
O2	0.5929 (13)	0.4699 (12)	0.25	0.0127*	
O3	0.3402 (7)	0.2520 (7)	0.0699 (8)	0.0127*	
O4	0	0	0.25	0.0127*	

### Calcium samarium silicate oxyapatite (Ca-Sm) Atomic displacement parameters (Å^2^)

**Table:**

	*U*^11^	*U*^22^	*U*^33^	*U*^12^	*U*^13^	*U*^23^
?	?	?	?	?	?	?

### Calcium samarium silicate oxyapatite (Ca-Sm) Geometric parameters (Å, º)

**Table:**

Ca1—Ca1^i^	3.464 (7)	Ca2—Ca2^xii^	4.1359 (14)
Ca1—Ca1^ii^	3.484 (7)	Ca2—Ca2^xiii^	4.1359 (14)
Ca1—Sm1	0	Ca2—Ca2^viii^	4.136 (2)
Ca1—Sm1^i^	3.464 (7)	Ca2—Ca2^xiv^	4.136 (2)
Ca1—Sm1^ii^	3.484 (7)	Ca2—Sm2	0
Ca1—Ca2^iii^	4.042 (3)	Ca2—Sm2^iv^	3.887 (5)
Ca1—Ca2^iv^	4.042 (3)	Ca2—Sm2^xi^	3.887 (3)
Ca1—Ca2^v^	4.042 (4)	Ca2—Si1	3.131 (7)
Ca1—Ca2^vi^	4.128 (2)	Ca2—Si1^xi^	3.266 (6)
Ca1—Ca2^vii^	4.128 (4)	Ca2—O2^xv^	2.443 (10)
Ca1—Ca2^viii^	4.128 (3)	Ca2—O3^xiii^	2.384 (6)
Ca1—Si1^vi^	3.236 (7)	Ca2—O3^xvi^	2.384 (6)
Ca1—Si1^vii^	3.236 (9)	Ca2—O4	2.244 (2)
Ca1—Si1^viii^	3.236 (6)	Sm2—Si1	3.131 (7)
Ca1—O1	2.404 (9)	Sm2—O3^xiii^	2.384 (6)
Ca1—O1^ix^	2.404 (8)	Sm2—O3^xvi^	2.384 (6)
Ca1—O1^x^	2.404 (12)	Sm2—O4	2.244 (2)
Sm1—Sm1^i^	3.464 (7)	Si1—O1	1.560 (16)
Sm1—Sm1^ii^	3.484 (7)	Si1—O2	1.585 (12)
Ca2—Ca2^iv^	3.887 (5)	Si1—O3	1.595 (7)
Ca2—Ca2^xi^	3.887 (3)	Si1—O3^ii^	1.595 (7)
			
Ca1^i^—Ca1—Ca1^ii^	180	Ca1^xx^—Ca2—Ca2^xi^	103.30 (5)
Ca1^i^—Ca1—Sm1	0	Ca1^xx^—Ca2—Ca2^xii^	103.49 (9)
Ca1^i^—Ca1—Sm1^i^	0	Ca1^xx^—Ca2—Ca2^xiii^	60.62 (8)
Ca1^i^—Ca1—Sm1^ii^	180	Ca1^xx^—Ca2—Ca2^viii^	146.66 (9)
Ca1^i^—Ca1—Ca2^iii^	115.52 (6)	Ca1^xx^—Ca2—Ca2^xiv^	96.40 (7)
Ca1^i^—Ca1—Ca2^iv^	115.52 (6)	Ca1^xx^—Ca2—Sm2	0
Ca1^i^—Ca1—Ca2^v^	115.52 (7)	Ca1^xx^—Ca2—Sm2^iv^	150.54 (6)
Ca1^i^—Ca1—Ca2^vi^	65.19 (6)	Ca1^xx^—Ca2—Sm2^xi^	103.30 (5)
Ca1^i^—Ca1—Ca2^vii^	65.19 (6)	Ca1^xx^—Ca2—Si1	134.58 (12)
Ca1^i^—Ca1—Ca2^viii^	65.19 (6)	Ca1^xx^—Ca2—Si1^xi^	57.75 (17)
Ca1^i^—Ca1—Si1^vi^	57.64 (10)	Ca1^xx^—Ca2—O2^xv^	74.8 (4)
Ca1^i^—Ca1—Si1^vii^	57.64 (12)	Ca1^xx^—Ca2—O3^xiii^	43.3 (2)
Ca1^i^—Ca1—Si1^viii^	57.64 (10)	Ca1^xx^—Ca2—O3^xvi^	94.3 (3)
Ca1^i^—Ca1—O1	136.4 (2)	Ca1^xx^—Ca2—O4	129.46 (6)
Ca1^i^—Ca1—O1^ix^	136.44 (19)	Ca2^iv^—Ca2—Ca2^xi^	60.00 (5)
Ca1^i^—Ca1—O1^x^	136.4 (3)	Ca2^iv^—Ca2—Ca2^xii^	90.00 (6)
Ca1^ii^—Ca1—Sm1	0	Ca2^iv^—Ca2—Ca2^xiii^	90.00 (6)
Ca1^ii^—Ca1—Sm1^i^	180	Ca2^iv^—Ca2—Ca2^viii^	61.97 (5)
Ca1^ii^—Ca1—Sm1^ii^	0	Ca2^iv^—Ca2—Ca2^xiv^	61.97 (5)
Ca1^ii^—Ca1—Ca2^iii^	64.48 (6)	Ca2^iv^—Ca2—Sm2	0
Ca1^ii^—Ca1—Ca2^iv^	64.48 (6)	Ca2^iv^—Ca2—Sm2^iv^	0
Ca1^ii^—Ca1—Ca2^v^	64.48 (7)	Ca2^iv^—Ca2—Sm2^xi^	60.00 (5)
Ca1^ii^—Ca1—Ca2^vi^	114.81 (6)	Ca2^iv^—Ca2—Si1	54.17 (15)
Ca1^ii^—Ca1—Ca2^vii^	114.81 (6)	Ca2^iv^—Ca2—Si1^xi^	111.0 (2)
Ca1^ii^—Ca1—Ca2^viii^	114.81 (6)	Ca2^iv^—Ca2—O2^xv^	122.2 (4)
Ca1^ii^—Ca1—Si1^vi^	122.36 (10)	Ca2^iv^—Ca2—O3^xiii^	110.1 (2)
Ca1^ii^—Ca1—Si1^vii^	122.36 (12)	Ca2^iv^—Ca2—O3^xvi^	110.1 (2)
Ca1^ii^—Ca1—Si1^viii^	122.36 (10)	Ca2^iv^—Ca2—O4	30.00 (5)
Ca1^ii^—Ca1—O1	43.6 (2)	Ca2^xi^—Ca2—Ca2^xii^	61.97 (4)
Ca1^ii^—Ca1—O1^ix^	43.56 (19)	Ca2^xi^—Ca2—Ca2^xiii^	61.97 (4)
Ca1^ii^—Ca1—O1^x^	43.6 (3)	Ca2^xi^—Ca2—Ca2^viii^	90.00 (5)
Sm1—Ca1—Sm1^i^	0	Ca2^xi^—Ca2—Ca2^xiv^	90.00 (5)
Sm1—Ca1—Sm1^ii^	0	Ca2^xi^—Ca2—Sm2	0
Sm1—Ca1—Ca2^iii^	0	Ca2^xi^—Ca2—Sm2^iv^	60.00 (5)
Sm1—Ca1—Ca2^iv^	0	Ca2^xi^—Ca2—Sm2^xi^	0
Sm1—Ca1—Ca2^v^	0	Ca2^xi^—Ca2—Si1	114.17 (16)
Sm1—Ca1—Ca2^vi^	0	Ca2^xi^—Ca2—Si1^xi^	51.02 (19)
Sm1—Ca1—Ca2^vii^	0	Ca2^xi^—Ca2—O2^xv^	177.8 (4)
Sm1—Ca1—Ca2^viii^	0	Ca2^xi^—Ca2—O3^xiii^	94.39 (14)
Sm1—Ca1—Si1^vi^	0	Ca2^xi^—Ca2—O3^xvi^	94.39 (14)
Sm1—Ca1—Si1^vii^	0	Ca2^xi^—Ca2—O4	30.00 (4)
Sm1—Ca1—Si1^viii^	0	Ca2^xii^—Ca2—Ca2^xiii^	114.28 (5)
Sm1—Ca1—O1	0	Ca2^xii^—Ca2—Ca2^viii^	56.06 (4)
Sm1—Ca1—O1^ix^	0	Ca2^xii^—Ca2—Ca2^xiv^	148.52 (9)
Sm1—Ca1—O1^x^	0	Ca2^xii^—Ca2—Sm2	0
Sm1^i^—Ca1—Sm1^ii^	180	Ca2^xii^—Ca2—Sm2^iv^	90.00 (6)
Sm1^i^—Ca1—Ca2^iii^	115.52 (6)	Ca2^xii^—Ca2—Sm2^xi^	61.97 (4)
Sm1^i^—Ca1—Ca2^iv^	115.52 (6)	Ca2^xii^—Ca2—Si1	116.10 (8)
Sm1^i^—Ca1—Ca2^v^	115.52 (7)	Ca2^xii^—Ca2—Si1^xi^	59.57 (6)
Sm1^i^—Ca1—Ca2^vi^	65.19 (6)	Ca2^xii^—Ca2—O2^xv^	117.35 (14)
Sm1^i^—Ca1—Ca2^vii^	65.19 (6)	Ca2^xii^—Ca2—O3^xiii^	136.31 (19)
Sm1^i^—Ca1—Ca2^viii^	65.19 (6)	Ca2^xii^—Ca2—O3^xvi^	32.53 (15)
Sm1^i^—Ca1—Si1^vi^	57.64 (10)	Ca2^xii^—Ca2—O4	74.26 (5)
Sm1^i^—Ca1—Si1^vii^	57.64 (12)	Ca2^xiii^—Ca2—Ca2^viii^	148.52 (9)
Sm1^i^—Ca1—Si1^viii^	57.64 (10)	Ca2^xiii^—Ca2—Ca2^xiv^	56.06 (4)
Sm1^i^—Ca1—O1	136.4 (2)	Ca2^xiii^—Ca2—Sm2	0
Sm1^i^—Ca1—O1^ix^	136.44 (19)	Ca2^xiii^—Ca2—Sm2^iv^	90.00 (6)
Sm1^i^—Ca1—O1^x^	136.4 (3)	Ca2^xiii^—Ca2—Sm2^xi^	61.97 (4)
Sm1^ii^—Ca1—Ca2^iii^	64.48 (6)	Ca2^xiii^—Ca2—Si1	116.10 (8)
Sm1^ii^—Ca1—Ca2^iv^	64.48 (6)	Ca2^xiii^—Ca2—Si1^xi^	59.57 (6)
Sm1^ii^—Ca1—Ca2^v^	64.48 (7)	Ca2^xiii^—Ca2—O2^xv^	117.35 (14)
Sm1^ii^—Ca1—Ca2^vi^	114.81 (6)	Ca2^xiii^—Ca2—O3^xiii^	32.53 (15)
Sm1^ii^—Ca1—Ca2^vii^	114.81 (6)	Ca2^xiii^—Ca2—O3^xvi^	136.31 (19)
Sm1^ii^—Ca1—Ca2^viii^	114.81 (6)	Ca2^xiii^—Ca2—O4	74.26 (5)
Sm1^ii^—Ca1—Si1^vi^	122.36 (10)	Ca2^viii^—Ca2—Ca2^xiv^	114.28 (8)
Sm1^ii^—Ca1—Si1^vii^	122.36 (12)	Ca2^viii^—Ca2—Sm2	0
Sm1^ii^—Ca1—Si1^viii^	122.36 (10)	Ca2^viii^—Ca2—Sm2^iv^	61.97 (5)
Sm1^ii^—Ca1—O1	43.6 (2)	Ca2^viii^—Ca2—Sm2^xi^	90.00 (5)
Sm1^ii^—Ca1—O1^ix^	43.56 (19)	Ca2^viii^—Ca2—Si1	60.33 (5)
Sm1^ii^—Ca1—O1^x^	43.6 (3)	Ca2^viii^—Ca2—Si1^xi^	114.95 (8)
Ca2^iii^—Ca1—Ca2^iv^	102.80 (9)	Ca2^viii^—Ca2—O2^xv^	91.2 (2)
Ca2^iii^—Ca1—Ca2^v^	102.80 (8)	Ca2^viii^—Ca2—O3^xiii^	167.1 (2)
Ca2^iii^—Ca1—Ca2^vi^	96.04 (4)	Ca2^viii^—Ca2—O3^xvi^	53.8 (2)
Ca2^iii^—Ca1—Ca2^vii^	60.81 (4)	Ca2^viii^—Ca2—O4	74.26 (7)
Ca2^iii^—Ca1—Ca2^viii^	157.66 (5)	Ca2^xiv^—Ca2—Sm2	0
Ca2^iii^—Ca1—Si1^vi^	144.40 (11)	Ca2^xiv^—Ca2—Sm2^iv^	61.97 (5)
Ca2^iii^—Ca1—Si1^vii^	60.89 (12)	Ca2^xiv^—Ca2—Sm2^xi^	90.00 (5)
Ca2^iii^—Ca1—Si1^viii^	111.42 (18)	Ca2^xiv^—Ca2—Si1	60.33 (5)
Ca2^iii^—Ca1—O1	106.8 (3)	Ca2^xiv^—Ca2—Si1^xi^	114.95 (8)
Ca2^iii^—Ca1—O1^ix^	61.7 (4)	Ca2^xiv^—Ca2—O2^xv^	91.2 (2)
Ca2^iii^—Ca1—O1^x^	41.3 (3)	Ca2^xiv^—Ca2—O3^xiii^	53.8 (2)
Ca2^iv^—Ca1—Ca2^v^	102.80 (9)	Ca2^xiv^—Ca2—O3^xvi^	167.1 (2)
Ca2^iv^—Ca1—Ca2^vi^	157.66 (7)	Ca2^xiv^—Ca2—O4	74.26 (7)
Ca2^iv^—Ca1—Ca2^vii^	96.04 (5)	Sm2—Ca2—Sm2^iv^	0
Ca2^iv^—Ca1—Ca2^viii^	60.81 (4)	Sm2—Ca2—Sm2^xi^	0
Ca2^iv^—Ca1—Si1^vi^	111.42 (13)	Sm2—Ca2—Si1	0
Ca2^iv^—Ca1—Si1^vii^	144.40 (13)	Sm2—Ca2—Si1^xi^	0
Ca2^iv^—Ca1—Si1^viii^	60.89 (6)	Sm2—Ca2—O2^xv^	0
Ca2^iv^—Ca1—O1	41.3 (2)	Sm2—Ca2—O3^xiii^	0
Ca2^iv^—Ca1—O1^ix^	106.8 (3)	Sm2—Ca2—O3^xvi^	0
Ca2^iv^—Ca1—O1^x^	61.7 (3)	Sm2—Ca2—O4	0
Ca2^v^—Ca1—Ca2^vi^	60.81 (3)	Sm2^iv^—Ca2—Sm2^xi^	60.00 (5)
Ca2^v^—Ca1—Ca2^vii^	157.66 (4)	Sm2^iv^—Ca2—Si1	54.17 (15)
Ca2^v^—Ca1—Ca2^viii^	96.04 (5)	Sm2^iv^—Ca2—Si1^xi^	111.0 (2)
Ca2^v^—Ca1—Si1^vi^	60.89 (7)	Sm2^iv^—Ca2—O2^xv^	122.2 (4)
Ca2^v^—Ca1—Si1^vii^	111.42 (11)	Sm2^iv^—Ca2—O3^xiii^	110.1 (2)
Ca2^v^—Ca1—Si1^viii^	144.40 (18)	Sm2^iv^—Ca2—O3^xvi^	110.1 (2)
Ca2^v^—Ca1—O1	61.7 (2)	Sm2^iv^—Ca2—O4	30.00 (5)
Ca2^v^—Ca1—O1^ix^	41.3 (4)	Sm2^xi^—Ca2—Si1	114.17 (16)
Ca2^v^—Ca1—O1^x^	106.8 (3)	Sm2^xi^—Ca2—Si1^xi^	51.02 (19)
Ca2^vi^—Ca1—Ca2^vii^	103.65 (8)	Sm2^xi^—Ca2—O2^xv^	177.8 (4)
Ca2^vi^—Ca1—Ca2^viii^	103.65 (9)	Sm2^xi^—Ca2—O3^xiii^	94.39 (14)
Ca2^vi^—Ca1—Si1^vi^	48.48 (12)	Sm2^xi^—Ca2—O3^xvi^	94.39 (14)
Ca2^vi^—Ca1—Si1^vii^	56.62 (14)	Sm2^xi^—Ca2—O4	30.00 (4)
Ca2^vi^—Ca1—Si1^viii^	122.64 (15)	Si1—Ca2—Si1^xi^	165.2 (2)
Ca2^vi^—Ca1—O1	121.4 (3)	Si1—Ca2—O2^xv^	68.0 (4)
Ca2^vi^—Ca1—O1^ix^	71.80 (16)	Si1—Ca2—O3^xiii^	106.9 (2)
Ca2^vi^—Ca1—O1^x^	134.8 (2)	Si1—Ca2—O3^xvi^	106.9 (2)
Ca2^vii^—Ca1—Ca2^viii^	103.65 (9)	Si1—Ca2—O4	84.17 (16)
Ca2^vii^—Ca1—Si1^vi^	122.64 (16)	Si1^xi^—Ca2—O2^xv^	126.8 (5)
Ca2^vii^—Ca1—Si1^vii^	48.48 (13)	Si1^xi^—Ca2—O3^xiii^	76.90 (18)
Ca2^vii^—Ca1—Si1^viii^	56.62 (18)	Si1^xi^—Ca2—O3^xvi^	76.90 (18)
Ca2^vii^—Ca1—O1	134.8 (3)	Si1^xi^—Ca2—O4	81.02 (19)
Ca2^vii^—Ca1—O1^ix^	121.4 (4)	O2^xv^—Ca2—O3^xiii^	84.9 (2)
Ca2^vii^—Ca1—O1^x^	71.8 (3)	O2^xv^—Ca2—O3^xvi^	84.9 (2)
Ca2^viii^—Ca1—Si1^vi^	56.62 (11)	O2^xv^—Ca2—O4	152.2 (4)
Ca2^viii^—Ca1—Si1^vii^	122.64 (16)	O3^xiii^—Ca2—O3^xvi^	137.6 (4)
Ca2^viii^—Ca1—Si1^viii^	48.48 (19)	O3^xiii^—Ca2—O4	104.06 (19)
Ca2^viii^—Ca1—O1	71.8 (2)	O3^xvi^—Ca2—O4	104.06 (19)
Ca2^viii^—Ca1—O1^ix^	134.8 (4)	Ca2—Sm2—Ca2^iv^	0
Ca2^viii^—Ca1—O1^x^	121.4 (2)	Ca2—Sm2—Ca2^xi^	0
Si1^vi^—Ca1—Si1^vii^	94.0 (2)	Ca2—Sm2—Si1	0
Si1^vi^—Ca1—Si1^viii^	94.0 (2)	Ca2—Sm2—O3^xiii^	0
Si1^vi^—Ca1—O1	93.1 (3)	Ca2—Sm2—O3^xvi^	0
Si1^vi^—Ca1—O1^ix^	98.1 (3)	Ca2—Sm2—O4	0
Si1^vi^—Ca1—O1^x^	165.4 (4)	Ca2^iv^—Sm2—Ca2^xi^	60.00 (5)
Si1^vii^—Ca1—Si1^viii^	94.03 (18)	Ca2^iv^—Sm2—Si1	54.17 (15)
Si1^vii^—Ca1—O1	165.4 (3)	Ca2^iv^—Sm2—O3^xiii^	110.1 (2)
Si1^vii^—Ca1—O1^ix^	93.1 (3)	Ca2^iv^—Sm2—O3^xvi^	110.1 (2)
Si1^vii^—Ca1—O1^x^	98.1 (3)	Ca2^iv^—Sm2—O4	30.00 (5)
Si1^viii^—Ca1—O1	98.1 (2)	Ca2^xi^—Sm2—Si1	114.17 (16)
Si1^viii^—Ca1—O1^ix^	165.4 (2)	Ca2^xi^—Sm2—O3^xiii^	94.39 (14)
Si1^viii^—Ca1—O1^x^	93.1 (3)	Ca2^xi^—Sm2—O3^xvi^	94.39 (14)
O1—Ca1—O1^ix^	73.3 (4)	Ca2^xi^—Sm2—O4	30.00 (4)
O1—Ca1—O1^x^	73.3 (4)	Si1—Sm2—O3^xiii^	106.9 (2)
O1^ix^—Ca1—O1^x^	73.3 (4)	Si1—Sm2—O3^xvi^	106.9 (2)
Ca1—Sm1—Ca1^i^	0	Si1—Sm2—O4	84.17 (16)
Ca1—Sm1—Ca1^ii^	0	O3^xiii^—Sm2—O3^xvi^	137.6 (4)
Ca1—Sm1—Sm1^i^	0	O3^xiii^—Sm2—O4	104.06 (19)
Ca1—Sm1—Sm1^ii^	0	O3^xvi^—Sm2—O4	104.06 (19)
Ca1^i^—Sm1—Ca1^ii^	180	Ca1^xviii^—Si1—Ca1^xix^	64.72 (18)
Ca1^i^—Sm1—Sm1^i^	0	Ca1^xviii^—Si1—Ca2	80.82 (19)
Ca1^i^—Sm1—Sm1^ii^	180	Ca1^xviii^—Si1—Ca2^iv^	139.35 (14)
Ca1^ii^—Sm1—Sm1^i^	180	Ca1^xviii^—Si1—Sm2	80.82 (19)
Ca1^ii^—Sm1—Sm1^ii^	0	Ca1^xviii^—Si1—O1	136.6 (3)
Sm1^i^—Sm1—Sm1^ii^	180	Ca1^xviii^—Si1—O2	47.6 (4)
Ca1^xvii^—Ca2—Ca1^xviii^	104.86 (6)	Ca1^xviii^—Si1—O3	110.7 (5)
Ca1^xvii^—Ca2—Ca1^xix^	83.96 (5)	Ca1^xviii^—Si1—O3^ii^	60.9 (3)
Ca1^xvii^—Ca2—Ca1^xx^	51.05 (10)	Ca1^xix^—Si1—Ca2	80.82 (19)
Ca1^xvii^—Ca2—Ca2^iv^	150.54 (6)	Ca1^xix^—Si1—Ca2^iv^	139.35 (14)
Ca1^xvii^—Ca2—Ca2^xi^	103.30 (5)	Ca1^xix^—Si1—Sm2	80.82 (19)
Ca1^xvii^—Ca2—Ca2^xii^	60.62 (8)	Ca1^xix^—Si1—O1	136.6 (3)
Ca1^xvii^—Ca2—Ca2^xiii^	103.49 (9)	Ca1^xix^—Si1—O2	47.6 (4)
Ca1^xvii^—Ca2—Ca2^viii^	96.40 (7)	Ca1^xix^—Si1—O3	60.9 (3)
Ca1^xvii^—Ca2—Ca2^xiv^	146.66 (9)	Ca1^xix^—Si1—O3^ii^	110.7 (5)
Ca1^xvii^—Ca2—Sm2	0	Ca2—Si1—Ca2^iv^	74.81 (13)
Ca1^xvii^—Ca2—Sm2^iv^	150.54 (6)	Ca2—Si1—Sm2	0
Ca1^xvii^—Ca2—Sm2^xi^	103.30 (5)	Ca2—Si1—O1	131.6 (4)
Ca1^xvii^—Ca2—Si1	134.58 (12)	Ca2—Si1—O2	116.1 (6)
Ca1^xvii^—Ca2—Si1^xi^	57.75 (17)	Ca2—Si1—O3	51.8 (3)
Ca1^xvii^—Ca2—O2^xv^	74.8 (4)	Ca2—Si1—O3^ii^	51.8 (3)
Ca1^xvii^—Ca2—O3^xiii^	94.3 (3)	Ca2^iv^—Si1—Sm2	74.81 (13)
Ca1^xvii^—Ca2—O3^xvi^	43.3 (2)	Ca2^iv^—Si1—O1	56.8 (4)
Ca1^xvii^—Ca2—O4	129.46 (6)	Ca2^iv^—Si1—O2	169.1 (6)
Ca1^xviii^—Ca2—Ca1^xix^	49.62 (9)	Ca2^iv^—Si1—O3	78.5 (3)
Ca1^xviii^—Ca2—Ca1^xx^	83.96 (5)	Ca2^iv^—Si1—O3^ii^	78.5 (3)
Ca1^xviii^—Ca2—Ca2^iv^	99.01 (6)	Sm2—Si1—O1	131.6 (4)
Ca1^xviii^—Ca2—Ca2^xi^	148.50 (7)	Sm2—Si1—O2	116.1 (6)
Ca1^xviii^—Ca2—Ca2^xii^	146.89 (7)	Sm2—Si1—O3	51.8 (3)
Ca1^xviii^—Ca2—Ca2^xiii^	97.63 (7)	Sm2—Si1—O3^ii^	51.8 (3)
Ca1^xviii^—Ca2—Ca2^viii^	100.57 (7)	O1—Si1—O2	112.3 (7)
Ca1^xviii^—Ca2—Ca2^xiv^	58.57 (7)	O1—Si1—O3	112.5 (5)
Ca1^xviii^—Ca2—Sm2	0	O1—Si1—O3^ii^	112.5 (5)
Ca1^xviii^—Ca2—Sm2^iv^	99.01 (6)	O2—Si1—O3	107.9 (5)
Ca1^xviii^—Ca2—Sm2^xi^	148.50 (7)	O2—Si1—O3^ii^	107.9 (5)
Ca1^xviii^—Ca2—Si1	50.71 (12)	O3—Si1—O3^ii^	103.3 (5)
Ca1^xviii^—Ca2—Si1^xi^	141.12 (16)	Ca1—O1—Ca1^ii^	92.9 (4)
Ca1^xviii^—Ca2—O2^xv^	32.8 (3)	Ca1—O1—Si1	128.9 (3)
Ca1^xviii^—Ca2—O3^xiii^	69.74 (15)	Ca1^ii^—O1—Si1	128.9 (3)
Ca1^xviii^—Ca2—O3^xvi^	115.85 (15)	Ca2^v^—O2—Si1	124.1 (8)
Ca1^xviii^—Ca2—O4	125.64 (9)	Ca2^viii^—O3—Sm2^viii^	0
Ca1^xix^—Ca2—Ca1^xx^	104.86 (6)	Ca2^viii^—O3—Si1	140.4 (5)
Ca1^xix^—Ca2—Ca2^iv^	99.01 (6)	Sm2^viii^—O3—Si1	140.4 (5)
Ca1^xix^—Ca2—Ca2^xi^	148.50 (7)	Ca2—O4—Ca2^iv^	120.00 (9)
Ca1^xix^—Ca2—Ca2^xii^	97.63 (7)	Ca2—O4—Ca2^xi^	120.00 (10)
Ca1^xix^—Ca2—Ca2^xiii^	146.89 (7)	Ca2—O4—Sm2	0
Ca1^xix^—Ca2—Ca2^viii^	58.57 (7)	Ca2—O4—Sm2^iv^	120.00 (9)
Ca1^xix^—Ca2—Ca2^xiv^	100.57 (7)	Ca2—O4—Sm2^xi^	120.00 (10)
Ca1^xix^—Ca2—Sm2	0	Ca2^iv^—O4—Ca2^xi^	120.00 (10)
Ca1^xix^—Ca2—Sm2^iv^	99.01 (6)	Ca2^iv^—O4—Sm2	120.00 (9)
Ca1^xix^—Ca2—Sm2^xi^	148.50 (7)	Ca2^iv^—O4—Sm2^iv^	0
Ca1^xix^—Ca2—Si1	50.71 (12)	Ca2^iv^—O4—Sm2^xi^	120.00 (10)
Ca1^xix^—Ca2—Si1^xi^	141.12 (16)	Ca2^xi^—O4—Sm2	120.00 (10)
Ca1^xix^—Ca2—O2^xv^	32.8 (3)	Ca2^xi^—O4—Sm2^iv^	120.00 (10)
Ca1^xix^—Ca2—O3^xiii^	115.85 (15)	Ca2^xi^—O4—Sm2^xi^	0
Ca1^xix^—Ca2—O3^xvi^	69.74 (15)	Sm2—O4—Sm2^iv^	120.00 (9)
Ca1^xix^—Ca2—O4	125.64 (9)	Sm2—O4—Sm2^xi^	120.00 (10)
Ca1^xx^—Ca2—Ca2^iv^	150.54 (6)	Sm2^iv^—O4—Sm2^xi^	120.00 (10)

Symmetry codes: (i) *x*, *y*, −*z*−1/2; (ii) *x*, *y*, −*z*+1/2; (iii) *x*, *y*+1, *z*; (iv) −*y*, *x*−*y*, *z*; (v) −*x*+*y*+1, −*x*+1, *z*; (vi) −*x*+1, −*y*+1, *z*−1/2; (vii) *y*, −*x*+*y*+1, *z*−1/2; (viii) *x*−*y*, *x*, *z*−1/2; (ix) −*y*+1, *x*−*y*+1, *z*; (x) −*x*+*y*, −*x*+1, *z*; (xi) −*x*+*y*, −*x*, *z*; (xii) *y*, −*x*+*y*, *z*−1/2; (xiii) *y*, −*x*+*y*, *z*+1/2; (xiv) *x*−*y*, *x*, *z*+1/2; (xv) −*y*+1, *x*−*y*, *z*; (xvi) *y*, −*x*+*y*, −*z*; (xvii) *x*, *y*−1, *z*; (xviii) −*x*+1, −*y*+1, *z*+1/2; (xix) −*x*+1, −*y*+1, −*z*; (xx) *x*, *y*−1, −*z*+1/2.

### Calcium europium silicate oxyapatite (Ca-Eu) Crystal data

**Table:**

Ca_2_Eu_8_(SiO_4_)_6_O_2_	*Z* = 1
*M**_r_* = 1880.1	*D*_x_ = 5.847 Mg m^−^^3^
Hexagonal, *P*6_3_/*m*	Cu *K*α radiation, λ = 1.54188 Å
*a* = 9.44082 (7) Å	*T* = 295 K
*c* = 6.91804 (6) Å	white
*V* = 533.99 (1) Å^3^	flat_sheet, 25 × 25 mm

### Calcium europium silicate oxyapatite (Ca-Eu) Data collection

**Table:**

Bruker D8 Advance diffractometer	Data collection mode: reflection
Radiation source: sealed X-ray tube	Scan method: step
Specimen mounting: packed powder pellet	2θ_min_ = 10°, 2θ_max_ = 70°, 2θ_step_ = 0.009°

### Calcium europium silicate oxyapatite (Ca-Eu) Refinement

**Table:**

*R*_p_ = 0.04	26 parameters
*R*_wp_ = 0.06	Weighting scheme based on measured s.u.'s
*R*_exp_ = 0.03	(Δ/σ)_max_ = 0.032
*R*_Bragg_ = 0.03	Background function: Chebychev
6994 data points	Preferred orientation correction: spherical harmonic
Profile function: pseudo-Voigt	

### Calcium europium silicate oxyapatite (Ca-Eu) Special details

**Table:**

Refinement. Beq were fixed as 1Å squared during refinement as they result high errors

### Calcium europium silicate oxyapatite (Ca-Eu) Fractional atomic coordinates and isotropic or equivalent isotropic displacement parameters (Å^2^)

**Table:**

	*x*	*y*	*z*	*U*_iso_*/*U*_eq_	Occ. (<1)
Ca1	0.333333	0.666667	−0.0001 (8)	0.0127*	0.448
Eu1	0.333333	0.666667	−0.0001 (8)	0.0127*	0.552
Ca2	0.23307 (17)	−0.0073 (3)	0.25	0.0127*	0.035
Eu2	0.23307 (17)	−0.0073 (3)	0.25	0.0127*	0.965
Si1	0.4006 (7)	0.3715 (8)	0.25	0.0127*	
O1	0.3232 (12)	0.4867 (12)	0.25	0.0127*	
O2	0.5961 (13)	0.4708 (12)	0.25	0.0127*	
O3	0.3387 (7)	0.2514 (7)	0.0667 (8)	0.0127*	
O4	0	0	0.25	0.0127*	

### Calcium europium silicate oxyapatite (Ca-Eu) Atomic displacement parameters (Å^2^)

**Table:**

	*U*^11^	*U*^22^	*U*^33^	*U*^12^	*U*^13^	*U*^23^
?	?	?	?	?	?	?

### Calcium europium silicate oxyapatite (Ca-Eu) Geometric parameters (Å, º)

**Table:**

Ca1—Ca1^i^	3.458 (8)	Eu1—Si1^vii^	3.215 (9)
Ca1—Ca1^ii^	3.460 (8)	Eu1—Si1^viii^	3.215 (6)
Ca1—Eu1	0	Eu1—O1	2.393 (9)
Ca1—Eu1^i^	3.458 (8)	Eu1—O1^ix^	2.393 (8)
Ca1—Eu1^ii^	3.460 (8)	Eu1—O1^x^	2.393 (13)
Ca1—Ca2^iii^	4.035 (4)	Ca2—Ca2^iv^	3.872 (5)
Ca1—Ca2^iv^	4.035 (3)	Ca2—Ca2^xi^	3.872 (3)
Ca1—Ca2^v^	4.035 (4)	Ca2—Ca2^xii^	4.1186 (15)
Ca1—Ca2^vi^	4.114 (3)	Ca2—Ca2^xiii^	4.1186 (15)
Ca1—Ca2^vii^	4.114 (4)	Ca2—Ca2^viii^	4.119 (2)
Ca1—Ca2^viii^	4.114 (3)	Ca2—Ca2^xiv^	4.119 (2)
Ca1—Eu2^iii^	4.035 (4)	Ca2—Eu2	0
Ca1—Eu2^iv^	4.035 (3)	Ca2—Eu2^iv^	3.872 (5)
Ca1—Eu2^v^	4.035 (4)	Ca2—Eu2^xi^	3.872 (3)
Ca1—Eu2^vi^	4.114 (3)	Ca2—Eu2^xii^	4.1186 (15)
Ca1—Eu2^vii^	4.114 (4)	Ca2—Eu2^xiii^	4.1186 (15)
Ca1—Eu2^viii^	4.114 (3)	Ca2—Eu2^viii^	4.119 (2)
Ca1—Si1^vi^	3.215 (7)	Ca2—Eu2^xiv^	4.119 (2)
Ca1—Si1^vii^	3.215 (9)	Ca2—Si1	3.104 (7)
Ca1—Si1^viii^	3.215 (6)	Ca2—Si1^xi^	3.275 (6)
Ca1—O1	2.393 (9)	Ca2—O2^xv^	2.426 (10)
Ca1—O1^ix^	2.393 (8)	Ca2—O3^xiii^	2.352 (6)
Ca1—O1^x^	2.393 (13)	Ca2—O3^xvi^	2.352 (6)
Ca1—O2^vi^	2.446 (11)	Ca2—O4	2.236 (2)
Ca1—O2^vii^	2.446 (10)	Eu2—Eu2^iv^	3.872 (5)
Ca1—O2^viii^	2.446 (12)	Eu2—Eu2^xi^	3.872 (3)
Eu1—Eu1^i^	3.458 (8)	Eu2—Eu2^xii^	4.1186 (15)
Eu1—Eu1^ii^	3.460 (8)	Eu2—Eu2^xiii^	4.1186 (15)
Eu1—Ca2^iii^	4.035 (4)	Eu2—Eu2^viii^	4.119 (2)
Eu1—Ca2^iv^	4.035 (3)	Eu2—Eu2^xiv^	4.119 (2)
Eu1—Ca2^v^	4.035 (4)	Eu2—Si1	3.104 (7)
Eu1—Ca2^vi^	4.114 (3)	Eu2—Si1^xi^	3.275 (6)
Eu1—Ca2^vii^	4.114 (4)	Eu2—O2^xv^	2.426 (10)
Eu1—Ca2^viii^	4.114 (3)	Eu2—O3^xiii^	2.352 (6)
Eu1—Eu2^iii^	4.035 (4)	Eu2—O3^xvi^	2.352 (6)
Eu1—Eu2^iv^	4.035 (3)	Eu2—O4	2.236 (2)
Eu1—Eu2^v^	4.035 (4)	Si1—O1	1.585 (16)
Eu1—Eu2^vi^	4.114 (3)	Si1—O2	1.598 (12)
Eu1—Eu2^vii^	4.114 (4)	Si1—O3	1.604 (7)
Eu1—Eu2^viii^	4.114 (3)	Si1—O3^ii^	1.604 (7)
Eu1—Si1^vi^	3.215 (7)		
			
Ca1^i^—Ca1—Ca1^ii^	180	Eu1^xvii^—Ca2—Si1	134.45 (12)
Ca1^i^—Ca1—Eu1	0	Eu1^xvii^—Ca2—Si1^xi^	57.83 (17)
Ca1^i^—Ca1—Eu1^i^	0	Eu1^xvii^—Ca2—O2^xv^	75.2 (4)
Ca1^i^—Ca1—Eu1^ii^	180	Eu1^xvii^—Ca2—O3^xiii^	94.1 (3)
Ca1^i^—Ca1—Ca2^iii^	115.39 (7)	Eu1^xvii^—Ca2—O3^xvi^	43.3 (3)
Ca1^i^—Ca1—Ca2^iv^	115.39 (7)	Eu1^xvii^—Ca2—O4	129.31 (6)
Ca1^i^—Ca1—Ca2^v^	115.39 (7)	Eu1^xviii^—Ca2—Eu1^xix^	49.70 (10)
Ca1^i^—Ca1—Ca2^vi^	65.15 (7)	Eu1^xviii^—Ca2—Eu1^xx^	83.96 (5)
Ca1^i^—Ca1—Ca2^vii^	65.15 (7)	Eu1^xviii^—Ca2—Ca2^iv^	99.26 (6)
Ca1^i^—Ca1—Ca2^viii^	65.15 (7)	Eu1^xviii^—Ca2—Ca2^xi^	148.63 (8)
Ca1^i^—Ca1—Eu2^iii^	115.39 (7)	Eu1^xviii^—Ca2—Ca2^xii^	146.90 (8)
Ca1^i^—Ca1—Eu2^iv^	115.39 (7)	Eu1^xviii^—Ca2—Ca2^xiii^	97.57 (7)
Ca1^i^—Ca1—Eu2^v^	115.39 (7)	Eu1^xviii^—Ca2—Ca2^viii^	100.73 (8)
Ca1^i^—Ca1—Eu2^vi^	65.15 (7)	Eu1^xviii^—Ca2—Ca2^xiv^	58.69 (7)
Ca1^i^—Ca1—Eu2^vii^	65.15 (7)	Eu1^xviii^—Ca2—Eu2	0
Ca1^i^—Ca1—Eu2^viii^	65.15 (7)	Eu1^xviii^—Ca2—Eu2^iv^	99.26 (6)
Ca1^i^—Ca1—Si1^vi^	57.47 (11)	Eu1^xviii^—Ca2—Eu2^xi^	148.63 (8)
Ca1^i^—Ca1—Si1^vii^	57.47 (13)	Eu1^xviii^—Ca2—Eu2^xii^	146.90 (8)
Ca1^i^—Ca1—Si1^viii^	57.47 (10)	Eu1^xviii^—Ca2—Eu2^xiii^	97.57 (7)
Ca1^i^—Ca1—O1	136.3 (2)	Eu1^xviii^—Ca2—Eu2^viii^	100.73 (8)
Ca1^i^—Ca1—O1^ix^	136.30 (19)	Eu1^xviii^—Ca2—Eu2^xiv^	58.69 (7)
Ca1^i^—Ca1—O1^x^	136.3 (3)	Eu1^xviii^—Ca2—Si1	50.56 (12)
Ca1^i^—Ca1—O2^vi^	45.0 (3)	Eu1^xviii^—Ca2—Si1^xi^	141.16 (16)
Ca1^i^—Ca1—O2^vii^	45.0 (2)	Eu1^xviii^—Ca2—O2^xv^	32.5 (3)
Ca1^i^—Ca1—O2^viii^	45.0 (3)	Eu1^xviii^—Ca2—O3^xiii^	69.84 (15)
Ca1^ii^—Ca1—Eu1	0	Eu1^xviii^—Ca2—O3^xvi^	116.00 (15)
Ca1^ii^—Ca1—Eu1^i^	180	Eu1^xviii^—Ca2—O4	125.87 (9)
Ca1^ii^—Ca1—Eu1^ii^	0	Eu1^xix^—Ca2—Eu1^xx^	104.79 (6)
Ca1^ii^—Ca1—Ca2^iii^	64.61 (7)	Eu1^xix^—Ca2—Ca2^iv^	99.26 (6)
Ca1^ii^—Ca1—Ca2^iv^	64.61 (7)	Eu1^xix^—Ca2—Ca2^xi^	148.63 (8)
Ca1^ii^—Ca1—Ca2^v^	64.61 (7)	Eu1^xix^—Ca2—Ca2^xii^	97.57 (7)
Ca1^ii^—Ca1—Ca2^vi^	114.85 (7)	Eu1^xix^—Ca2—Ca2^xiii^	146.90 (8)
Ca1^ii^—Ca1—Ca2^vii^	114.85 (7)	Eu1^xix^—Ca2—Ca2^viii^	58.69 (7)
Ca1^ii^—Ca1—Ca2^viii^	114.85 (7)	Eu1^xix^—Ca2—Ca2^xiv^	100.73 (8)
Ca1^ii^—Ca1—Eu2^iii^	64.61 (7)	Eu1^xix^—Ca2—Eu2	0
Ca1^ii^—Ca1—Eu2^iv^	64.61 (7)	Eu1^xix^—Ca2—Eu2^iv^	99.26 (6)
Ca1^ii^—Ca1—Eu2^v^	64.61 (7)	Eu1^xix^—Ca2—Eu2^xi^	148.63 (8)
Ca1^ii^—Ca1—Eu2^vi^	114.85 (7)	Eu1^xix^—Ca2—Eu2^xii^	97.57 (7)
Ca1^ii^—Ca1—Eu2^vii^	114.85 (7)	Eu1^xix^—Ca2—Eu2^xiii^	146.90 (8)
Ca1^ii^—Ca1—Eu2^viii^	114.85 (7)	Eu1^xix^—Ca2—Eu2^viii^	58.69 (7)
Ca1^ii^—Ca1—Si1^vi^	122.53 (11)	Eu1^xix^—Ca2—Eu2^xiv^	100.73 (8)
Ca1^ii^—Ca1—Si1^vii^	122.53 (13)	Eu1^xix^—Ca2—Si1	50.56 (12)
Ca1^ii^—Ca1—Si1^viii^	122.53 (10)	Eu1^xix^—Ca2—Si1^xi^	141.16 (16)
Ca1^ii^—Ca1—O1	43.7 (2)	Eu1^xix^—Ca2—O2^xv^	32.5 (3)
Ca1^ii^—Ca1—O1^ix^	43.70 (19)	Eu1^xix^—Ca2—O3^xiii^	116.00 (15)
Ca1^ii^—Ca1—O1^x^	43.7 (3)	Eu1^xix^—Ca2—O3^xvi^	69.84 (15)
Ca1^ii^—Ca1—O2^vi^	135.0 (3)	Eu1^xix^—Ca2—O4	125.87 (9)
Ca1^ii^—Ca1—O2^vii^	135.0 (2)	Eu1^xx^—Ca2—Ca2^iv^	150.53 (7)
Ca1^ii^—Ca1—O2^viii^	135.0 (3)	Eu1^xx^—Ca2—Ca2^xi^	103.10 (5)
Eu1—Ca1—Eu1^i^	0	Eu1^xx^—Ca2—Ca2^xii^	103.26 (10)
Eu1—Ca1—Eu1^ii^	0	Eu1^xx^—Ca2—Ca2^xiii^	60.59 (8)
Eu1—Ca1—Ca2^iii^	0	Eu1^xx^—Ca2—Ca2^viii^	146.60 (9)
Eu1—Ca1—Ca2^iv^	0	Eu1^xx^—Ca2—Ca2^xiv^	96.58 (8)
Eu1—Ca1—Ca2^v^	0	Eu1^xx^—Ca2—Eu2	0
Eu1—Ca1—Ca2^vi^	0	Eu1^xx^—Ca2—Eu2^iv^	150.53 (7)
Eu1—Ca1—Ca2^vii^	0	Eu1^xx^—Ca2—Eu2^xi^	103.10 (5)
Eu1—Ca1—Ca2^viii^	0	Eu1^xx^—Ca2—Eu2^xii^	103.26 (10)
Eu1—Ca1—Eu2^iii^	0	Eu1^xx^—Ca2—Eu2^xiii^	60.59 (8)
Eu1—Ca1—Eu2^iv^	0	Eu1^xx^—Ca2—Eu2^viii^	146.60 (9)
Eu1—Ca1—Eu2^v^	0	Eu1^xx^—Ca2—Eu2^xiv^	96.58 (8)
Eu1—Ca1—Eu2^vi^	0	Eu1^xx^—Ca2—Si1	134.45 (12)
Eu1—Ca1—Eu2^vii^	0	Eu1^xx^—Ca2—Si1^xi^	57.83 (17)
Eu1—Ca1—Eu2^viii^	0	Eu1^xx^—Ca2—O2^xv^	75.2 (4)
Eu1—Ca1—Si1^vi^	0	Eu1^xx^—Ca2—O3^xiii^	43.3 (3)
Eu1—Ca1—Si1^vii^	0	Eu1^xx^—Ca2—O3^xvi^	94.1 (3)
Eu1—Ca1—Si1^viii^	0	Eu1^xx^—Ca2—O4	129.31 (6)
Eu1—Ca1—O1	0	Ca2^iv^—Ca2—Ca2^xi^	60.00 (6)
Eu1—Ca1—O1^ix^	0	Ca2^iv^—Ca2—Ca2^xii^	90.00 (7)
Eu1—Ca1—O1^x^	0	Ca2^iv^—Ca2—Ca2^xiii^	90.00 (7)
Eu1—Ca1—O2^vi^	0	Ca2^iv^—Ca2—Ca2^viii^	61.96 (6)
Eu1—Ca1—O2^vii^	0	Ca2^iv^—Ca2—Ca2^xiv^	61.96 (6)
Eu1—Ca1—O2^viii^	0	Ca2^iv^—Ca2—Eu2	0
Eu1^i^—Ca1—Eu1^ii^	180	Ca2^iv^—Ca2—Eu2^iv^	0
Eu1^i^—Ca1—Ca2^iii^	115.39 (7)	Ca2^iv^—Ca2—Eu2^xi^	60.00 (6)
Eu1^i^—Ca1—Ca2^iv^	115.39 (7)	Ca2^iv^—Ca2—Eu2^xii^	90.00 (7)
Eu1^i^—Ca1—Ca2^v^	115.39 (7)	Ca2^iv^—Ca2—Eu2^xiii^	90.00 (7)
Eu1^i^—Ca1—Ca2^vi^	65.15 (7)	Ca2^iv^—Ca2—Eu2^viii^	61.96 (6)
Eu1^i^—Ca1—Ca2^vii^	65.15 (7)	Ca2^iv^—Ca2—Eu2^xiv^	61.96 (6)
Eu1^i^—Ca1—Ca2^viii^	65.15 (7)	Ca2^iv^—Ca2—Si1	54.66 (15)
Eu1^i^—Ca1—Eu2^iii^	115.39 (7)	Ca2^iv^—Ca2—Si1^xi^	110.6 (2)
Eu1^i^—Ca1—Eu2^iv^	115.39 (7)	Ca2^iv^—Ca2—O2^xv^	121.9 (4)
Eu1^i^—Ca1—Eu2^v^	115.39 (7)	Ca2^iv^—Ca2—O3^xiii^	110.2 (2)
Eu1^i^—Ca1—Eu2^vi^	65.15 (7)	Ca2^iv^—Ca2—O3^xvi^	110.2 (2)
Eu1^i^—Ca1—Eu2^vii^	65.15 (7)	Ca2^iv^—Ca2—O4	30.00 (5)
Eu1^i^—Ca1—Eu2^viii^	65.15 (7)	Ca2^xi^—Ca2—Ca2^xii^	61.96 (4)
Eu1^i^—Ca1—Si1^vi^	57.47 (11)	Ca2^xi^—Ca2—Ca2^xiii^	61.96 (4)
Eu1^i^—Ca1—Si1^vii^	57.47 (13)	Ca2^xi^—Ca2—Ca2^viii^	90.00 (6)
Eu1^i^—Ca1—Si1^viii^	57.47 (10)	Ca2^xi^—Ca2—Ca2^xiv^	90.00 (6)
Eu1^i^—Ca1—O1	136.3 (2)	Ca2^xi^—Ca2—Eu2	0
Eu1^i^—Ca1—O1^ix^	136.30 (19)	Ca2^xi^—Ca2—Eu2^iv^	60.00 (6)
Eu1^i^—Ca1—O1^x^	136.3 (3)	Ca2^xi^—Ca2—Eu2^xi^	0
Eu1^i^—Ca1—O2^vi^	45.0 (3)	Ca2^xi^—Ca2—Eu2^xii^	61.96 (4)
Eu1^i^—Ca1—O2^vii^	45.0 (2)	Ca2^xi^—Ca2—Eu2^xiii^	61.96 (4)
Eu1^i^—Ca1—O2^viii^	45.0 (3)	Ca2^xi^—Ca2—Eu2^viii^	90.00 (6)
Eu1^ii^—Ca1—Ca2^iii^	64.61 (7)	Ca2^xi^—Ca2—Eu2^xiv^	90.00 (6)
Eu1^ii^—Ca1—Ca2^iv^	64.61 (7)	Ca2^xi^—Ca2—Si1	114.66 (16)
Eu1^ii^—Ca1—Ca2^v^	64.61 (7)	Ca2^xi^—Ca2—Si1^xi^	50.64 (19)
Eu1^ii^—Ca1—Ca2^vi^	114.85 (7)	Ca2^xi^—Ca2—O2^xv^	178.1 (4)
Eu1^ii^—Ca1—Ca2^vii^	114.85 (7)	Ca2^xi^—Ca2—O3^xiii^	94.20 (14)
Eu1^ii^—Ca1—Ca2^viii^	114.85 (7)	Ca2^xi^—Ca2—O3^xvi^	94.20 (14)
Eu1^ii^—Ca1—Eu2^iii^	64.61 (7)	Ca2^xi^—Ca2—O4	30.00 (4)
Eu1^ii^—Ca1—Eu2^iv^	64.61 (7)	Ca2^xii^—Ca2—Ca2^xiii^	114.25 (5)
Eu1^ii^—Ca1—Eu2^v^	64.61 (7)	Ca2^xii^—Ca2—Ca2^viii^	56.08 (4)
Eu1^ii^—Ca1—Eu2^vi^	114.85 (7)	Ca2^xii^—Ca2—Ca2^xiv^	148.50 (9)
Eu1^ii^—Ca1—Eu2^vii^	114.85 (7)	Ca2^xii^—Ca2—Eu2	0
Eu1^ii^—Ca1—Eu2^viii^	114.85 (7)	Ca2^xii^—Ca2—Eu2^iv^	90.00 (7)
Eu1^ii^—Ca1—Si1^vi^	122.53 (11)	Ca2^xii^—Ca2—Eu2^xi^	61.96 (4)
Eu1^ii^—Ca1—Si1^vii^	122.53 (13)	Ca2^xii^—Ca2—Eu2^xii^	0
Eu1^ii^—Ca1—Si1^viii^	122.53 (10)	Ca2^xii^—Ca2—Eu2^xiii^	114.25 (5)
Eu1^ii^—Ca1—O1	43.7 (2)	Ca2^xii^—Ca2—Eu2^viii^	56.08 (4)
Eu1^ii^—Ca1—O1^ix^	43.70 (19)	Ca2^xii^—Ca2—Eu2^xiv^	148.50 (9)
Eu1^ii^—Ca1—O1^x^	43.7 (3)	Ca2^xii^—Ca2—Si1	116.28 (8)
Eu1^ii^—Ca1—O2^vi^	135.0 (3)	Ca2^xii^—Ca2—Si1^xi^	59.47 (6)
Eu1^ii^—Ca1—O2^vii^	135.0 (2)	Ca2^xii^—Ca2—O2^xv^	117.43 (14)
Eu1^ii^—Ca1—O2^viii^	135.0 (3)	Ca2^xii^—Ca2—O3^xiii^	136.1 (2)
Ca2^iii^—Ca1—Ca2^iv^	102.95 (10)	Ca2^xii^—Ca2—O3^xvi^	32.37 (15)
Ca2^iii^—Ca1—Ca2^v^	102.95 (9)	Ca2^xii^—Ca2—O4	74.25 (5)
Ca2^iii^—Ca1—Ca2^vi^	96.04 (4)	Ca2^xiii^—Ca2—Ca2^viii^	148.50 (9)
Ca2^iii^—Ca1—Ca2^vii^	60.71 (4)	Ca2^xiii^—Ca2—Ca2^xiv^	56.08 (4)
Ca2^iii^—Ca1—Ca2^viii^	157.62 (5)	Ca2^xiii^—Ca2—Eu2	0
Ca2^iii^—Ca1—Eu2^iii^	0	Ca2^xiii^—Ca2—Eu2^iv^	90.00 (7)
Ca2^iii^—Ca1—Eu2^iv^	102.95 (10)	Ca2^xiii^—Ca2—Eu2^xi^	61.96 (4)
Ca2^iii^—Ca1—Eu2^v^	102.95 (9)	Ca2^xiii^—Ca2—Eu2^xii^	114.25 (5)
Ca2^iii^—Ca1—Eu2^vi^	96.04 (4)	Ca2^xiii^—Ca2—Eu2^xiii^	0
Ca2^iii^—Ca1—Eu2^vii^	60.71 (4)	Ca2^xiii^—Ca2—Eu2^viii^	148.50 (9)
Ca2^iii^—Ca1—Eu2^viii^	157.62 (5)	Ca2^xiii^—Ca2—Eu2^xiv^	56.08 (4)
Ca2^iii^—Ca1—Si1^vi^	144.17 (11)	Ca2^xiii^—Ca2—Si1	116.28 (8)
Ca2^iii^—Ca1—Si1^vii^	60.87 (12)	Ca2^xiii^—Ca2—Si1^xi^	59.47 (6)
Ca2^iii^—Ca1—Si1^viii^	111.59 (18)	Ca2^xiii^—Ca2—O2^xv^	117.43 (14)
Ca2^iii^—Ca1—O1	106.9 (3)	Ca2^xiii^—Ca2—O3^xiii^	32.37 (15)
Ca2^iii^—Ca1—O1^ix^	62.5 (4)	Ca2^xiii^—Ca2—O3^xvi^	136.1 (2)
Ca2^iii^—Ca1—O1^x^	40.7 (3)	Ca2^xiii^—Ca2—O4	74.25 (5)
Ca2^iii^—Ca1—O2^vi^	159.7 (3)	Ca2^viii^—Ca2—Ca2^xiv^	114.25 (8)
Ca2^iii^—Ca1—O2^vii^	85.6 (4)	Ca2^viii^—Ca2—Eu2	0
Ca2^iii^—Ca1—O2^viii^	92.8 (4)	Ca2^viii^—Ca2—Eu2^iv^	61.96 (6)
Ca2^iv^—Ca1—Ca2^v^	102.95 (9)	Ca2^viii^—Ca2—Eu2^xi^	90.00 (6)
Ca2^iv^—Ca1—Ca2^vi^	157.62 (7)	Ca2^viii^—Ca2—Eu2^xii^	56.08 (4)
Ca2^iv^—Ca1—Ca2^vii^	96.04 (5)	Ca2^viii^—Ca2—Eu2^xiii^	148.50 (9)
Ca2^iv^—Ca1—Ca2^viii^	60.71 (4)	Ca2^viii^—Ca2—Eu2^viii^	0
Ca2^iv^—Ca1—Eu2^iii^	102.95 (10)	Ca2^viii^—Ca2—Eu2^xiv^	114.25 (8)
Ca2^iv^—Ca1—Eu2^iv^	0	Ca2^viii^—Ca2—Si1	60.44 (5)
Ca2^iv^—Ca1—Eu2^v^	102.95 (9)	Ca2^viii^—Ca2—Si1^xi^	114.82 (8)
Ca2^iv^—Ca1—Eu2^vi^	157.62 (7)	Ca2^viii^—Ca2—O2^xv^	91.0 (2)
Ca2^iv^—Ca1—Eu2^vii^	96.04 (5)	Ca2^viii^—Ca2—O3^xiii^	167.3 (2)
Ca2^iv^—Ca1—Eu2^viii^	60.71 (4)	Ca2^viii^—Ca2—O3^xvi^	53.9 (2)
Ca2^iv^—Ca1—Si1^vi^	111.59 (13)	Ca2^viii^—Ca2—O4	74.25 (7)
Ca2^iv^—Ca1—Si1^vii^	144.17 (14)	Ca2^xiv^—Ca2—Eu2	0
Ca2^iv^—Ca1—Si1^viii^	60.87 (6)	Ca2^xiv^—Ca2—Eu2^iv^	61.96 (6)
Ca2^iv^—Ca1—O1	40.7 (2)	Ca2^xiv^—Ca2—Eu2^xi^	90.00 (6)
Ca2^iv^—Ca1—O1^ix^	106.9 (3)	Ca2^xiv^—Ca2—Eu2^xii^	148.50 (9)
Ca2^iv^—Ca1—O1^x^	62.5 (3)	Ca2^xiv^—Ca2—Eu2^xiii^	56.08 (4)
Ca2^iv^—Ca1—O2^vi^	92.76 (19)	Ca2^xiv^—Ca2—Eu2^viii^	114.25 (8)
Ca2^iv^—Ca1—O2^vii^	159.7 (2)	Ca2^xiv^—Ca2—Eu2^xiv^	0
Ca2^iv^—Ca1—O2^viii^	85.6 (2)	Ca2^xiv^—Ca2—Si1	60.44 (5)
Ca2^v^—Ca1—Ca2^vi^	60.71 (3)	Ca2^xiv^—Ca2—Si1^xi^	114.82 (8)
Ca2^v^—Ca1—Ca2^vii^	157.62 (4)	Ca2^xiv^—Ca2—O2^xv^	91.0 (2)
Ca2^v^—Ca1—Ca2^viii^	96.04 (5)	Ca2^xiv^—Ca2—O3^xiii^	53.9 (2)
Ca2^v^—Ca1—Eu2^iii^	102.95 (9)	Ca2^xiv^—Ca2—O3^xvi^	167.3 (2)
Ca2^v^—Ca1—Eu2^iv^	102.95 (9)	Ca2^xiv^—Ca2—O4	74.25 (7)
Ca2^v^—Ca1—Eu2^v^	0	Eu2—Ca2—Eu2^iv^	0
Ca2^v^—Ca1—Eu2^vi^	60.71 (3)	Eu2—Ca2—Eu2^xi^	0
Ca2^v^—Ca1—Eu2^vii^	157.62 (4)	Eu2—Ca2—Eu2^xii^	0
Ca2^v^—Ca1—Eu2^viii^	96.04 (5)	Eu2—Ca2—Eu2^xiii^	0
Ca2^v^—Ca1—Si1^vi^	60.87 (7)	Eu2—Ca2—Eu2^viii^	0
Ca2^v^—Ca1—Si1^vii^	111.59 (11)	Eu2—Ca2—Eu2^xiv^	0
Ca2^v^—Ca1—Si1^viii^	144.17 (18)	Eu2—Ca2—Si1	0
Ca2^v^—Ca1—O1	62.5 (2)	Eu2—Ca2—Si1^xi^	0
Ca2^v^—Ca1—O1^ix^	40.7 (4)	Eu2—Ca2—O2^xv^	0
Ca2^v^—Ca1—O1^x^	106.9 (3)	Eu2—Ca2—O3^xiii^	0
Ca2^v^—Ca1—O2^vi^	85.6 (2)	Eu2—Ca2—O3^xvi^	0
Ca2^v^—Ca1—O2^vii^	92.8 (3)	Eu2—Ca2—O4	0
Ca2^v^—Ca1—O2^viii^	159.7 (3)	Eu2^iv^—Ca2—Eu2^xi^	60.00 (6)
Ca2^vi^—Ca1—Ca2^vii^	103.60 (9)	Eu2^iv^—Ca2—Eu2^xii^	90.00 (7)
Ca2^vi^—Ca1—Ca2^viii^	103.60 (9)	Eu2^iv^—Ca2—Eu2^xiii^	90.00 (7)
Ca2^vi^—Ca1—Eu2^iii^	96.04 (4)	Eu2^iv^—Ca2—Eu2^viii^	61.96 (6)
Ca2^vi^—Ca1—Eu2^iv^	157.62 (7)	Eu2^iv^—Ca2—Eu2^xiv^	61.96 (6)
Ca2^vi^—Ca1—Eu2^v^	60.71 (3)	Eu2^iv^—Ca2—Si1	54.66 (15)
Ca2^vi^—Ca1—Eu2^vi^	0	Eu2^iv^—Ca2—Si1^xi^	110.6 (2)
Ca2^vi^—Ca1—Eu2^vii^	103.60 (9)	Eu2^iv^—Ca2—O2^xv^	121.9 (4)
Ca2^vi^—Ca1—Eu2^viii^	103.60 (9)	Eu2^iv^—Ca2—O3^xiii^	110.2 (2)
Ca2^vi^—Ca1—Si1^vi^	48.22 (12)	Eu2^iv^—Ca2—O3^xvi^	110.2 (2)
Ca2^vi^—Ca1—Si1^vii^	56.79 (14)	Eu2^iv^—Ca2—O4	30.00 (5)
Ca2^vi^—Ca1—Si1^viii^	122.41 (17)	Eu2^xi^—Ca2—Eu2^xii^	61.96 (4)
Ca2^vi^—Ca1—O1	122.0 (3)	Eu2^xi^—Ca2—Eu2^xiii^	61.96 (4)
Ca2^vi^—Ca1—O1^ix^	71.61 (16)	Eu2^xi^—Ca2—Eu2^viii^	90.00 (6)
Ca2^vi^—Ca1—O1^x^	134.2 (2)	Eu2^xi^—Ca2—Eu2^xiv^	90.00 (6)
Ca2^vi^—Ca1—O2^vi^	71.9 (2)	Eu2^xi^—Ca2—Si1	114.66 (16)
Ca2^vi^—Ca1—O2^vii^	32.2 (3)	Eu2^xi^—Ca2—Si1^xi^	50.64 (19)
Ca2^vi^—Ca1—O2^viii^	105.4 (2)	Eu2^xi^—Ca2—O2^xv^	178.1 (4)
Ca2^vii^—Ca1—Ca2^viii^	103.60 (10)	Eu2^xi^—Ca2—O3^xiii^	94.20 (14)
Ca2^vii^—Ca1—Eu2^iii^	60.71 (4)	Eu2^xi^—Ca2—O3^xvi^	94.20 (14)
Ca2^vii^—Ca1—Eu2^iv^	96.04 (5)	Eu2^xi^—Ca2—O4	30.00 (4)
Ca2^vii^—Ca1—Eu2^v^	157.62 (4)	Eu2^xii^—Ca2—Eu2^xiii^	114.25 (5)
Ca2^vii^—Ca1—Eu2^vi^	103.60 (9)	Eu2^xii^—Ca2—Eu2^viii^	56.08 (4)
Ca2^vii^—Ca1—Eu2^vii^	0	Eu2^xii^—Ca2—Eu2^xiv^	148.50 (9)
Ca2^vii^—Ca1—Eu2^viii^	103.60 (10)	Eu2^xii^—Ca2—Si1	116.28 (8)
Ca2^vii^—Ca1—Si1^vi^	122.41 (17)	Eu2^xii^—Ca2—Si1^xi^	59.47 (6)
Ca2^vii^—Ca1—Si1^vii^	48.22 (13)	Eu2^xii^—Ca2—O2^xv^	117.43 (14)
Ca2^vii^—Ca1—Si1^viii^	56.79 (18)	Eu2^xii^—Ca2—O3^xiii^	136.1 (2)
Ca2^vii^—Ca1—O1	134.2 (3)	Eu2^xii^—Ca2—O3^xvi^	32.37 (15)
Ca2^vii^—Ca1—O1^ix^	122.0 (4)	Eu2^xii^—Ca2—O4	74.25 (5)
Ca2^vii^—Ca1—O1^x^	71.6 (3)	Eu2^xiii^—Ca2—Eu2^viii^	148.50 (9)
Ca2^vii^—Ca1—O2^vi^	105.4 (3)	Eu2^xiii^—Ca2—Eu2^xiv^	56.08 (4)
Ca2^vii^—Ca1—O2^vii^	71.9 (3)	Eu2^xiii^—Ca2—Si1	116.28 (8)
Ca2^vii^—Ca1—O2^viii^	32.2 (4)	Eu2^xiii^—Ca2—Si1^xi^	59.47 (6)
Ca2^viii^—Ca1—Eu2^iii^	157.62 (5)	Eu2^xiii^—Ca2—O2^xv^	117.43 (14)
Ca2^viii^—Ca1—Eu2^iv^	60.71 (4)	Eu2^xiii^—Ca2—O3^xiii^	32.37 (15)
Ca2^viii^—Ca1—Eu2^v^	96.04 (5)	Eu2^xiii^—Ca2—O3^xvi^	136.1 (2)
Ca2^viii^—Ca1—Eu2^vi^	103.60 (9)	Eu2^xiii^—Ca2—O4	74.25 (5)
Ca2^viii^—Ca1—Eu2^vii^	103.60 (10)	Eu2^viii^—Ca2—Eu2^xiv^	114.25 (8)
Ca2^viii^—Ca1—Eu2^viii^	0	Eu2^viii^—Ca2—Si1	60.44 (5)
Ca2^viii^—Ca1—Si1^vi^	56.79 (12)	Eu2^viii^—Ca2—Si1^xi^	114.82 (8)
Ca2^viii^—Ca1—Si1^vii^	122.41 (18)	Eu2^viii^—Ca2—O2^xv^	91.0 (2)
Ca2^viii^—Ca1—Si1^viii^	48.22 (19)	Eu2^viii^—Ca2—O3^xiii^	167.3 (2)
Ca2^viii^—Ca1—O1	71.6 (2)	Eu2^viii^—Ca2—O3^xvi^	53.9 (2)
Ca2^viii^—Ca1—O1^ix^	134.2 (4)	Eu2^viii^—Ca2—O4	74.25 (7)
Ca2^viii^—Ca1—O1^x^	122.0 (2)	Eu2^xiv^—Ca2—Si1	60.44 (5)
Ca2^viii^—Ca1—O2^vi^	32.23 (19)	Eu2^xiv^—Ca2—Si1^xi^	114.82 (8)
Ca2^viii^—Ca1—O2^vii^	105.4 (3)	Eu2^xiv^—Ca2—O2^xv^	91.0 (2)
Ca2^viii^—Ca1—O2^viii^	71.9 (4)	Eu2^xiv^—Ca2—O3^xiii^	53.9 (2)
Eu2^iii^—Ca1—Eu2^iv^	102.95 (10)	Eu2^xiv^—Ca2—O3^xvi^	167.3 (2)
Eu2^iii^—Ca1—Eu2^v^	102.95 (9)	Eu2^xiv^—Ca2—O4	74.25 (7)
Eu2^iii^—Ca1—Eu2^vi^	96.04 (4)	Si1—Ca2—Si1^xi^	165.3 (2)
Eu2^iii^—Ca1—Eu2^vii^	60.71 (4)	Si1—Ca2—O2^xv^	67.3 (4)
Eu2^iii^—Ca1—Eu2^viii^	157.62 (5)	Si1—Ca2—O3^xiii^	107.0 (2)
Eu2^iii^—Ca1—Si1^vi^	144.17 (11)	Si1—Ca2—O3^xvi^	107.0 (2)
Eu2^iii^—Ca1—Si1^vii^	60.87 (12)	Si1—Ca2—O4	84.66 (16)
Eu2^iii^—Ca1—Si1^viii^	111.59 (18)	Si1^xi^—Ca2—O2^xv^	127.4 (5)
Eu2^iii^—Ca1—O1	106.9 (3)	Si1^xi^—Ca2—O3^xiii^	76.77 (18)
Eu2^iii^—Ca1—O1^ix^	62.5 (4)	Si1^xi^—Ca2—O3^xvi^	76.77 (18)
Eu2^iii^—Ca1—O1^x^	40.7 (3)	Si1^xi^—Ca2—O4	80.64 (19)
Eu2^iii^—Ca1—O2^vi^	159.7 (3)	O2^xv^—Ca2—O3^xiii^	85.1 (2)
Eu2^iii^—Ca1—O2^vii^	85.6 (4)	O2^xv^—Ca2—O3^xvi^	85.1 (2)
Eu2^iii^—Ca1—O2^viii^	92.8 (4)	O2^xv^—Ca2—O4	151.9 (4)
Eu2^iv^—Ca1—Eu2^v^	102.95 (9)	O3^xiii^—Ca2—O3^xvi^	137.4 (4)
Eu2^iv^—Ca1—Eu2^vi^	157.62 (7)	O3^xiii^—Ca2—O4	103.97 (19)
Eu2^iv^—Ca1—Eu2^vii^	96.04 (5)	O3^xvi^—Ca2—O4	103.97 (19)
Eu2^iv^—Ca1—Eu2^viii^	60.71 (4)	Ca1^xvii^—Eu2—Ca1^xviii^	104.79 (6)
Eu2^iv^—Ca1—Si1^vi^	111.59 (13)	Ca1^xvii^—Eu2—Ca1^xix^	83.96 (5)
Eu2^iv^—Ca1—Si1^vii^	144.17 (14)	Ca1^xvii^—Eu2—Ca1^xx^	50.79 (11)
Eu2^iv^—Ca1—Si1^viii^	60.87 (6)	Ca1^xvii^—Eu2—Eu1^xvii^	0
Eu2^iv^—Ca1—O1	40.7 (2)	Ca1^xvii^—Eu2—Eu1^xviii^	104.79 (6)
Eu2^iv^—Ca1—O1^ix^	106.9 (3)	Ca1^xvii^—Eu2—Eu1^xix^	83.96 (5)
Eu2^iv^—Ca1—O1^x^	62.5 (3)	Ca1^xvii^—Eu2—Eu1^xx^	50.79 (11)
Eu2^iv^—Ca1—O2^vi^	92.76 (19)	Ca1^xvii^—Eu2—Ca2	0
Eu2^iv^—Ca1—O2^vii^	159.7 (2)	Ca1^xvii^—Eu2—Ca2^iv^	150.53 (7)
Eu2^iv^—Ca1—O2^viii^	85.6 (2)	Ca1^xvii^—Eu2—Ca2^xi^	103.10 (5)
Eu2^v^—Ca1—Eu2^vi^	60.71 (3)	Ca1^xvii^—Eu2—Ca2^xii^	60.59 (8)
Eu2^v^—Ca1—Eu2^vii^	157.62 (4)	Ca1^xvii^—Eu2—Ca2^xiii^	103.26 (10)
Eu2^v^—Ca1—Eu2^viii^	96.04 (5)	Ca1^xvii^—Eu2—Ca2^viii^	96.58 (8)
Eu2^v^—Ca1—Si1^vi^	60.87 (7)	Ca1^xvii^—Eu2—Ca2^xiv^	146.60 (9)
Eu2^v^—Ca1—Si1^vii^	111.59 (11)	Ca1^xvii^—Eu2—Eu2^iv^	150.53 (7)
Eu2^v^—Ca1—Si1^viii^	144.17 (18)	Ca1^xvii^—Eu2—Eu2^xi^	103.10 (5)
Eu2^v^—Ca1—O1	62.5 (2)	Ca1^xvii^—Eu2—Eu2^xii^	60.59 (8)
Eu2^v^—Ca1—O1^ix^	40.7 (4)	Ca1^xvii^—Eu2—Eu2^xiii^	103.26 (10)
Eu2^v^—Ca1—O1^x^	106.9 (3)	Ca1^xvii^—Eu2—Eu2^viii^	96.58 (8)
Eu2^v^—Ca1—O2^vi^	85.6 (2)	Ca1^xvii^—Eu2—Eu2^xiv^	146.60 (9)
Eu2^v^—Ca1—O2^vii^	92.8 (3)	Ca1^xvii^—Eu2—Si1	134.45 (12)
Eu2^v^—Ca1—O2^viii^	159.7 (3)	Ca1^xvii^—Eu2—Si1^xi^	57.83 (17)
Eu2^vi^—Ca1—Eu2^vii^	103.60 (9)	Ca1^xvii^—Eu2—O2^xv^	75.2 (4)
Eu2^vi^—Ca1—Eu2^viii^	103.60 (9)	Ca1^xvii^—Eu2—O3^xiii^	94.1 (3)
Eu2^vi^—Ca1—Si1^vi^	48.22 (12)	Ca1^xvii^—Eu2—O3^xvi^	43.3 (3)
Eu2^vi^—Ca1—Si1^vii^	56.79 (14)	Ca1^xvii^—Eu2—O4	129.31 (6)
Eu2^vi^—Ca1—Si1^viii^	122.41 (17)	Ca1^xviii^—Eu2—Ca1^xix^	49.70 (10)
Eu2^vi^—Ca1—O1	122.0 (3)	Ca1^xviii^—Eu2—Ca1^xx^	83.96 (5)
Eu2^vi^—Ca1—O1^ix^	71.61 (16)	Ca1^xviii^—Eu2—Eu1^xvii^	104.79 (6)
Eu2^vi^—Ca1—O1^x^	134.2 (2)	Ca1^xviii^—Eu2—Eu1^xviii^	0
Eu2^vi^—Ca1—O2^vi^	71.9 (2)	Ca1^xviii^—Eu2—Eu1^xix^	49.70 (10)
Eu2^vi^—Ca1—O2^vii^	32.2 (3)	Ca1^xviii^—Eu2—Eu1^xx^	83.96 (5)
Eu2^vi^—Ca1—O2^viii^	105.4 (2)	Ca1^xviii^—Eu2—Ca2	0
Eu2^vii^—Ca1—Eu2^viii^	103.60 (10)	Ca1^xviii^—Eu2—Ca2^iv^	99.26 (6)
Eu2^vii^—Ca1—Si1^vi^	122.41 (17)	Ca1^xviii^—Eu2—Ca2^xi^	148.63 (8)
Eu2^vii^—Ca1—Si1^vii^	48.22 (13)	Ca1^xviii^—Eu2—Ca2^xii^	146.90 (8)
Eu2^vii^—Ca1—Si1^viii^	56.79 (18)	Ca1^xviii^—Eu2—Ca2^xiii^	97.57 (7)
Eu2^vii^—Ca1—O1	134.2 (3)	Ca1^xviii^—Eu2—Ca2^viii^	100.73 (8)
Eu2^vii^—Ca1—O1^ix^	122.0 (4)	Ca1^xviii^—Eu2—Ca2^xiv^	58.69 (7)
Eu2^vii^—Ca1—O1^x^	71.6 (3)	Ca1^xviii^—Eu2—Eu2^iv^	99.26 (6)
Eu2^vii^—Ca1—O2^vi^	105.4 (3)	Ca1^xviii^—Eu2—Eu2^xi^	148.63 (8)
Eu2^vii^—Ca1—O2^vii^	71.9 (3)	Ca1^xviii^—Eu2—Eu2^xii^	146.90 (8)
Eu2^vii^—Ca1—O2^viii^	32.2 (4)	Ca1^xviii^—Eu2—Eu2^xiii^	97.57 (7)
Eu2^viii^—Ca1—Si1^vi^	56.79 (12)	Ca1^xviii^—Eu2—Eu2^viii^	100.73 (8)
Eu2^viii^—Ca1—Si1^vii^	122.41 (18)	Ca1^xviii^—Eu2—Eu2^xiv^	58.69 (7)
Eu2^viii^—Ca1—Si1^viii^	48.22 (19)	Ca1^xviii^—Eu2—Si1	50.56 (12)
Eu2^viii^—Ca1—O1	71.6 (2)	Ca1^xviii^—Eu2—Si1^xi^	141.16 (16)
Eu2^viii^—Ca1—O1^ix^	134.2 (4)	Ca1^xviii^—Eu2—O2^xv^	32.5 (3)
Eu2^viii^—Ca1—O1^x^	122.0 (2)	Ca1^xviii^—Eu2—O3^xiii^	69.84 (15)
Eu2^viii^—Ca1—O2^vi^	32.23 (19)	Ca1^xviii^—Eu2—O3^xvi^	116.00 (15)
Eu2^viii^—Ca1—O2^vii^	105.4 (3)	Ca1^xviii^—Eu2—O4	125.87 (9)
Eu2^viii^—Ca1—O2^viii^	71.9 (4)	Ca1^xix^—Eu2—Ca1^xx^	104.79 (6)
Si1^vi^—Ca1—Si1^vii^	93.8 (2)	Ca1^xix^—Eu2—Eu1^xvii^	83.96 (5)
Si1^vi^—Ca1—Si1^viii^	93.8 (2)	Ca1^xix^—Eu2—Eu1^xviii^	49.70 (10)
Si1^vi^—Ca1—O1	93.7 (3)	Ca1^xix^—Eu2—Eu1^xix^	0
Si1^vi^—Ca1—O1^ix^	97.5 (3)	Ca1^xix^—Eu2—Eu1^xx^	104.79 (6)
Si1^vi^—Ca1—O1^x^	165.9 (4)	Ca1^xix^—Eu2—Ca2	0
Si1^vi^—Ca1—O2^vi^	28.9 (2)	Ca1^xix^—Eu2—Ca2^iv^	99.26 (6)
Si1^vi^—Ca1—O2^vii^	65.1 (4)	Ca1^xix^—Eu2—Ca2^xi^	148.63 (8)
Si1^vi^—Ca1—O2^viii^	98.9 (4)	Ca1^xix^—Eu2—Ca2^xii^	97.57 (7)
Si1^vii^—Ca1—Si1^viii^	93.79 (18)	Ca1^xix^—Eu2—Ca2^xiii^	146.90 (8)
Si1^vii^—Ca1—O1	165.9 (3)	Ca1^xix^—Eu2—Ca2^viii^	58.69 (7)
Si1^vii^—Ca1—O1^ix^	93.7 (4)	Ca1^xix^—Eu2—Ca2^xiv^	100.73 (8)
Si1^vii^—Ca1—O1^x^	97.5 (3)	Ca1^xix^—Eu2—Eu2^iv^	99.26 (6)
Si1^vii^—Ca1—O2^vi^	98.9 (3)	Ca1^xix^—Eu2—Eu2^xi^	148.63 (8)
Si1^vii^—Ca1—O2^vii^	28.9 (4)	Ca1^xix^—Eu2—Eu2^xii^	97.57 (7)
Si1^vii^—Ca1—O2^viii^	65.1 (3)	Ca1^xix^—Eu2—Eu2^xiii^	146.90 (8)
Si1^viii^—Ca1—O1	97.5 (2)	Ca1^xix^—Eu2—Eu2^viii^	58.69 (7)
Si1^viii^—Ca1—O1^ix^	165.9 (2)	Ca1^xix^—Eu2—Eu2^xiv^	100.73 (8)
Si1^viii^—Ca1—O1^x^	93.7 (3)	Ca1^xix^—Eu2—Si1	50.56 (12)
Si1^viii^—Ca1—O2^vi^	65.1 (3)	Ca1^xix^—Eu2—Si1^xi^	141.16 (16)
Si1^viii^—Ca1—O2^vii^	98.9 (2)	Ca1^xix^—Eu2—O2^xv^	32.5 (3)
Si1^viii^—Ca1—O2^viii^	28.9 (3)	Ca1^xix^—Eu2—O3^xiii^	116.00 (15)
O1—Ca1—O1^ix^	73.5 (4)	Ca1^xix^—Eu2—O3^xvi^	69.84 (15)
O1—Ca1—O1^x^	73.5 (4)	Ca1^xix^—Eu2—O4	125.87 (9)
O1—Ca1—O2^vi^	93.4 (3)	Ca1^xx^—Eu2—Eu1^xvii^	50.79 (11)
O1—Ca1—O2^vii^	153.9 (4)	Ca1^xx^—Eu2—Eu1^xviii^	83.96 (5)
O1—Ca1—O2^viii^	125.2 (3)	Ca1^xx^—Eu2—Eu1^xix^	104.79 (6)
O1^ix^—Ca1—O1^x^	73.5 (4)	Ca1^xx^—Eu2—Eu1^xx^	0
O1^ix^—Ca1—O2^vi^	125.2 (4)	Ca1^xx^—Eu2—Ca2	0
O1^ix^—Ca1—O2^vii^	93.4 (3)	Ca1^xx^—Eu2—Ca2^iv^	150.53 (7)
O1^ix^—Ca1—O2^viii^	153.9 (5)	Ca1^xx^—Eu2—Ca2^xi^	103.10 (5)
O1^x^—Ca1—O2^vi^	153.9 (3)	Ca1^xx^—Eu2—Ca2^xii^	103.26 (10)
O1^x^—Ca1—O2^vii^	125.2 (5)	Ca1^xx^—Eu2—Ca2^xiii^	60.59 (8)
O1^x^—Ca1—O2^viii^	93.4 (4)	Ca1^xx^—Eu2—Ca2^viii^	146.60 (9)
O2^vi^—Ca1—O2^vii^	75.6 (4)	Ca1^xx^—Eu2—Ca2^xiv^	96.58 (8)
O2^vi^—Ca1—O2^viii^	75.6 (5)	Ca1^xx^—Eu2—Eu2^iv^	150.53 (7)
O2^vii^—Ca1—O2^viii^	75.6 (3)	Ca1^xx^—Eu2—Eu2^xi^	103.10 (5)
Ca1—Eu1—Ca1^i^	0	Ca1^xx^—Eu2—Eu2^xii^	103.26 (10)
Ca1—Eu1—Ca1^ii^	0	Ca1^xx^—Eu2—Eu2^xiii^	60.59 (8)
Ca1—Eu1—Eu1^i^	0	Ca1^xx^—Eu2—Eu2^viii^	146.60 (9)
Ca1—Eu1—Eu1^ii^	0	Ca1^xx^—Eu2—Eu2^xiv^	96.58 (8)
Ca1—Eu1—Ca2^iii^	0	Ca1^xx^—Eu2—Si1	134.45 (12)
Ca1—Eu1—Ca2^iv^	0	Ca1^xx^—Eu2—Si1^xi^	57.83 (17)
Ca1—Eu1—Ca2^v^	0	Ca1^xx^—Eu2—O2^xv^	75.2 (4)
Ca1—Eu1—Ca2^vi^	0	Ca1^xx^—Eu2—O3^xiii^	43.3 (3)
Ca1—Eu1—Ca2^vii^	0	Ca1^xx^—Eu2—O3^xvi^	94.1 (3)
Ca1—Eu1—Ca2^viii^	0	Ca1^xx^—Eu2—O4	129.31 (6)
Ca1—Eu1—Eu2^iii^	0	Eu1^xvii^—Eu2—Eu1^xviii^	104.79 (6)
Ca1—Eu1—Eu2^iv^	0	Eu1^xvii^—Eu2—Eu1^xix^	83.96 (5)
Ca1—Eu1—Eu2^v^	0	Eu1^xvii^—Eu2—Eu1^xx^	50.79 (11)
Ca1—Eu1—Eu2^vi^	0	Eu1^xvii^—Eu2—Ca2	0
Ca1—Eu1—Eu2^vii^	0	Eu1^xvii^—Eu2—Ca2^iv^	150.53 (7)
Ca1—Eu1—Eu2^viii^	0	Eu1^xvii^—Eu2—Ca2^xi^	103.10 (5)
Ca1—Eu1—Si1^vi^	0	Eu1^xvii^—Eu2—Ca2^xii^	60.59 (8)
Ca1—Eu1—Si1^vii^	0	Eu1^xvii^—Eu2—Ca2^xiii^	103.26 (10)
Ca1—Eu1—Si1^viii^	0	Eu1^xvii^—Eu2—Ca2^viii^	96.58 (8)
Ca1—Eu1—O1	0	Eu1^xvii^—Eu2—Ca2^xiv^	146.60 (9)
Ca1—Eu1—O1^ix^	0	Eu1^xvii^—Eu2—Eu2^iv^	150.53 (7)
Ca1—Eu1—O1^x^	0	Eu1^xvii^—Eu2—Eu2^xi^	103.10 (5)
Ca1^i^—Eu1—Ca1^ii^	180	Eu1^xvii^—Eu2—Eu2^xii^	60.59 (8)
Ca1^i^—Eu1—Eu1^i^	0	Eu1^xvii^—Eu2—Eu2^xiii^	103.26 (10)
Ca1^i^—Eu1—Eu1^ii^	180	Eu1^xvii^—Eu2—Eu2^viii^	96.58 (8)
Ca1^i^—Eu1—Ca2^iii^	115.39 (7)	Eu1^xvii^—Eu2—Eu2^xiv^	146.60 (9)
Ca1^i^—Eu1—Ca2^iv^	115.39 (7)	Eu1^xvii^—Eu2—Si1	134.45 (12)
Ca1^i^—Eu1—Ca2^v^	115.39 (7)	Eu1^xvii^—Eu2—Si1^xi^	57.83 (17)
Ca1^i^—Eu1—Ca2^vi^	65.15 (7)	Eu1^xvii^—Eu2—O2^xv^	75.2 (4)
Ca1^i^—Eu1—Ca2^vii^	65.15 (7)	Eu1^xvii^—Eu2—O3^xiii^	94.1 (3)
Ca1^i^—Eu1—Ca2^viii^	65.15 (7)	Eu1^xvii^—Eu2—O3^xvi^	43.3 (3)
Ca1^i^—Eu1—Eu2^iii^	115.39 (7)	Eu1^xvii^—Eu2—O4	129.31 (6)
Ca1^i^—Eu1—Eu2^iv^	115.39 (7)	Eu1^xviii^—Eu2—Eu1^xix^	49.70 (10)
Ca1^i^—Eu1—Eu2^v^	115.39 (7)	Eu1^xviii^—Eu2—Eu1^xx^	83.96 (5)
Ca1^i^—Eu1—Eu2^vi^	65.15 (7)	Eu1^xviii^—Eu2—Ca2	0
Ca1^i^—Eu1—Eu2^vii^	65.15 (7)	Eu1^xviii^—Eu2—Ca2^iv^	99.26 (6)
Ca1^i^—Eu1—Eu2^viii^	65.15 (7)	Eu1^xviii^—Eu2—Ca2^xi^	148.63 (8)
Ca1^i^—Eu1—Si1^vi^	57.47 (11)	Eu1^xviii^—Eu2—Ca2^xii^	146.90 (8)
Ca1^i^—Eu1—Si1^vii^	57.47 (13)	Eu1^xviii^—Eu2—Ca2^xiii^	97.57 (7)
Ca1^i^—Eu1—Si1^viii^	57.47 (10)	Eu1^xviii^—Eu2—Ca2^viii^	100.73 (8)
Ca1^i^—Eu1—O1	136.3 (2)	Eu1^xviii^—Eu2—Ca2^xiv^	58.69 (7)
Ca1^i^—Eu1—O1^ix^	136.30 (19)	Eu1^xviii^—Eu2—Eu2^iv^	99.26 (6)
Ca1^i^—Eu1—O1^x^	136.3 (3)	Eu1^xviii^—Eu2—Eu2^xi^	148.63 (8)
Ca1^ii^—Eu1—Eu1^i^	180	Eu1^xviii^—Eu2—Eu2^xii^	146.90 (8)
Ca1^ii^—Eu1—Eu1^ii^	0	Eu1^xviii^—Eu2—Eu2^xiii^	97.57 (7)
Ca1^ii^—Eu1—Ca2^iii^	64.61 (7)	Eu1^xviii^—Eu2—Eu2^viii^	100.73 (8)
Ca1^ii^—Eu1—Ca2^iv^	64.61 (7)	Eu1^xviii^—Eu2—Eu2^xiv^	58.69 (7)
Ca1^ii^—Eu1—Ca2^v^	64.61 (7)	Eu1^xviii^—Eu2—Si1	50.56 (12)
Ca1^ii^—Eu1—Ca2^vi^	114.85 (7)	Eu1^xviii^—Eu2—Si1^xi^	141.16 (16)
Ca1^ii^—Eu1—Ca2^vii^	114.85 (7)	Eu1^xviii^—Eu2—O2^xv^	32.5 (3)
Ca1^ii^—Eu1—Ca2^viii^	114.85 (7)	Eu1^xviii^—Eu2—O3^xiii^	69.84 (15)
Ca1^ii^—Eu1—Eu2^iii^	64.61 (7)	Eu1^xviii^—Eu2—O3^xvi^	116.00 (15)
Ca1^ii^—Eu1—Eu2^iv^	64.61 (7)	Eu1^xviii^—Eu2—O4	125.87 (9)
Ca1^ii^—Eu1—Eu2^v^	64.61 (7)	Eu1^xix^—Eu2—Eu1^xx^	104.79 (6)
Ca1^ii^—Eu1—Eu2^vi^	114.85 (7)	Eu1^xix^—Eu2—Ca2	0
Ca1^ii^—Eu1—Eu2^vii^	114.85 (7)	Eu1^xix^—Eu2—Ca2^iv^	99.26 (6)
Ca1^ii^—Eu1—Eu2^viii^	114.85 (7)	Eu1^xix^—Eu2—Ca2^xi^	148.63 (8)
Ca1^ii^—Eu1—Si1^vi^	122.53 (11)	Eu1^xix^—Eu2—Ca2^xii^	97.57 (7)
Ca1^ii^—Eu1—Si1^vii^	122.53 (13)	Eu1^xix^—Eu2—Ca2^xiii^	146.90 (8)
Ca1^ii^—Eu1—Si1^viii^	122.53 (10)	Eu1^xix^—Eu2—Ca2^viii^	58.69 (7)
Ca1^ii^—Eu1—O1	43.7 (2)	Eu1^xix^—Eu2—Ca2^xiv^	100.73 (8)
Ca1^ii^—Eu1—O1^ix^	43.70 (19)	Eu1^xix^—Eu2—Eu2^iv^	99.26 (6)
Ca1^ii^—Eu1—O1^x^	43.7 (3)	Eu1^xix^—Eu2—Eu2^xi^	148.63 (8)
Eu1^i^—Eu1—Eu1^ii^	180	Eu1^xix^—Eu2—Eu2^xii^	97.57 (7)
Eu1^i^—Eu1—Ca2^iii^	115.39 (7)	Eu1^xix^—Eu2—Eu2^xiii^	146.90 (8)
Eu1^i^—Eu1—Ca2^iv^	115.39 (7)	Eu1^xix^—Eu2—Eu2^viii^	58.69 (7)
Eu1^i^—Eu1—Ca2^v^	115.39 (7)	Eu1^xix^—Eu2—Eu2^xiv^	100.73 (8)
Eu1^i^—Eu1—Ca2^vi^	65.15 (7)	Eu1^xix^—Eu2—Si1	50.56 (12)
Eu1^i^—Eu1—Ca2^vii^	65.15 (7)	Eu1^xix^—Eu2—Si1^xi^	141.16 (16)
Eu1^i^—Eu1—Ca2^viii^	65.15 (7)	Eu1^xix^—Eu2—O2^xv^	32.5 (3)
Eu1^i^—Eu1—Eu2^iii^	115.39 (7)	Eu1^xix^—Eu2—O3^xiii^	116.00 (15)
Eu1^i^—Eu1—Eu2^iv^	115.39 (7)	Eu1^xix^—Eu2—O3^xvi^	69.84 (15)
Eu1^i^—Eu1—Eu2^v^	115.39 (7)	Eu1^xix^—Eu2—O4	125.87 (9)
Eu1^i^—Eu1—Eu2^vi^	65.15 (7)	Eu1^xx^—Eu2—Ca2	0
Eu1^i^—Eu1—Eu2^vii^	65.15 (7)	Eu1^xx^—Eu2—Ca2^iv^	150.53 (7)
Eu1^i^—Eu1—Eu2^viii^	65.15 (7)	Eu1^xx^—Eu2—Ca2^xi^	103.10 (5)
Eu1^i^—Eu1—Si1^vi^	57.47 (11)	Eu1^xx^—Eu2—Ca2^xii^	103.26 (10)
Eu1^i^—Eu1—Si1^vii^	57.47 (13)	Eu1^xx^—Eu2—Ca2^xiii^	60.59 (8)
Eu1^i^—Eu1—Si1^viii^	57.47 (10)	Eu1^xx^—Eu2—Ca2^viii^	146.60 (9)
Eu1^i^—Eu1—O1	136.3 (2)	Eu1^xx^—Eu2—Ca2^xiv^	96.58 (8)
Eu1^i^—Eu1—O1^ix^	136.30 (19)	Eu1^xx^—Eu2—Eu2^iv^	150.53 (7)
Eu1^i^—Eu1—O1^x^	136.3 (3)	Eu1^xx^—Eu2—Eu2^xi^	103.10 (5)
Eu1^ii^—Eu1—Ca2^iii^	64.61 (7)	Eu1^xx^—Eu2—Eu2^xii^	103.26 (10)
Eu1^ii^—Eu1—Ca2^iv^	64.61 (7)	Eu1^xx^—Eu2—Eu2^xiii^	60.59 (8)
Eu1^ii^—Eu1—Ca2^v^	64.61 (7)	Eu1^xx^—Eu2—Eu2^viii^	146.60 (9)
Eu1^ii^—Eu1—Ca2^vi^	114.85 (7)	Eu1^xx^—Eu2—Eu2^xiv^	96.58 (8)
Eu1^ii^—Eu1—Ca2^vii^	114.85 (7)	Eu1^xx^—Eu2—Si1	134.45 (12)
Eu1^ii^—Eu1—Ca2^viii^	114.85 (7)	Eu1^xx^—Eu2—Si1^xi^	57.83 (17)
Eu1^ii^—Eu1—Eu2^iii^	64.61 (7)	Eu1^xx^—Eu2—O2^xv^	75.2 (4)
Eu1^ii^—Eu1—Eu2^iv^	64.61 (7)	Eu1^xx^—Eu2—O3^xiii^	43.3 (3)
Eu1^ii^—Eu1—Eu2^v^	64.61 (7)	Eu1^xx^—Eu2—O3^xvi^	94.1 (3)
Eu1^ii^—Eu1—Eu2^vi^	114.85 (7)	Eu1^xx^—Eu2—O4	129.31 (6)
Eu1^ii^—Eu1—Eu2^vii^	114.85 (7)	Ca2—Eu2—Ca2^iv^	0
Eu1^ii^—Eu1—Eu2^viii^	114.85 (7)	Ca2—Eu2—Ca2^xi^	0
Eu1^ii^—Eu1—Si1^vi^	122.53 (11)	Ca2—Eu2—Ca2^xii^	0
Eu1^ii^—Eu1—Si1^vii^	122.53 (13)	Ca2—Eu2—Ca2^xiii^	0
Eu1^ii^—Eu1—Si1^viii^	122.53 (10)	Ca2—Eu2—Ca2^viii^	0
Eu1^ii^—Eu1—O1	43.7 (2)	Ca2—Eu2—Ca2^xiv^	0
Eu1^ii^—Eu1—O1^ix^	43.70 (19)	Ca2—Eu2—Eu2^iv^	0
Eu1^ii^—Eu1—O1^x^	43.7 (3)	Ca2—Eu2—Eu2^xi^	0
Ca2^iii^—Eu1—Ca2^iv^	102.95 (10)	Ca2—Eu2—Eu2^xii^	0
Ca2^iii^—Eu1—Ca2^v^	102.95 (9)	Ca2—Eu2—Eu2^xiii^	0
Ca2^iii^—Eu1—Ca2^vi^	96.04 (4)	Ca2—Eu2—Eu2^viii^	0
Ca2^iii^—Eu1—Ca2^vii^	60.71 (4)	Ca2—Eu2—Eu2^xiv^	0
Ca2^iii^—Eu1—Ca2^viii^	157.62 (5)	Ca2—Eu2—Si1	0
Ca2^iii^—Eu1—Eu2^iii^	0	Ca2—Eu2—Si1^xi^	0
Ca2^iii^—Eu1—Eu2^iv^	102.95 (10)	Ca2—Eu2—O2^xv^	0
Ca2^iii^—Eu1—Eu2^v^	102.95 (9)	Ca2—Eu2—O3^xiii^	0
Ca2^iii^—Eu1—Eu2^vi^	96.04 (4)	Ca2—Eu2—O3^xvi^	0
Ca2^iii^—Eu1—Eu2^vii^	60.71 (4)	Ca2—Eu2—O4	0
Ca2^iii^—Eu1—Eu2^viii^	157.62 (5)	Ca2^iv^—Eu2—Ca2^xi^	60.00 (6)
Ca2^iii^—Eu1—Si1^vi^	144.17 (11)	Ca2^iv^—Eu2—Ca2^xii^	90.00 (7)
Ca2^iii^—Eu1—Si1^vii^	60.87 (12)	Ca2^iv^—Eu2—Ca2^xiii^	90.00 (7)
Ca2^iii^—Eu1—Si1^viii^	111.59 (18)	Ca2^iv^—Eu2—Ca2^viii^	61.96 (6)
Ca2^iii^—Eu1—O1	106.9 (3)	Ca2^iv^—Eu2—Ca2^xiv^	61.96 (6)
Ca2^iii^—Eu1—O1^ix^	62.5 (4)	Ca2^iv^—Eu2—Eu2^iv^	0
Ca2^iii^—Eu1—O1^x^	40.7 (3)	Ca2^iv^—Eu2—Eu2^xi^	60.00 (6)
Ca2^iv^—Eu1—Ca2^v^	102.95 (9)	Ca2^iv^—Eu2—Eu2^xii^	90.00 (7)
Ca2^iv^—Eu1—Ca2^vi^	157.62 (7)	Ca2^iv^—Eu2—Eu2^xiii^	90.00 (7)
Ca2^iv^—Eu1—Ca2^vii^	96.04 (5)	Ca2^iv^—Eu2—Eu2^viii^	61.96 (6)
Ca2^iv^—Eu1—Ca2^viii^	60.71 (4)	Ca2^iv^—Eu2—Eu2^xiv^	61.96 (6)
Ca2^iv^—Eu1—Eu2^iii^	102.95 (10)	Ca2^iv^—Eu2—Si1	54.66 (15)
Ca2^iv^—Eu1—Eu2^iv^	0	Ca2^iv^—Eu2—Si1^xi^	110.6 (2)
Ca2^iv^—Eu1—Eu2^v^	102.95 (9)	Ca2^iv^—Eu2—O2^xv^	121.9 (4)
Ca2^iv^—Eu1—Eu2^vi^	157.62 (7)	Ca2^iv^—Eu2—O3^xiii^	110.2 (2)
Ca2^iv^—Eu1—Eu2^vii^	96.04 (5)	Ca2^iv^—Eu2—O3^xvi^	110.2 (2)
Ca2^iv^—Eu1—Eu2^viii^	60.71 (4)	Ca2^iv^—Eu2—O4	30.00 (5)
Ca2^iv^—Eu1—Si1^vi^	111.59 (13)	Ca2^xi^—Eu2—Ca2^xii^	61.96 (4)
Ca2^iv^—Eu1—Si1^vii^	144.17 (14)	Ca2^xi^—Eu2—Ca2^xiii^	61.96 (4)
Ca2^iv^—Eu1—Si1^viii^	60.87 (6)	Ca2^xi^—Eu2—Ca2^viii^	90.00 (6)
Ca2^iv^—Eu1—O1	40.7 (2)	Ca2^xi^—Eu2—Ca2^xiv^	90.00 (6)
Ca2^iv^—Eu1—O1^ix^	106.9 (3)	Ca2^xi^—Eu2—Eu2^iv^	60.00 (6)
Ca2^iv^—Eu1—O1^x^	62.5 (3)	Ca2^xi^—Eu2—Eu2^xi^	0
Ca2^v^—Eu1—Ca2^vi^	60.71 (3)	Ca2^xi^—Eu2—Eu2^xii^	61.96 (4)
Ca2^v^—Eu1—Ca2^vii^	157.62 (4)	Ca2^xi^—Eu2—Eu2^xiii^	61.96 (4)
Ca2^v^—Eu1—Ca2^viii^	96.04 (5)	Ca2^xi^—Eu2—Eu2^viii^	90.00 (6)
Ca2^v^—Eu1—Eu2^iii^	102.95 (9)	Ca2^xi^—Eu2—Eu2^xiv^	90.00 (6)
Ca2^v^—Eu1—Eu2^iv^	102.95 (9)	Ca2^xi^—Eu2—Si1	114.66 (16)
Ca2^v^—Eu1—Eu2^v^	0	Ca2^xi^—Eu2—Si1^xi^	50.64 (19)
Ca2^v^—Eu1—Eu2^vi^	60.71 (3)	Ca2^xi^—Eu2—O2^xv^	178.1 (4)
Ca2^v^—Eu1—Eu2^vii^	157.62 (4)	Ca2^xi^—Eu2—O3^xiii^	94.20 (14)
Ca2^v^—Eu1—Eu2^viii^	96.04 (5)	Ca2^xi^—Eu2—O3^xvi^	94.20 (14)
Ca2^v^—Eu1—Si1^vi^	60.87 (7)	Ca2^xi^—Eu2—O4	30.00 (4)
Ca2^v^—Eu1—Si1^vii^	111.59 (11)	Ca2^xii^—Eu2—Ca2^xiii^	114.25 (5)
Ca2^v^—Eu1—Si1^viii^	144.17 (18)	Ca2^xii^—Eu2—Ca2^viii^	56.08 (4)
Ca2^v^—Eu1—O1	62.5 (2)	Ca2^xii^—Eu2—Ca2^xiv^	148.50 (9)
Ca2^v^—Eu1—O1^ix^	40.7 (4)	Ca2^xii^—Eu2—Eu2^iv^	90.00 (7)
Ca2^v^—Eu1—O1^x^	106.9 (3)	Ca2^xii^—Eu2—Eu2^xi^	61.96 (4)
Ca2^vi^—Eu1—Ca2^vii^	103.60 (9)	Ca2^xii^—Eu2—Eu2^xii^	0
Ca2^vi^—Eu1—Ca2^viii^	103.60 (9)	Ca2^xii^—Eu2—Eu2^xiii^	114.25 (5)
Ca2^vi^—Eu1—Eu2^iii^	96.04 (4)	Ca2^xii^—Eu2—Eu2^viii^	56.08 (4)
Ca2^vi^—Eu1—Eu2^iv^	157.62 (7)	Ca2^xii^—Eu2—Eu2^xiv^	148.50 (9)
Ca2^vi^—Eu1—Eu2^v^	60.71 (3)	Ca2^xii^—Eu2—Si1	116.28 (8)
Ca2^vi^—Eu1—Eu2^vi^	0	Ca2^xii^—Eu2—Si1^xi^	59.47 (6)
Ca2^vi^—Eu1—Eu2^vii^	103.60 (9)	Ca2^xii^—Eu2—O2^xv^	117.43 (14)
Ca2^vi^—Eu1—Eu2^viii^	103.60 (9)	Ca2^xii^—Eu2—O3^xiii^	136.1 (2)
Ca2^vi^—Eu1—Si1^vi^	48.22 (12)	Ca2^xii^—Eu2—O3^xvi^	32.37 (15)
Ca2^vi^—Eu1—Si1^vii^	56.79 (14)	Ca2^xii^—Eu2—O4	74.25 (5)
Ca2^vi^—Eu1—Si1^viii^	122.41 (17)	Ca2^xiii^—Eu2—Ca2^viii^	148.50 (9)
Ca2^vi^—Eu1—O1	122.0 (3)	Ca2^xiii^—Eu2—Ca2^xiv^	56.08 (4)
Ca2^vi^—Eu1—O1^ix^	71.61 (16)	Ca2^xiii^—Eu2—Eu2^iv^	90.00 (7)
Ca2^vi^—Eu1—O1^x^	134.2 (2)	Ca2^xiii^—Eu2—Eu2^xi^	61.96 (4)
Ca2^vii^—Eu1—Ca2^viii^	103.60 (10)	Ca2^xiii^—Eu2—Eu2^xii^	114.25 (5)
Ca2^vii^—Eu1—Eu2^iii^	60.71 (4)	Ca2^xiii^—Eu2—Eu2^xiii^	0
Ca2^vii^—Eu1—Eu2^iv^	96.04 (5)	Ca2^xiii^—Eu2—Eu2^viii^	148.50 (9)
Ca2^vii^—Eu1—Eu2^v^	157.62 (4)	Ca2^xiii^—Eu2—Eu2^xiv^	56.08 (4)
Ca2^vii^—Eu1—Eu2^vi^	103.60 (9)	Ca2^xiii^—Eu2—Si1	116.28 (8)
Ca2^vii^—Eu1—Eu2^vii^	0	Ca2^xiii^—Eu2—Si1^xi^	59.47 (6)
Ca2^vii^—Eu1—Eu2^viii^	103.60 (10)	Ca2^xiii^—Eu2—O2^xv^	117.43 (14)
Ca2^vii^—Eu1—Si1^vi^	122.41 (17)	Ca2^xiii^—Eu2—O3^xiii^	32.37 (15)
Ca2^vii^—Eu1—Si1^vii^	48.22 (13)	Ca2^xiii^—Eu2—O3^xvi^	136.1 (2)
Ca2^vii^—Eu1—Si1^viii^	56.79 (18)	Ca2^xiii^—Eu2—O4	74.25 (5)
Ca2^vii^—Eu1—O1	134.2 (3)	Ca2^viii^—Eu2—Ca2^xiv^	114.25 (8)
Ca2^vii^—Eu1—O1^ix^	122.0 (4)	Ca2^viii^—Eu2—Eu2^iv^	61.96 (6)
Ca2^vii^—Eu1—O1^x^	71.6 (3)	Ca2^viii^—Eu2—Eu2^xi^	90.00 (6)
Ca2^viii^—Eu1—Eu2^iii^	157.62 (5)	Ca2^viii^—Eu2—Eu2^xii^	56.08 (4)
Ca2^viii^—Eu1—Eu2^iv^	60.71 (4)	Ca2^viii^—Eu2—Eu2^xiii^	148.50 (9)
Ca2^viii^—Eu1—Eu2^v^	96.04 (5)	Ca2^viii^—Eu2—Eu2^viii^	0
Ca2^viii^—Eu1—Eu2^vi^	103.60 (9)	Ca2^viii^—Eu2—Eu2^xiv^	114.25 (8)
Ca2^viii^—Eu1—Eu2^vii^	103.60 (10)	Ca2^viii^—Eu2—Si1	60.44 (5)
Ca2^viii^—Eu1—Eu2^viii^	0	Ca2^viii^—Eu2—Si1^xi^	114.82 (8)
Ca2^viii^—Eu1—Si1^vi^	56.79 (12)	Ca2^viii^—Eu2—O2^xv^	91.0 (2)
Ca2^viii^—Eu1—Si1^vii^	122.41 (18)	Ca2^viii^—Eu2—O3^xiii^	167.3 (2)
Ca2^viii^—Eu1—Si1^viii^	48.22 (19)	Ca2^viii^—Eu2—O3^xvi^	53.9 (2)
Ca2^viii^—Eu1—O1	71.6 (2)	Ca2^viii^—Eu2—O4	74.25 (7)
Ca2^viii^—Eu1—O1^ix^	134.2 (4)	Ca2^xiv^—Eu2—Eu2^iv^	61.96 (6)
Ca2^viii^—Eu1—O1^x^	122.0 (2)	Ca2^xiv^—Eu2—Eu2^xi^	90.00 (6)
Eu2^iii^—Eu1—Eu2^iv^	102.95 (10)	Ca2^xiv^—Eu2—Eu2^xii^	148.50 (9)
Eu2^iii^—Eu1—Eu2^v^	102.95 (9)	Ca2^xiv^—Eu2—Eu2^xiii^	56.08 (4)
Eu2^iii^—Eu1—Eu2^vi^	96.04 (4)	Ca2^xiv^—Eu2—Eu2^viii^	114.25 (8)
Eu2^iii^—Eu1—Eu2^vii^	60.71 (4)	Ca2^xiv^—Eu2—Eu2^xiv^	0
Eu2^iii^—Eu1—Eu2^viii^	157.62 (5)	Ca2^xiv^—Eu2—Si1	60.44 (5)
Eu2^iii^—Eu1—Si1^vi^	144.17 (11)	Ca2^xiv^—Eu2—Si1^xi^	114.82 (8)
Eu2^iii^—Eu1—Si1^vii^	60.87 (12)	Ca2^xiv^—Eu2—O2^xv^	91.0 (2)
Eu2^iii^—Eu1—Si1^viii^	111.59 (18)	Ca2^xiv^—Eu2—O3^xiii^	53.9 (2)
Eu2^iii^—Eu1—O1	106.9 (3)	Ca2^xiv^—Eu2—O3^xvi^	167.3 (2)
Eu2^iii^—Eu1—O1^ix^	62.5 (4)	Ca2^xiv^—Eu2—O4	74.25 (7)
Eu2^iii^—Eu1—O1^x^	40.7 (3)	Eu2^iv^—Eu2—Eu2^xi^	60.00 (6)
Eu2^iv^—Eu1—Eu2^v^	102.95 (9)	Eu2^iv^—Eu2—Eu2^xii^	90.00 (7)
Eu2^iv^—Eu1—Eu2^vi^	157.62 (7)	Eu2^iv^—Eu2—Eu2^xiii^	90.00 (7)
Eu2^iv^—Eu1—Eu2^vii^	96.04 (5)	Eu2^iv^—Eu2—Eu2^viii^	61.96 (6)
Eu2^iv^—Eu1—Eu2^viii^	60.71 (4)	Eu2^iv^—Eu2—Eu2^xiv^	61.96 (6)
Eu2^iv^—Eu1—Si1^vi^	111.59 (13)	Eu2^iv^—Eu2—Si1	54.66 (15)
Eu2^iv^—Eu1—Si1^vii^	144.17 (14)	Eu2^iv^—Eu2—Si1^xi^	110.6 (2)
Eu2^iv^—Eu1—Si1^viii^	60.87 (6)	Eu2^iv^—Eu2—O2^xv^	121.9 (4)
Eu2^iv^—Eu1—O1	40.7 (2)	Eu2^iv^—Eu2—O3^xiii^	110.2 (2)
Eu2^iv^—Eu1—O1^ix^	106.9 (3)	Eu2^iv^—Eu2—O3^xvi^	110.2 (2)
Eu2^iv^—Eu1—O1^x^	62.5 (3)	Eu2^iv^—Eu2—O4	30.00 (5)
Eu2^v^—Eu1—Eu2^vi^	60.71 (3)	Eu2^xi^—Eu2—Eu2^xii^	61.96 (4)
Eu2^v^—Eu1—Eu2^vii^	157.62 (4)	Eu2^xi^—Eu2—Eu2^xiii^	61.96 (4)
Eu2^v^—Eu1—Eu2^viii^	96.04 (5)	Eu2^xi^—Eu2—Eu2^viii^	90.00 (6)
Eu2^v^—Eu1—Si1^vi^	60.87 (7)	Eu2^xi^—Eu2—Eu2^xiv^	90.00 (6)
Eu2^v^—Eu1—Si1^vii^	111.59 (11)	Eu2^xi^—Eu2—Si1	114.66 (16)
Eu2^v^—Eu1—Si1^viii^	144.17 (18)	Eu2^xi^—Eu2—Si1^xi^	50.64 (19)
Eu2^v^—Eu1—O1	62.5 (2)	Eu2^xi^—Eu2—O2^xv^	178.1 (4)
Eu2^v^—Eu1—O1^ix^	40.7 (4)	Eu2^xi^—Eu2—O3^xiii^	94.20 (14)
Eu2^v^—Eu1—O1^x^	106.9 (3)	Eu2^xi^—Eu2—O3^xvi^	94.20 (14)
Eu2^vi^—Eu1—Eu2^vii^	103.60 (9)	Eu2^xi^—Eu2—O4	30.00 (4)
Eu2^vi^—Eu1—Eu2^viii^	103.60 (9)	Eu2^xii^—Eu2—Eu2^xiii^	114.25 (5)
Eu2^vi^—Eu1—Si1^vi^	48.22 (12)	Eu2^xii^—Eu2—Eu2^viii^	56.08 (4)
Eu2^vi^—Eu1—Si1^vii^	56.79 (14)	Eu2^xii^—Eu2—Eu2^xiv^	148.50 (9)
Eu2^vi^—Eu1—Si1^viii^	122.41 (17)	Eu2^xii^—Eu2—Si1	116.28 (8)
Eu2^vi^—Eu1—O1	122.0 (3)	Eu2^xii^—Eu2—Si1^xi^	59.47 (6)
Eu2^vi^—Eu1—O1^ix^	71.61 (16)	Eu2^xii^—Eu2—O2^xv^	117.43 (14)
Eu2^vi^—Eu1—O1^x^	134.2 (2)	Eu2^xii^—Eu2—O3^xiii^	136.1 (2)
Eu2^vii^—Eu1—Eu2^viii^	103.60 (10)	Eu2^xii^—Eu2—O3^xvi^	32.37 (15)
Eu2^vii^—Eu1—Si1^vi^	122.41 (17)	Eu2^xii^—Eu2—O4	74.25 (5)
Eu2^vii^—Eu1—Si1^vii^	48.22 (13)	Eu2^xiii^—Eu2—Eu2^viii^	148.50 (9)
Eu2^vii^—Eu1—Si1^viii^	56.79 (18)	Eu2^xiii^—Eu2—Eu2^xiv^	56.08 (4)
Eu2^vii^—Eu1—O1	134.2 (3)	Eu2^xiii^—Eu2—Si1	116.28 (8)
Eu2^vii^—Eu1—O1^ix^	122.0 (4)	Eu2^xiii^—Eu2—Si1^xi^	59.47 (6)
Eu2^vii^—Eu1—O1^x^	71.6 (3)	Eu2^xiii^—Eu2—O2^xv^	117.43 (14)
Eu2^viii^—Eu1—Si1^vi^	56.79 (12)	Eu2^xiii^—Eu2—O3^xiii^	32.37 (15)
Eu2^viii^—Eu1—Si1^vii^	122.41 (18)	Eu2^xiii^—Eu2—O3^xvi^	136.1 (2)
Eu2^viii^—Eu1—Si1^viii^	48.22 (19)	Eu2^xiii^—Eu2—O4	74.25 (5)
Eu2^viii^—Eu1—O1	71.6 (2)	Eu2^viii^—Eu2—Eu2^xiv^	114.25 (8)
Eu2^viii^—Eu1—O1^ix^	134.2 (4)	Eu2^viii^—Eu2—Si1	60.44 (5)
Eu2^viii^—Eu1—O1^x^	122.0 (2)	Eu2^viii^—Eu2—Si1^xi^	114.82 (8)
Si1^vi^—Eu1—Si1^vii^	93.8 (2)	Eu2^viii^—Eu2—O2^xv^	91.0 (2)
Si1^vi^—Eu1—Si1^viii^	93.8 (2)	Eu2^viii^—Eu2—O3^xiii^	167.3 (2)
Si1^vi^—Eu1—O1	93.7 (3)	Eu2^viii^—Eu2—O3^xvi^	53.9 (2)
Si1^vi^—Eu1—O1^ix^	97.5 (3)	Eu2^viii^—Eu2—O4	74.25 (7)
Si1^vi^—Eu1—O1^x^	165.9 (4)	Eu2^xiv^—Eu2—Si1	60.44 (5)
Si1^vii^—Eu1—Si1^viii^	93.79 (18)	Eu2^xiv^—Eu2—Si1^xi^	114.82 (8)
Si1^vii^—Eu1—O1	165.9 (3)	Eu2^xiv^—Eu2—O2^xv^	91.0 (2)
Si1^vii^—Eu1—O1^ix^	93.7 (4)	Eu2^xiv^—Eu2—O3^xiii^	53.9 (2)
Si1^vii^—Eu1—O1^x^	97.5 (3)	Eu2^xiv^—Eu2—O3^xvi^	167.3 (2)
Si1^viii^—Eu1—O1	97.5 (2)	Eu2^xiv^—Eu2—O4	74.25 (7)
Si1^viii^—Eu1—O1^ix^	165.9 (2)	Si1—Eu2—Si1^xi^	165.3 (2)
Si1^viii^—Eu1—O1^x^	93.7 (3)	Si1—Eu2—O2^xv^	67.3 (4)
O1—Eu1—O1^ix^	73.5 (4)	Si1—Eu2—O3^xiii^	107.0 (2)
O1—Eu1—O1^x^	73.5 (4)	Si1—Eu2—O3^xvi^	107.0 (2)
O1^ix^—Eu1—O1^x^	73.5 (4)	Si1—Eu2—O4	84.66 (16)
Ca1^xvii^—Ca2—Ca1^xviii^	104.79 (6)	Si1^xi^—Eu2—O2^xv^	127.4 (5)
Ca1^xvii^—Ca2—Ca1^xix^	83.96 (5)	Si1^xi^—Eu2—O3^xiii^	76.77 (18)
Ca1^xvii^—Ca2—Ca1^xx^	50.79 (11)	Si1^xi^—Eu2—O3^xvi^	76.77 (18)
Ca1^xvii^—Ca2—Eu1^xvii^	0	Si1^xi^—Eu2—O4	80.64 (19)
Ca1^xvii^—Ca2—Eu1^xviii^	104.79 (6)	O2^xv^—Eu2—O3^xiii^	85.1 (2)
Ca1^xvii^—Ca2—Eu1^xix^	83.96 (5)	O2^xv^—Eu2—O3^xvi^	85.1 (2)
Ca1^xvii^—Ca2—Eu1^xx^	50.79 (11)	O2^xv^—Eu2—O4	151.9 (4)
Ca1^xvii^—Ca2—Ca2^iv^	150.53 (7)	O3^xiii^—Eu2—O3^xvi^	137.4 (4)
Ca1^xvii^—Ca2—Ca2^xi^	103.10 (5)	O3^xiii^—Eu2—O4	103.97 (19)
Ca1^xvii^—Ca2—Ca2^xii^	60.59 (8)	O3^xvi^—Eu2—O4	103.97 (19)
Ca1^xvii^—Ca2—Ca2^xiii^	103.26 (10)	Ca1^xviii^—Si1—Ca1^xix^	65.07 (19)
Ca1^xvii^—Ca2—Ca2^viii^	96.58 (8)	Ca1^xviii^—Si1—Eu1^xviii^	0
Ca1^xvii^—Ca2—Ca2^xiv^	146.60 (9)	Ca1^xviii^—Si1—Eu1^xix^	65.07 (19)
Ca1^xvii^—Ca2—Eu2	0	Ca1^xviii^—Si1—Ca2	81.22 (19)
Ca1^xvii^—Ca2—Eu2^iv^	150.53 (7)	Ca1^xviii^—Si1—Ca2^iv^	139.42 (14)
Ca1^xvii^—Ca2—Eu2^xi^	103.10 (5)	Ca1^xviii^—Si1—Eu2	81.22 (19)
Ca1^xvii^—Ca2—Eu2^xii^	60.59 (8)	Ca1^xviii^—Si1—Eu2^iv^	139.42 (14)
Ca1^xvii^—Ca2—Eu2^xiii^	103.26 (10)	Ca1^xviii^—Si1—O1	137.0 (3)
Ca1^xvii^—Ca2—Eu2^viii^	96.58 (8)	Ca1^xviii^—Si1—O2	47.8 (4)
Ca1^xvii^—Ca2—Eu2^xiv^	146.60 (9)	Ca1^xviii^—Si1—O3	112.0 (5)
Ca1^xvii^—Ca2—Si1	134.45 (12)	Ca1^xviii^—Si1—O3^ii^	61.6 (3)
Ca1^xvii^—Ca2—Si1^xi^	57.83 (17)	Ca1^xix^—Si1—Eu1^xviii^	65.07 (19)
Ca1^xvii^—Ca2—O2^xv^	75.2 (4)	Ca1^xix^—Si1—Eu1^xix^	0
Ca1^xvii^—Ca2—O3^xiii^	94.1 (3)	Ca1^xix^—Si1—Ca2	81.22 (19)
Ca1^xvii^—Ca2—O3^xvi^	43.3 (3)	Ca1^xix^—Si1—Ca2^iv^	139.42 (14)
Ca1^xvii^—Ca2—O4	129.31 (6)	Ca1^xix^—Si1—Eu2	81.22 (19)
Ca1^xviii^—Ca2—Ca1^xix^	49.70 (10)	Ca1^xix^—Si1—Eu2^iv^	139.42 (14)
Ca1^xviii^—Ca2—Ca1^xx^	83.96 (5)	Ca1^xix^—Si1—O1	137.0 (3)
Ca1^xviii^—Ca2—Eu1^xvii^	104.79 (6)	Ca1^xix^—Si1—O2	47.8 (4)
Ca1^xviii^—Ca2—Eu1^xviii^	0	Ca1^xix^—Si1—O3	61.6 (3)
Ca1^xviii^—Ca2—Eu1^xix^	49.70 (10)	Ca1^xix^—Si1—O3^ii^	112.0 (5)
Ca1^xviii^—Ca2—Eu1^xx^	83.96 (5)	Eu1^xviii^—Si1—Eu1^xix^	65.07 (19)
Ca1^xviii^—Ca2—Ca2^iv^	99.26 (6)	Eu1^xviii^—Si1—Ca2	81.22 (19)
Ca1^xviii^—Ca2—Ca2^xi^	148.63 (8)	Eu1^xviii^—Si1—Ca2^iv^	139.42 (14)
Ca1^xviii^—Ca2—Ca2^xii^	146.90 (8)	Eu1^xviii^—Si1—Eu2	81.22 (19)
Ca1^xviii^—Ca2—Ca2^xiii^	97.57 (7)	Eu1^xviii^—Si1—Eu2^iv^	139.42 (14)
Ca1^xviii^—Ca2—Ca2^viii^	100.73 (8)	Eu1^xviii^—Si1—O1	137.0 (3)
Ca1^xviii^—Ca2—Ca2^xiv^	58.69 (7)	Eu1^xviii^—Si1—O2	47.8 (4)
Ca1^xviii^—Ca2—Eu2	0	Eu1^xviii^—Si1—O3	112.0 (5)
Ca1^xviii^—Ca2—Eu2^iv^	99.26 (6)	Eu1^xviii^—Si1—O3^ii^	61.6 (3)
Ca1^xviii^—Ca2—Eu2^xi^	148.63 (8)	Eu1^xix^—Si1—Ca2	81.22 (19)
Ca1^xviii^—Ca2—Eu2^xii^	146.90 (8)	Eu1^xix^—Si1—Ca2^iv^	139.42 (14)
Ca1^xviii^—Ca2—Eu2^xiii^	97.57 (7)	Eu1^xix^—Si1—Eu2	81.22 (19)
Ca1^xviii^—Ca2—Eu2^viii^	100.73 (8)	Eu1^xix^—Si1—Eu2^iv^	139.42 (14)
Ca1^xviii^—Ca2—Eu2^xiv^	58.69 (7)	Eu1^xix^—Si1—O1	137.0 (3)
Ca1^xviii^—Ca2—Si1	50.56 (12)	Eu1^xix^—Si1—O2	47.8 (4)
Ca1^xviii^—Ca2—Si1^xi^	141.16 (16)	Eu1^xix^—Si1—O3	61.6 (3)
Ca1^xviii^—Ca2—O2^xv^	32.5 (3)	Eu1^xix^—Si1—O3^ii^	112.0 (5)
Ca1^xviii^—Ca2—O3^xiii^	69.84 (15)	Ca2—Si1—Ca2^iv^	74.70 (13)
Ca1^xviii^—Ca2—O3^xvi^	116.00 (15)	Ca2—Si1—Eu2	0
Ca1^xviii^—Ca2—O4	125.87 (9)	Ca2—Si1—Eu2^iv^	74.70 (13)
Ca1^xix^—Ca2—Ca1^xx^	104.79 (6)	Ca2—Si1—O1	130.3 (4)
Ca1^xix^—Ca2—Eu1^xvii^	83.96 (5)	Ca2—Si1—O2	116.7 (6)
Ca1^xix^—Ca2—Eu1^xviii^	49.70 (10)	Ca2—Si1—O3	52.4 (3)
Ca1^xix^—Ca2—Eu1^xix^	0	Ca2—Si1—O3^ii^	52.4 (3)
Ca1^xix^—Ca2—Eu1^xx^	104.79 (6)	Ca2^iv^—Si1—Eu2	74.70 (13)
Ca1^xix^—Ca2—Ca2^iv^	99.26 (6)	Ca2^iv^—Si1—Eu2^iv^	0
Ca1^xix^—Ca2—Ca2^xi^	148.63 (8)	Ca2^iv^—Si1—O1	55.6 (3)
Ca1^xix^—Ca2—Ca2^xii^	97.57 (7)	Ca2^iv^—Si1—O2	168.6 (6)
Ca1^xix^—Ca2—Ca2^xiii^	146.90 (8)	Ca2^iv^—Si1—O3	77.8 (3)
Ca1^xix^—Ca2—Ca2^viii^	58.69 (7)	Ca2^iv^—Si1—O3^ii^	77.8 (3)
Ca1^xix^—Ca2—Ca2^xiv^	100.73 (8)	Eu2—Si1—Eu2^iv^	74.70 (13)
Ca1^xix^—Ca2—Eu2	0	Eu2—Si1—O1	130.3 (4)
Ca1^xix^—Ca2—Eu2^iv^	99.26 (6)	Eu2—Si1—O2	116.7 (6)
Ca1^xix^—Ca2—Eu2^xi^	148.63 (8)	Eu2—Si1—O3	52.4 (3)
Ca1^xix^—Ca2—Eu2^xii^	97.57 (7)	Eu2—Si1—O3^ii^	52.4 (3)
Ca1^xix^—Ca2—Eu2^xiii^	146.90 (8)	Eu2^iv^—Si1—O1	55.6 (3)
Ca1^xix^—Ca2—Eu2^viii^	58.69 (7)	Eu2^iv^—Si1—O2	168.6 (6)
Ca1^xix^—Ca2—Eu2^xiv^	100.73 (8)	Eu2^iv^—Si1—O3	77.8 (3)
Ca1^xix^—Ca2—Si1	50.56 (12)	Eu2^iv^—Si1—O3^ii^	77.8 (3)
Ca1^xix^—Ca2—Si1^xi^	141.16 (16)	O1—Si1—O2	113.0 (7)
Ca1^xix^—Ca2—O2^xv^	32.5 (3)	O1—Si1—O3	110.8 (5)
Ca1^xix^—Ca2—O3^xiii^	116.00 (15)	O1—Si1—O3^ii^	110.8 (5)
Ca1^xix^—Ca2—O3^xvi^	69.84 (15)	O2—Si1—O3	108.7 (5)
Ca1^xix^—Ca2—O4	125.87 (9)	O2—Si1—O3^ii^	108.7 (5)
Ca1^xx^—Ca2—Eu1^xvii^	50.79 (11)	O3—Si1—O3^ii^	104.5 (5)
Ca1^xx^—Ca2—Eu1^xviii^	83.96 (5)	Ca1—O1—Ca1^ii^	92.6 (4)
Ca1^xx^—Ca2—Eu1^xix^	104.79 (6)	Ca1—O1—Eu1	0
Ca1^xx^—Ca2—Eu1^xx^	0	Ca1—O1—Eu1^ii^	92.6 (4)
Ca1^xx^—Ca2—Ca2^iv^	150.53 (7)	Ca1—O1—Si1	128.2 (3)
Ca1^xx^—Ca2—Ca2^xi^	103.10 (5)	Ca1^ii^—O1—Eu1	92.6 (4)
Ca1^xx^—Ca2—Ca2^xii^	103.26 (10)	Ca1^ii^—O1—Eu1^ii^	0
Ca1^xx^—Ca2—Ca2^xiii^	60.59 (8)	Ca1^ii^—O1—Si1	128.2 (3)
Ca1^xx^—Ca2—Ca2^viii^	146.60 (9)	Eu1—O1—Eu1^ii^	92.6 (4)
Ca1^xx^—Ca2—Ca2^xiv^	96.58 (8)	Eu1—O1—Si1	128.2 (3)
Ca1^xx^—Ca2—Eu2	0	Eu1^ii^—O1—Si1	128.2 (3)
Ca1^xx^—Ca2—Eu2^iv^	150.53 (7)	Ca1^xviii^—O2—Ca1^xix^	90.0 (5)
Ca1^xx^—Ca2—Eu2^xi^	103.10 (5)	Ca1^xviii^—O2—Ca2^v^	115.2 (3)
Ca1^xx^—Ca2—Eu2^xii^	103.26 (10)	Ca1^xviii^—O2—Eu2^v^	115.2 (3)
Ca1^xx^—Ca2—Eu2^xiii^	60.59 (8)	Ca1^xviii^—O2—Si1	103.3 (4)
Ca1^xx^—Ca2—Eu2^viii^	146.60 (9)	Ca1^xix^—O2—Ca2^v^	115.2 (3)
Ca1^xx^—Ca2—Eu2^xiv^	96.58 (8)	Ca1^xix^—O2—Eu2^v^	115.2 (3)
Ca1^xx^—Ca2—Si1	134.45 (12)	Ca1^xix^—O2—Si1	103.3 (4)
Ca1^xx^—Ca2—Si1^xi^	57.83 (17)	Ca2^v^—O2—Eu2^v^	0
Ca1^xx^—Ca2—O2^xv^	75.2 (4)	Ca2^v^—O2—Si1	124.0 (8)
Ca1^xx^—Ca2—O3^xiii^	43.3 (3)	Eu2^v^—O2—Si1	124.0 (8)
Ca1^xx^—Ca2—O3^xvi^	94.1 (3)	Ca2^viii^—O3—Eu2^viii^	0
Ca1^xx^—Ca2—O4	129.31 (6)	Ca2^viii^—O3—Si1	141.2 (5)
Eu1^xvii^—Ca2—Eu1^xviii^	104.79 (6)	Eu2^viii^—O3—Si1	141.2 (5)
Eu1^xvii^—Ca2—Eu1^xix^	83.96 (5)	Ca2—O4—Ca2^iv^	120.00 (9)
Eu1^xvii^—Ca2—Eu1^xx^	50.79 (11)	Ca2—O4—Ca2^xi^	120.00 (10)
Eu1^xvii^—Ca2—Ca2^iv^	150.53 (7)	Ca2—O4—Eu2	0
Eu1^xvii^—Ca2—Ca2^xi^	103.10 (5)	Ca2—O4—Eu2^iv^	120.00 (9)
Eu1^xvii^—Ca2—Ca2^xii^	60.59 (8)	Ca2—O4—Eu2^xi^	120.00 (10)
Eu1^xvii^—Ca2—Ca2^xiii^	103.26 (10)	Ca2^iv^—O4—Ca2^xi^	120.00 (10)
Eu1^xvii^—Ca2—Ca2^viii^	96.58 (8)	Ca2^iv^—O4—Eu2	120.00 (9)
Eu1^xvii^—Ca2—Ca2^xiv^	146.60 (9)	Ca2^iv^—O4—Eu2^iv^	0
Eu1^xvii^—Ca2—Eu2	0	Ca2^iv^—O4—Eu2^xi^	120.00 (10)
Eu1^xvii^—Ca2—Eu2^iv^	150.53 (7)	Ca2^xi^—O4—Eu2	120.00 (10)
Eu1^xvii^—Ca2—Eu2^xi^	103.10 (5)	Ca2^xi^—O4—Eu2^iv^	120.00 (10)
Eu1^xvii^—Ca2—Eu2^xii^	60.59 (8)	Ca2^xi^—O4—Eu2^xi^	0
Eu1^xvii^—Ca2—Eu2^xiii^	103.26 (10)	Eu2—O4—Eu2^iv^	120.00 (9)
Eu1^xvii^—Ca2—Eu2^viii^	96.58 (8)	Eu2—O4—Eu2^xi^	120.00 (10)
Eu1^xvii^—Ca2—Eu2^xiv^	146.60 (9)	Eu2^iv^—O4—Eu2^xi^	120.00 (10)

Symmetry codes: (i) *x*, *y*, −*z*−1/2; (ii) *x*, *y*, −*z*+1/2; (iii) *x*, *y*+1, *z*; (iv) −*y*, *x*−*y*, *z*; (v) −*x*+*y*+1, −*x*+1, *z*; (vi) −*x*+1, −*y*+1, *z*−1/2; (vii) *y*, −*x*+*y*+1, *z*−1/2; (viii) *x*−*y*, *x*, *z*−1/2; (ix) −*y*+1, *x*−*y*+1, *z*; (x) −*x*+*y*, −*x*+1, *z*; (xi) −*x*+*y*, −*x*, *z*; (xii) *y*, −*x*+*y*, *z*−1/2; (xiii) *y*, −*x*+*y*, *z*+1/2; (xiv) *x*−*y*, *x*, *z*+1/2; (xv) −*y*+1, *x*−*y*, *z*; (xvi) *y*, −*x*+*y*, −*z*; (xvii) *x*, *y*−1, *z*; (xviii) −*x*+1, −*y*+1, *z*+1/2; (xix) −*x*+1, −*y*+1, −*z*; (xx) *x*, *y*−1, −*z*+1/2.

### Calcium ytterbium silicate oxyapatite (Ca-Yb) Crystal data

**Table:**

Ca_2_Yb_8_(SiO_4_)_6_O_2_	*Z* = 1
*M**_r_* = 2048.7	*D*_x_ = 6.785 Mg m^−^^3^
Hexagonal, *P*6_3_/*m*	Cu *K*α radiation, λ = 1.54188 Å
*a* = 9.29743 (7) Å	*T* = 295 K
*c* = 6.69748 (6) Å	white
*V* = 501.38 (1) Å^3^	flat_sheet, 25 × 25 mm

### Calcium ytterbium silicate oxyapatite (Ca-Yb) Data collection

**Table:**

Bruker D8 Advance diffractometer	Data collection mode: reflection
Radiation source: sealed X-ray tube	Scan method: step
Specimen mounting: packed powder pellet	2θ_min_ = 10°, 2θ_max_ = 70°, 2θ_step_ = 0.009°

### Calcium ytterbium silicate oxyapatite (Ca-Yb) Refinement

**Table:**

*R*_p_ = 0.05	36 parameters
*R*_wp_ = 0.07	Weighting scheme based on measured s.u.'s
*R*_exp_ = 0.02	(Δ/σ)_max_ = 0.039
*R*_Bragg_ = 0.04	Background function: Chebychev
6994 data points	Preferred orientation correction: spherical harmonic
Profile function: pseudo-Voigt	

### Calcium ytterbium silicate oxyapatite (Ca-Yb) Special details

**Table:**

Refinement. Beq were fixed as 1Å squared during refinement as they result high errors

### Calcium ytterbium silicate oxyapatite (Ca-Yb) Fractional atomic coordinates and isotropic or equivalent isotropic displacement parameters (Å^2^)

**Table:**

	*x*	*y*	*z*	*U*_iso_*/*U*_eq_	Occ. (<1)
Ca1	0.333333	0.666667	−0.0006 (8)	0.0127*	0.448
Yb1	0.333333	0.666667	−0.0006 (8)	0.0127*	0.552
Ca2	0.23445 (17)	−0.0010 (3)	0.25	0.0127*	0.035
Yb2	0.23445 (17)	−0.0010 (3)	0.25	0.0127*	0.965
Si1	0.3751 (9)	0.3955 (9)	0.25	0.0127*	
O1	0.1336 (14)	0.5361 (13)	0.25	0.0127*	
O2	0.8349 (13)	0.5129 (13)	0.25	0.0127*	
O3	0.2557 (8)	0.3390 (7)	0.0659 (10)	0.0127*	
O4	0	0	0.25	0.0127*	

### Calcium ytterbium silicate oxyapatite (Ca-Yb) Atomic displacement parameters (Å^2^)

**Table:**

	*U*^11^	*U*^22^	*U*^33^	*U*^12^	*U*^13^	*U*^23^
?	?	?	?	?	?	?

### Calcium ytterbium silicate oxyapatite (Ca-Yb) Geometric parameters (Å, º)

**Table:**

Ca1—Ca1^i^	3.341 (8)	Yb1—Si1^ix^	3.210 (7)
Ca1—Ca1^ii^	3.357 (8)	Yb1—Si1^x^	3.210 (11)
Ca1—Yb1	0	Yb1—O2^vi^	2.326 (7)
Ca1—Yb1^i^	3.341 (8)	Yb1—O2^vii^	2.326 (11)
Ca1—Yb1^ii^	3.357 (8)	Yb1—O2^viii^	2.326 (11)
Ca1—Ca2^iii^	4.006 (3)	Ca2—Ca2^iv^	3.784 (5)
Ca1—Ca2^iv^	4.006 (3)	Ca2—Ca2^xi^	3.784 (3)
Ca1—Ca2^v^	4.006 (4)	Ca2—Ca2^xii^	3.9982 (15)
Ca1—Ca2^vi^	4.014 (3)	Ca2—Ca2^xiii^	3.9982 (15)
Ca1—Ca2^vii^	4.014 (4)	Ca2—Ca2^viii^	3.998 (2)
Ca1—Ca2^viii^	4.014 (3)	Ca2—Ca2^xiv^	3.998 (2)
Ca1—Yb2^iii^	4.006 (3)	Ca2—Yb2	0
Ca1—Yb2^iv^	4.006 (3)	Ca2—Yb2^iv^	3.784 (5)
Ca1—Yb2^v^	4.006 (4)	Ca2—Yb2^xi^	3.784 (3)
Ca1—Yb2^vi^	4.014 (3)	Ca2—Yb2^xii^	3.9982 (15)
Ca1—Yb2^vii^	4.014 (4)	Ca2—Yb2^xiii^	3.9982 (15)
Ca1—Yb2^viii^	4.014 (3)	Ca2—Yb2^viii^	3.998 (2)
Ca1—Si1	3.210 (9)	Ca2—Yb2^xiv^	3.998 (2)
Ca1—Si1^ix^	3.210 (7)	Ca2—Si1	3.237 (8)
Ca1—Si1^x^	3.210 (11)	Ca2—Si1^xi^	3.023 (7)
Ca1—O1	2.342 (8)	Ca2—O1^xi^	2.426 (19)
Ca1—O1^ix^	2.342 (13)	Ca2—O3^xi^	2.403 (7)
Ca1—O1^x^	2.342 (10)	Ca2—O3^xiii^	2.296 (7)
Ca1—O2^vi^	2.326 (7)	Ca2—O3^xv^	2.296 (7)
Ca1—O2^vii^	2.326 (11)	Ca2—O3^xvi^	2.403 (7)
Ca1—O2^viii^	2.326 (11)	Ca2—O4	2.184 (2)
Yb1—Yb1^i^	3.341 (8)	Yb2—Yb2^iv^	3.784 (5)
Yb1—Yb1^ii^	3.357 (8)	Yb2—Yb2^xi^	3.784 (3)
Yb1—Ca2^iii^	4.006 (3)	Yb2—Yb2^xii^	3.9982 (15)
Yb1—Ca2^iv^	4.006 (3)	Yb2—Yb2^xiii^	3.9982 (15)
Yb1—Ca2^v^	4.006 (4)	Yb2—Yb2^viii^	3.998 (2)
Yb1—Ca2^vi^	4.014 (3)	Yb2—Yb2^xiv^	3.998 (2)
Yb1—Ca2^vii^	4.014 (4)	Yb2—Si1	3.237 (8)
Yb1—Ca2^viii^	4.014 (3)	Yb2—Si1^xi^	3.023 (7)
Yb1—Yb2^iii^	4.006 (3)	Yb2—O3^xiii^	2.296 (7)
Yb1—Yb2^iv^	4.006 (3)	Yb2—O3^xv^	2.296 (7)
Yb1—Yb2^v^	4.006 (4)	Yb2—O4	2.184 (2)
Yb1—Yb2^vi^	4.014 (3)	Si1—O1^ix^	1.630 (18)
Yb1—Yb2^vii^	4.014 (4)	Si1—O2^xvii^	1.50 (2)
Yb1—Yb2^viii^	4.014 (3)	Si1—O3	1.564 (8)
Yb1—Si1	3.210 (9)	Si1—O3^ii^	1.564 (8)
			
Ca1^i^—Ca1—Ca1^ii^	180	Yb1^xviii^—Ca2—O3^xvi^	72.6 (2)
Ca1^i^—Ca1—Yb1	0	Yb1^xviii^—Ca2—O4	128.14 (6)
Ca1^i^—Ca1—Yb1^i^	0	Yb1^xix^—Ca2—Yb1^xx^	49.19 (10)
Ca1^i^—Ca1—Yb1^ii^	180	Yb1^xix^—Ca2—Yb1^xxi^	84.03 (5)
Ca1^i^—Ca1—Ca2^iii^	114.77 (7)	Yb1^xix^—Ca2—Ca2^iv^	101.13 (6)
Ca1^i^—Ca1—Ca2^iv^	114.77 (7)	Yb1^xix^—Ca2—Ca2^xi^	150.00 (8)
Ca1^i^—Ca1—Ca2^v^	114.77 (7)	Yb1^xix^—Ca2—Ca2^xii^	146.51 (8)
Ca1^i^—Ca1—Ca2^vi^	65.41 (7)	Yb1^xix^—Ca2—Ca2^xiii^	97.87 (7)
Ca1^i^—Ca1—Ca2^vii^	65.41 (7)	Yb1^xix^—Ca2—Ca2^viii^	101.37 (8)
Ca1^i^—Ca1—Ca2^viii^	65.41 (7)	Yb1^xix^—Ca2—Ca2^xiv^	60.00 (7)
Ca1^i^—Ca1—Yb2^iii^	114.77 (7)	Yb1^xix^—Ca2—Yb2	0
Ca1^i^—Ca1—Yb2^iv^	114.77 (7)	Yb1^xix^—Ca2—Yb2^iv^	101.13 (6)
Ca1^i^—Ca1—Yb2^v^	114.77 (7)	Yb1^xix^—Ca2—Yb2^xi^	150.00 (8)
Ca1^i^—Ca1—Yb2^vi^	65.41 (7)	Yb1^xix^—Ca2—Yb2^xii^	146.51 (8)
Ca1^i^—Ca1—Yb2^vii^	65.41 (7)	Yb1^xix^—Ca2—Yb2^xiii^	97.87 (7)
Ca1^i^—Ca1—Yb2^viii^	65.41 (7)	Yb1^xix^—Ca2—Yb2^viii^	101.37 (8)
Ca1^i^—Ca1—Si1	121.53 (12)	Yb1^xix^—Ca2—Yb2^xiv^	60.00 (7)
Ca1^i^—Ca1—Si1^ix^	121.53 (11)	Yb1^xix^—Ca2—Si1	55.95 (14)
Ca1^i^—Ca1—Si1^x^	121.53 (14)	Yb1^xix^—Ca2—Si1^xi^	136.0 (2)
Ca1^i^—Ca1—O1	135.8 (2)	Yb1^xix^—Ca2—O1^xi^	75.2 (2)
Ca1^i^—Ca1—O1^ix^	135.8 (3)	Yb1^xix^—Ca2—O3^xi^	147.4 (3)
Ca1^i^—Ca1—O1^x^	135.8 (2)	Yb1^xix^—Ca2—O3^xiii^	42.54 (16)
Ca1^i^—Ca1—O2^vi^	44.11 (18)	Yb1^xix^—Ca2—O3^xv^	91.72 (16)
Ca1^i^—Ca1—O2^vii^	44.1 (3)	Yb1^xix^—Ca2—O3^xvi^	114.5 (2)
Ca1^i^—Ca1—O2^viii^	44.1 (3)	Yb1^xix^—Ca2—O4	127.70 (9)
Ca1^ii^—Ca1—Yb1	0	Yb1^xx^—Ca2—Yb1^xxi^	104.16 (6)
Ca1^ii^—Ca1—Yb1^i^	180	Yb1^xx^—Ca2—Ca2^iv^	101.13 (6)
Ca1^ii^—Ca1—Yb1^ii^	0	Yb1^xx^—Ca2—Ca2^xi^	150.00 (8)
Ca1^ii^—Ca1—Ca2^iii^	65.23 (7)	Yb1^xx^—Ca2—Ca2^xii^	97.87 (7)
Ca1^ii^—Ca1—Ca2^iv^	65.23 (7)	Yb1^xx^—Ca2—Ca2^xiii^	146.51 (8)
Ca1^ii^—Ca1—Ca2^v^	65.23 (7)	Yb1^xx^—Ca2—Ca2^viii^	60.00 (7)
Ca1^ii^—Ca1—Ca2^vi^	114.59 (7)	Yb1^xx^—Ca2—Ca2^xiv^	101.37 (8)
Ca1^ii^—Ca1—Ca2^vii^	114.59 (7)	Yb1^xx^—Ca2—Yb2	0
Ca1^ii^—Ca1—Ca2^viii^	114.59 (7)	Yb1^xx^—Ca2—Yb2^iv^	101.13 (6)
Ca1^ii^—Ca1—Yb2^iii^	65.23 (7)	Yb1^xx^—Ca2—Yb2^xi^	150.00 (8)
Ca1^ii^—Ca1—Yb2^iv^	65.23 (7)	Yb1^xx^—Ca2—Yb2^xii^	97.87 (7)
Ca1^ii^—Ca1—Yb2^v^	65.23 (7)	Yb1^xx^—Ca2—Yb2^xiii^	146.51 (8)
Ca1^ii^—Ca1—Yb2^vi^	114.59 (7)	Yb1^xx^—Ca2—Yb2^viii^	60.00 (7)
Ca1^ii^—Ca1—Yb2^vii^	114.59 (7)	Yb1^xx^—Ca2—Yb2^xiv^	101.37 (8)
Ca1^ii^—Ca1—Yb2^viii^	114.59 (7)	Yb1^xx^—Ca2—Si1	55.95 (14)
Ca1^ii^—Ca1—Si1	58.47 (12)	Yb1^xx^—Ca2—Si1^xi^	136.0 (2)
Ca1^ii^—Ca1—Si1^ix^	58.47 (11)	Yb1^xx^—Ca2—O1^xi^	75.2 (2)
Ca1^ii^—Ca1—Si1^x^	58.47 (14)	Yb1^xx^—Ca2—O3^xi^	114.5 (2)
Ca1^ii^—Ca1—O1	44.2 (2)	Yb1^xx^—Ca2—O3^xiii^	91.72 (16)
Ca1^ii^—Ca1—O1^ix^	44.2 (3)	Yb1^xx^—Ca2—O3^xv^	42.54 (16)
Ca1^ii^—Ca1—O1^x^	44.2 (2)	Yb1^xx^—Ca2—O3^xvi^	147.4 (3)
Ca1^ii^—Ca1—O2^vi^	135.89 (18)	Yb1^xx^—Ca2—O4	127.70 (9)
Ca1^ii^—Ca1—O2^vii^	135.9 (3)	Yb1^xxi^—Ca2—Ca2^iv^	150.19 (6)
Ca1^ii^—Ca1—O2^viii^	135.9 (3)	Yb1^xxi^—Ca2—Ca2^xi^	101.65 (6)
Yb1—Ca1—Yb1^i^	0	Yb1^xxi^—Ca2—Ca2^xii^	101.81 (10)
Yb1—Ca1—Yb1^ii^	0	Yb1^xxi^—Ca2—Ca2^xiii^	60.19 (8)
Yb1—Ca1—Ca2^iii^	0	Yb1^xxi^—Ca2—Ca2^viii^	146.57 (9)
Yb1—Ca1—Ca2^iv^	0	Yb1^xxi^—Ca2—Ca2^xiv^	97.63 (8)
Yb1—Ca1—Ca2^v^	0	Yb1^xxi^—Ca2—Yb2	0
Yb1—Ca1—Ca2^vi^	0	Yb1^xxi^—Ca2—Yb2^iv^	150.19 (6)
Yb1—Ca1—Ca2^vii^	0	Yb1^xxi^—Ca2—Yb2^xi^	101.65 (6)
Yb1—Ca1—Ca2^viii^	0	Yb1^xxi^—Ca2—Yb2^xii^	101.81 (10)
Yb1—Ca1—Yb2^iii^	0	Yb1^xxi^—Ca2—Yb2^xiii^	60.19 (8)
Yb1—Ca1—Yb2^iv^	0	Yb1^xxi^—Ca2—Yb2^viii^	146.57 (9)
Yb1—Ca1—Yb2^v^	0	Yb1^xxi^—Ca2—Yb2^xiv^	97.63 (8)
Yb1—Ca1—Yb2^vi^	0	Yb1^xxi^—Ca2—Si1	139.51 (13)
Yb1—Ca1—Yb2^vii^	0	Yb1^xxi^—Ca2—Si1^xi^	52.1 (2)
Yb1—Ca1—Yb2^viii^	0	Yb1^xxi^—Ca2—O1^xi^	32.19 (17)
Yb1—Ca1—Si1	0	Yb1^xxi^—Ca2—O3^xi^	72.6 (2)
Yb1—Ca1—Si1^ix^	0	Yb1^xxi^—Ca2—O3^xiii^	69.9 (3)
Yb1—Ca1—Si1^x^	0	Yb1^xxi^—Ca2—O3^xv^	115.3 (3)
Yb1—Ca1—O1	0	Yb1^xxi^—Ca2—O3^xvi^	43.3 (3)
Yb1—Ca1—O1^ix^	0	Yb1^xxi^—Ca2—O4	128.14 (6)
Yb1—Ca1—O1^x^	0	Ca2^iv^—Ca2—Ca2^xi^	60.00 (6)
Yb1—Ca1—O2^vi^	0	Ca2^iv^—Ca2—Ca2^xii^	90.00 (7)
Yb1—Ca1—O2^vii^	0	Ca2^iv^—Ca2—Ca2^xiii^	90.00 (7)
Yb1—Ca1—O2^viii^	0	Ca2^iv^—Ca2—Ca2^viii^	61.76 (6)
Yb1^i^—Ca1—Yb1^ii^	180	Ca2^iv^—Ca2—Ca2^xiv^	61.76 (6)
Yb1^i^—Ca1—Ca2^iii^	114.77 (7)	Ca2^iv^—Ca2—Yb2	0
Yb1^i^—Ca1—Ca2^iv^	114.77 (7)	Ca2^iv^—Ca2—Yb2^iv^	0
Yb1^i^—Ca1—Ca2^v^	114.77 (7)	Ca2^iv^—Ca2—Yb2^xi^	60.00 (6)
Yb1^i^—Ca1—Ca2^vi^	65.41 (7)	Ca2^iv^—Ca2—Yb2^xii^	90.00 (7)
Yb1^i^—Ca1—Ca2^vii^	65.41 (7)	Ca2^iv^—Ca2—Yb2^xiii^	90.00 (7)
Yb1^i^—Ca1—Ca2^viii^	65.41 (7)	Ca2^iv^—Ca2—Yb2^viii^	61.76 (6)
Yb1^i^—Ca1—Yb2^iii^	114.77 (7)	Ca2^iv^—Ca2—Yb2^xiv^	61.76 (6)
Yb1^i^—Ca1—Yb2^iv^	114.77 (7)	Ca2^iv^—Ca2—Si1	50.27 (16)
Yb1^i^—Ca1—Yb2^v^	114.77 (7)	Ca2^iv^—Ca2—Si1^xi^	115.4 (2)
Yb1^i^—Ca1—Yb2^vi^	65.41 (7)	Ca2^iv^—Ca2—O1^xi^	175.9 (3)
Yb1^i^—Ca1—Yb2^vii^	65.41 (7)	Ca2^iv^—Ca2—O3^xi^	110.5 (3)
Yb1^i^—Ca1—Yb2^viii^	65.41 (7)	Ca2^iv^—Ca2—O3^xiii^	94.0 (3)
Yb1^i^—Ca1—Si1	121.53 (12)	Ca2^iv^—Ca2—O3^xv^	94.0 (3)
Yb1^i^—Ca1—Si1^ix^	121.53 (11)	Ca2^iv^—Ca2—O3^xvi^	110.5 (3)
Yb1^i^—Ca1—Si1^x^	121.53 (14)	Ca2^iv^—Ca2—O4	30.00 (5)
Yb1^i^—Ca1—O1	135.8 (2)	Ca2^xi^—Ca2—Ca2^xii^	61.76 (4)
Yb1^i^—Ca1—O1^ix^	135.8 (3)	Ca2^xi^—Ca2—Ca2^xiii^	61.76 (4)
Yb1^i^—Ca1—O1^x^	135.8 (2)	Ca2^xi^—Ca2—Ca2^viii^	90.00 (6)
Yb1^i^—Ca1—O2^vi^	44.11 (18)	Ca2^xi^—Ca2—Ca2^xiv^	90.00 (6)
Yb1^i^—Ca1—O2^vii^	44.1 (3)	Ca2^xi^—Ca2—Yb2	0
Yb1^i^—Ca1—O2^viii^	44.1 (3)	Ca2^xi^—Ca2—Yb2^iv^	60.00 (6)
Yb1^ii^—Ca1—Ca2^iii^	65.23 (7)	Ca2^xi^—Ca2—Yb2^xi^	0
Yb1^ii^—Ca1—Ca2^iv^	65.23 (7)	Ca2^xi^—Ca2—Yb2^xii^	61.76 (4)
Yb1^ii^—Ca1—Ca2^v^	65.23 (7)	Ca2^xi^—Ca2—Yb2^xiii^	61.76 (4)
Yb1^ii^—Ca1—Ca2^vi^	114.59 (7)	Ca2^xi^—Ca2—Yb2^viii^	90.00 (6)
Yb1^ii^—Ca1—Ca2^vii^	114.59 (7)	Ca2^xi^—Ca2—Yb2^xiv^	90.00 (6)
Yb1^ii^—Ca1—Ca2^viii^	114.59 (7)	Ca2^xi^—Ca2—Si1	110.27 (17)
Yb1^ii^—Ca1—Yb2^iii^	65.23 (7)	Ca2^xi^—Ca2—Si1^xi^	55.4 (2)
Yb1^ii^—Ca1—Yb2^iv^	65.23 (7)	Ca2^xi^—Ca2—O1^xi^	124.1 (3)
Yb1^ii^—Ca1—Yb2^v^	65.23 (7)	Ca2^xi^—Ca2—O3^xi^	59.7 (2)
Yb1^ii^—Ca1—Yb2^vi^	114.59 (7)	Ca2^xi^—Ca2—O3^xiii^	111.47 (15)
Yb1^ii^—Ca1—Yb2^vii^	114.59 (7)	Ca2^xi^—Ca2—O3^xv^	111.47 (15)
Yb1^ii^—Ca1—Yb2^viii^	114.59 (7)	Ca2^xi^—Ca2—O3^xvi^	59.7 (2)
Yb1^ii^—Ca1—Si1	58.47 (12)	Ca2^xi^—Ca2—O4	30.00 (4)
Yb1^ii^—Ca1—Si1^ix^	58.47 (11)	Ca2^xii^—Ca2—Ca2^xiii^	113.77 (5)
Yb1^ii^—Ca1—Si1^x^	58.47 (14)	Ca2^xii^—Ca2—Ca2^viii^	56.48 (4)
Yb1^ii^—Ca1—O1	44.2 (2)	Ca2^xii^—Ca2—Ca2^xiv^	148.29 (9)
Yb1^ii^—Ca1—O1^ix^	44.2 (3)	Ca2^xii^—Ca2—Yb2	0
Yb1^ii^—Ca1—O1^x^	44.2 (2)	Ca2^xii^—Ca2—Yb2^iv^	90.00 (7)
Yb1^ii^—Ca1—O2^vi^	135.89 (18)	Ca2^xii^—Ca2—Yb2^xi^	61.76 (4)
Yb1^ii^—Ca1—O2^vii^	135.9 (3)	Ca2^xii^—Ca2—Yb2^xii^	0
Yb1^ii^—Ca1—O2^viii^	135.9 (3)	Ca2^xii^—Ca2—Yb2^xiii^	113.77 (5)
Ca2^iii^—Ca1—Ca2^iv^	103.69 (10)	Ca2^xii^—Ca2—Yb2^viii^	56.48 (4)
Ca2^iii^—Ca1—Ca2^v^	103.69 (9)	Ca2^xii^—Ca2—Yb2^xiv^	148.29 (9)
Ca2^iii^—Ca1—Ca2^vi^	95.97 (4)	Ca2^xii^—Ca2—Si1	114.84 (9)
Ca2^iii^—Ca1—Ca2^vii^	59.81 (4)	Ca2^xii^—Ca2—Si1^xi^	60.44 (8)
Ca2^iii^—Ca1—Ca2^viii^	157.21 (5)	Ca2^xii^—Ca2—O1^xi^	92.24 (16)
Ca2^iii^—Ca1—Yb2^iii^	0	Ca2^xii^—Ca2—O3^xi^	30.91 (19)
Ca2^iii^—Ca1—Yb2^iv^	103.69 (10)	Ca2^xii^—Ca2—O3^xiii^	168.7 (2)
Ca2^iii^—Ca1—Yb2^v^	103.69 (9)	Ca2^xii^—Ca2—O3^xv^	55.76 (15)
Ca2^iii^—Ca1—Yb2^vi^	95.97 (4)	Ca2^xii^—Ca2—O3^xvi^	90.09 (17)
Ca2^iii^—Ca1—Yb2^vii^	59.81 (4)	Ca2^xii^—Ca2—O4	74.15 (5)
Ca2^iii^—Ca1—Yb2^viii^	157.21 (5)	Ca2^xiii^—Ca2—Ca2^viii^	148.29 (9)
Ca2^iii^—Ca1—Si1	123.45 (18)	Ca2^xiii^—Ca2—Ca2^xiv^	56.48 (4)
Ca2^iii^—Ca1—Si1^ix^	57.4 (2)	Ca2^xiii^—Ca2—Yb2	0
Ca2^iii^—Ca1—Si1^x^	47.98 (14)	Ca2^xiii^—Ca2—Yb2^iv^	90.00 (7)
Ca2^iii^—Ca1—O1	70.8 (3)	Ca2^xiii^—Ca2—Yb2^xi^	61.76 (4)
Ca2^iii^—Ca1—O1^ix^	105.2 (4)	Ca2^xiii^—Ca2—Yb2^xii^	113.77 (5)
Ca2^iii^—Ca1—O1^x^	33.5 (4)	Ca2^xiii^—Ca2—Yb2^xiii^	0
Ca2^iii^—Ca1—O2^vi^	121.5 (4)	Ca2^xiii^—Ca2—Yb2^viii^	148.29 (9)
Ca2^iii^—Ca1—O2^vii^	134.6 (4)	Ca2^xiii^—Ca2—Yb2^xiv^	56.48 (4)
Ca2^iii^—Ca1—O2^viii^	71.2 (3)	Ca2^xiii^—Ca2—Si1	114.84 (9)
Ca2^iv^—Ca1—Ca2^v^	103.69 (9)	Ca2^xiii^—Ca2—Si1^xi^	60.44 (8)
Ca2^iv^—Ca1—Ca2^vi^	157.21 (7)	Ca2^xiii^—Ca2—O1^xi^	92.24 (16)
Ca2^iv^—Ca1—Ca2^vii^	95.97 (5)	Ca2^xiii^—Ca2—O3^xi^	90.09 (17)
Ca2^iv^—Ca1—Ca2^viii^	59.81 (4)	Ca2^xiii^—Ca2—O3^xiii^	55.76 (15)
Ca2^iv^—Ca1—Yb2^iii^	103.69 (10)	Ca2^xiii^—Ca2—O3^xv^	168.7 (2)
Ca2^iv^—Ca1—Yb2^iv^	0	Ca2^xiii^—Ca2—O3^xvi^	30.91 (19)
Ca2^iv^—Ca1—Yb2^v^	103.69 (9)	Ca2^xiii^—Ca2—O4	74.15 (5)
Ca2^iv^—Ca1—Yb2^vi^	157.21 (7)	Ca2^viii^—Ca2—Ca2^xiv^	113.77 (8)
Ca2^iv^—Ca1—Yb2^vii^	95.97 (5)	Ca2^viii^—Ca2—Yb2	0
Ca2^iv^—Ca1—Yb2^viii^	59.81 (4)	Ca2^viii^—Ca2—Yb2^iv^	61.76 (6)
Ca2^iv^—Ca1—Si1	47.98 (14)	Ca2^viii^—Ca2—Yb2^xi^	90.00 (6)
Ca2^iv^—Ca1—Si1^ix^	123.45 (17)	Ca2^viii^—Ca2—Yb2^xii^	56.48 (4)
Ca2^iv^—Ca1—Si1^x^	57.38 (16)	Ca2^viii^—Ca2—Yb2^xiii^	148.29 (9)
Ca2^iv^—Ca1—O1	33.5 (3)	Ca2^viii^—Ca2—Yb2^viii^	0
Ca2^iv^—Ca1—O1^ix^	70.8 (3)	Ca2^viii^—Ca2—Yb2^xiv^	113.77 (8)
Ca2^iv^—Ca1—O1^x^	105.2 (3)	Ca2^viii^—Ca2—Si1	59.17 (5)
Ca2^iv^—Ca1—O2^vi^	71.17 (17)	Ca2^viii^—Ca2—Si1^xi^	116.74 (9)
Ca2^iv^—Ca1—O2^vii^	121.5 (4)	Ca2^viii^—Ca2—O1^xi^	116.90 (10)
Ca2^iv^—Ca1—O2^viii^	134.6 (3)	Ca2^viii^—Ca2—O3^xi^	87.13 (18)
Ca2^v^—Ca1—Ca2^vi^	59.81 (3)	Ca2^viii^—Ca2—O3^xiii^	134.4 (2)
Ca2^v^—Ca1—Ca2^vii^	157.21 (4)	Ca2^viii^—Ca2—O3^xv^	32.5 (3)
Ca2^v^—Ca1—Ca2^viii^	95.97 (5)	Ca2^viii^—Ca2—O3^xvi^	144.04 (19)
Ca2^v^—Ca1—Yb2^iii^	103.69 (9)	Ca2^viii^—Ca2—O4	74.15 (7)
Ca2^v^—Ca1—Yb2^iv^	103.69 (9)	Ca2^xiv^—Ca2—Yb2	0
Ca2^v^—Ca1—Yb2^v^	0	Ca2^xiv^—Ca2—Yb2^iv^	61.76 (6)
Ca2^v^—Ca1—Yb2^vi^	59.81 (3)	Ca2^xiv^—Ca2—Yb2^xi^	90.00 (6)
Ca2^v^—Ca1—Yb2^vii^	157.21 (4)	Ca2^xiv^—Ca2—Yb2^xii^	148.29 (9)
Ca2^v^—Ca1—Yb2^viii^	95.97 (5)	Ca2^xiv^—Ca2—Yb2^xiii^	56.48 (4)
Ca2^v^—Ca1—Si1	57.38 (12)	Ca2^xiv^—Ca2—Yb2^viii^	113.77 (8)
Ca2^v^—Ca1—Si1^ix^	48.0 (2)	Ca2^xiv^—Ca2—Yb2^xiv^	0
Ca2^v^—Ca1—Si1^x^	123.45 (19)	Ca2^xiv^—Ca2—Si1	59.17 (5)
Ca2^v^—Ca1—O1	105.2 (3)	Ca2^xiv^—Ca2—Si1^xi^	116.74 (9)
Ca2^v^—Ca1—O1^ix^	33.5 (2)	Ca2^xiv^—Ca2—O1^xi^	116.90 (10)
Ca2^v^—Ca1—O1^x^	70.8 (5)	Ca2^xiv^—Ca2—O3^xi^	144.04 (19)
Ca2^v^—Ca1—O2^vi^	134.6 (4)	Ca2^xiv^—Ca2—O3^xiii^	32.5 (3)
Ca2^v^—Ca1—O2^vii^	71.2 (2)	Ca2^xiv^—Ca2—O3^xv^	134.4 (2)
Ca2^v^—Ca1—O2^viii^	121.5 (3)	Ca2^xiv^—Ca2—O3^xvi^	87.13 (18)
Ca2^vi^—Ca1—Ca2^vii^	103.90 (9)	Ca2^xiv^—Ca2—O4	74.15 (7)
Ca2^vi^—Ca1—Ca2^viii^	103.90 (9)	Yb2—Ca2—Yb2^iv^	0
Ca2^vi^—Ca1—Yb2^iii^	95.97 (4)	Yb2—Ca2—Yb2^xi^	0
Ca2^vi^—Ca1—Yb2^iv^	157.21 (7)	Yb2—Ca2—Yb2^xii^	0
Ca2^vi^—Ca1—Yb2^v^	59.81 (3)	Yb2—Ca2—Yb2^xiii^	0
Ca2^vi^—Ca1—Yb2^vi^	0	Yb2—Ca2—Yb2^viii^	0
Ca2^vi^—Ca1—Yb2^vii^	103.90 (9)	Yb2—Ca2—Yb2^xiv^	0
Ca2^vi^—Ca1—Yb2^viii^	103.90 (9)	Yb2—Ca2—Si1	0
Ca2^vi^—Ca1—Si1	111.00 (13)	Yb2—Ca2—Si1^xi^	0
Ca2^vi^—Ca1—Si1^ix^	59.15 (8)	Yb2—Ca2—O1^xi^	0
Ca2^vi^—Ca1—Si1^x^	143.83 (15)	Yb2—Ca2—O3^xi^	0
Ca2^vi^—Ca1—O1	158.0 (2)	Yb2—Ca2—O3^xiii^	0
Ca2^vi^—Ca1—O1^ix^	93.2 (2)	Yb2—Ca2—O3^xv^	0
Ca2^vi^—Ca1—O1^x^	85.0 (3)	Yb2—Ca2—O3^xvi^	0
Ca2^vi^—Ca1—O2^vi^	108.1 (2)	Yb2—Ca2—O4	0
Ca2^vi^—Ca1—O2^vii^	41.3 (3)	Yb2^iv^—Ca2—Yb2^xi^	60.00 (6)
Ca2^vi^—Ca1—O2^viii^	62.9 (4)	Yb2^iv^—Ca2—Yb2^xii^	90.00 (7)
Ca2^vii^—Ca1—Ca2^viii^	103.90 (10)	Yb2^iv^—Ca2—Yb2^xiii^	90.00 (7)
Ca2^vii^—Ca1—Yb2^iii^	59.81 (4)	Yb2^iv^—Ca2—Yb2^viii^	61.76 (6)
Ca2^vii^—Ca1—Yb2^iv^	95.97 (5)	Yb2^iv^—Ca2—Yb2^xiv^	61.76 (6)
Ca2^vii^—Ca1—Yb2^v^	157.21 (4)	Yb2^iv^—Ca2—Si1	50.27 (16)
Ca2^vii^—Ca1—Yb2^vi^	103.90 (9)	Yb2^iv^—Ca2—Si1^xi^	115.4 (2)
Ca2^vii^—Ca1—Yb2^vii^	0	Yb2^iv^—Ca2—O1^xi^	175.9 (3)
Ca2^vii^—Ca1—Yb2^viii^	103.90 (10)	Yb2^iv^—Ca2—O3^xi^	110.5 (3)
Ca2^vii^—Ca1—Si1	143.83 (13)	Yb2^iv^—Ca2—O3^xiii^	94.0 (3)
Ca2^vii^—Ca1—Si1^ix^	111.0 (2)	Yb2^iv^—Ca2—O3^xv^	94.0 (3)
Ca2^vii^—Ca1—Si1^x^	59.15 (14)	Yb2^iv^—Ca2—O3^xvi^	110.5 (3)
Ca2^vii^—Ca1—O1	85.0 (3)	Yb2^iv^—Ca2—O4	30.00 (5)
Ca2^vii^—Ca1—O1^ix^	158.0 (3)	Yb2^xi^—Ca2—Yb2^xii^	61.76 (4)
Ca2^vii^—Ca1—O1^x^	93.2 (4)	Yb2^xi^—Ca2—Yb2^xiii^	61.76 (4)
Ca2^vii^—Ca1—O2^vi^	62.9 (4)	Yb2^xi^—Ca2—Yb2^viii^	90.00 (6)
Ca2^vii^—Ca1—O2^vii^	108.1 (3)	Yb2^xi^—Ca2—Yb2^xiv^	90.00 (6)
Ca2^vii^—Ca1—O2^viii^	41.3 (4)	Yb2^xi^—Ca2—Si1	110.27 (17)
Ca2^viii^—Ca1—Yb2^iii^	157.21 (5)	Yb2^xi^—Ca2—Si1^xi^	55.4 (2)
Ca2^viii^—Ca1—Yb2^iv^	59.81 (4)	Yb2^xi^—Ca2—O1^xi^	124.1 (3)
Ca2^viii^—Ca1—Yb2^v^	95.97 (5)	Yb2^xi^—Ca2—O3^xi^	59.7 (2)
Ca2^viii^—Ca1—Yb2^vi^	103.90 (9)	Yb2^xi^—Ca2—O3^xiii^	111.47 (15)
Ca2^viii^—Ca1—Yb2^vii^	103.90 (10)	Yb2^xi^—Ca2—O3^xv^	111.47 (15)
Ca2^viii^—Ca1—Yb2^viii^	0	Yb2^xi^—Ca2—O3^xvi^	59.7 (2)
Ca2^viii^—Ca1—Si1	59.15 (8)	Yb2^xi^—Ca2—O4	30.00 (4)
Ca2^viii^—Ca1—Si1^ix^	143.8 (2)	Yb2^xii^—Ca2—Yb2^xiii^	113.77 (5)
Ca2^viii^—Ca1—Si1^x^	111.00 (13)	Yb2^xii^—Ca2—Yb2^viii^	56.48 (4)
Ca2^viii^—Ca1—O1	93.2 (3)	Yb2^xii^—Ca2—Yb2^xiv^	148.29 (9)
Ca2^viii^—Ca1—O1^ix^	85.0 (4)	Yb2^xii^—Ca2—Si1	114.84 (9)
Ca2^viii^—Ca1—O1^x^	158.0 (3)	Yb2^xii^—Ca2—Si1^xi^	60.44 (8)
Ca2^viii^—Ca1—O2^vi^	41.3 (4)	Yb2^xii^—Ca2—O1^xi^	92.24 (16)
Ca2^viii^—Ca1—O2^vii^	62.9 (4)	Yb2^xii^—Ca2—O3^xi^	30.91 (19)
Ca2^viii^—Ca1—O2^viii^	108.1 (3)	Yb2^xii^—Ca2—O3^xiii^	168.7 (2)
Yb2^iii^—Ca1—Yb2^iv^	103.69 (10)	Yb2^xii^—Ca2—O3^xv^	55.76 (15)
Yb2^iii^—Ca1—Yb2^v^	103.69 (9)	Yb2^xii^—Ca2—O3^xvi^	90.09 (17)
Yb2^iii^—Ca1—Yb2^vi^	95.97 (4)	Yb2^xii^—Ca2—O4	74.15 (5)
Yb2^iii^—Ca1—Yb2^vii^	59.81 (4)	Yb2^xiii^—Ca2—Yb2^viii^	148.29 (9)
Yb2^iii^—Ca1—Yb2^viii^	157.21 (5)	Yb2^xiii^—Ca2—Yb2^xiv^	56.48 (4)
Yb2^iii^—Ca1—Si1	123.45 (18)	Yb2^xiii^—Ca2—Si1	114.84 (9)
Yb2^iii^—Ca1—Si1^ix^	57.4 (2)	Yb2^xiii^—Ca2—Si1^xi^	60.44 (8)
Yb2^iii^—Ca1—Si1^x^	47.98 (14)	Yb2^xiii^—Ca2—O1^xi^	92.24 (16)
Yb2^iii^—Ca1—O1	70.8 (3)	Yb2^xiii^—Ca2—O3^xi^	90.09 (17)
Yb2^iii^—Ca1—O1^ix^	105.2 (4)	Yb2^xiii^—Ca2—O3^xiii^	55.76 (15)
Yb2^iii^—Ca1—O1^x^	33.5 (4)	Yb2^xiii^—Ca2—O3^xv^	168.7 (2)
Yb2^iii^—Ca1—O2^vi^	121.5 (4)	Yb2^xiii^—Ca2—O3^xvi^	30.91 (19)
Yb2^iii^—Ca1—O2^vii^	134.6 (4)	Yb2^xiii^—Ca2—O4	74.15 (5)
Yb2^iii^—Ca1—O2^viii^	71.2 (3)	Yb2^viii^—Ca2—Yb2^xiv^	113.77 (8)
Yb2^iv^—Ca1—Yb2^v^	103.69 (9)	Yb2^viii^—Ca2—Si1	59.17 (5)
Yb2^iv^—Ca1—Yb2^vi^	157.21 (7)	Yb2^viii^—Ca2—Si1^xi^	116.74 (9)
Yb2^iv^—Ca1—Yb2^vii^	95.97 (5)	Yb2^viii^—Ca2—O1^xi^	116.90 (10)
Yb2^iv^—Ca1—Yb2^viii^	59.81 (4)	Yb2^viii^—Ca2—O3^xi^	87.13 (18)
Yb2^iv^—Ca1—Si1	47.98 (14)	Yb2^viii^—Ca2—O3^xiii^	134.4 (2)
Yb2^iv^—Ca1—Si1^ix^	123.45 (17)	Yb2^viii^—Ca2—O3^xv^	32.5 (3)
Yb2^iv^—Ca1—Si1^x^	57.38 (16)	Yb2^viii^—Ca2—O3^xvi^	144.04 (19)
Yb2^iv^—Ca1—O1	33.5 (3)	Yb2^viii^—Ca2—O4	74.15 (7)
Yb2^iv^—Ca1—O1^ix^	70.8 (3)	Yb2^xiv^—Ca2—Si1	59.17 (5)
Yb2^iv^—Ca1—O1^x^	105.2 (3)	Yb2^xiv^—Ca2—Si1^xi^	116.74 (9)
Yb2^iv^—Ca1—O2^vi^	71.17 (17)	Yb2^xiv^—Ca2—O1^xi^	116.90 (10)
Yb2^iv^—Ca1—O2^vii^	121.5 (4)	Yb2^xiv^—Ca2—O3^xi^	144.04 (19)
Yb2^iv^—Ca1—O2^viii^	134.6 (3)	Yb2^xiv^—Ca2—O3^xiii^	32.5 (3)
Yb2^v^—Ca1—Yb2^vi^	59.81 (3)	Yb2^xiv^—Ca2—O3^xv^	134.4 (2)
Yb2^v^—Ca1—Yb2^vii^	157.21 (4)	Yb2^xiv^—Ca2—O3^xvi^	87.13 (18)
Yb2^v^—Ca1—Yb2^viii^	95.97 (5)	Yb2^xiv^—Ca2—O4	74.15 (7)
Yb2^v^—Ca1—Si1	57.38 (12)	Si1—Ca2—Si1^xi^	165.7 (3)
Yb2^v^—Ca1—Si1^ix^	48.0 (2)	Si1—Ca2—O1^xi^	125.6 (3)
Yb2^v^—Ca1—Si1^x^	123.45 (19)	Si1—Ca2—O3^xi^	145.7 (2)
Yb2^v^—Ca1—O1	105.2 (3)	Si1—Ca2—O3^xiii^	75.5 (2)
Yb2^v^—Ca1—O1^ix^	33.5 (2)	Si1—Ca2—O3^xv^	75.5 (2)
Yb2^v^—Ca1—O1^x^	70.8 (5)	Si1—Ca2—O3^xvi^	145.7 (2)
Yb2^v^—Ca1—O2^vi^	134.6 (4)	Si1—Ca2—O4	80.27 (18)
Yb2^v^—Ca1—O2^vii^	71.2 (2)	Si1^xi^—Ca2—O1^xi^	68.7 (4)
Yb2^v^—Ca1—O2^viii^	121.5 (3)	Si1^xi^—Ca2—O3^xi^	30.90 (16)
Yb2^vi^—Ca1—Yb2^vii^	103.90 (9)	Si1^xi^—Ca2—O3^xiii^	108.4 (2)
Yb2^vi^—Ca1—Yb2^viii^	103.90 (9)	Si1^xi^—Ca2—O3^xv^	108.4 (2)
Yb2^vi^—Ca1—Si1	111.00 (13)	Si1^xi^—Ca2—O3^xvi^	30.90 (16)
Yb2^vi^—Ca1—Si1^ix^	59.15 (8)	Si1^xi^—Ca2—O4	85.4 (2)
Yb2^vi^—Ca1—Si1^x^	143.83 (15)	O1^xi^—Ca2—O3^xi^	73.0 (3)
Yb2^vi^—Ca1—O1	158.0 (2)	O1^xi^—Ca2—O3^xiii^	84.4 (3)
Yb2^vi^—Ca1—O1^ix^	93.2 (2)	O1^xi^—Ca2—O3^xv^	84.4 (3)
Yb2^vi^—Ca1—O1^x^	85.0 (3)	O1^xi^—Ca2—O3^xvi^	73.0 (3)
Yb2^vi^—Ca1—O2^vi^	108.1 (2)	O1^xi^—Ca2—O4	154.1 (3)
Yb2^vi^—Ca1—O2^vii^	41.3 (3)	O3^xi^—Ca2—O3^xiii^	138.4 (3)
Yb2^vi^—Ca1—O2^viii^	62.9 (4)	O3^xi^—Ca2—O3^xv^	78.6 (2)
Yb2^vii^—Ca1—Yb2^viii^	103.90 (10)	O3^xi^—Ca2—O3^xvi^	61.7 (2)
Yb2^vii^—Ca1—Si1	143.83 (13)	O3^xi^—Ca2—O4	84.9 (3)
Yb2^vii^—Ca1—Si1^ix^	111.0 (2)	O3^xiii^—Ca2—O3^xv^	134.2 (2)
Yb2^vii^—Ca1—Si1^x^	59.15 (14)	O3^xiii^—Ca2—O3^xvi^	78.6 (2)
Yb2^vii^—Ca1—O1	85.0 (3)	O3^xiii^—Ca2—O4	104.6 (2)
Yb2^vii^—Ca1—O1^ix^	158.0 (3)	O3^xv^—Ca2—O3^xvi^	138.4 (3)
Yb2^vii^—Ca1—O1^x^	93.2 (4)	O3^xv^—Ca2—O4	104.6 (2)
Yb2^vii^—Ca1—O2^vi^	62.9 (4)	O3^xvi^—Ca2—O4	84.9 (3)
Yb2^vii^—Ca1—O2^vii^	108.1 (3)	Ca1^xviii^—Yb2—Ca1^xix^	104.16 (6)
Yb2^vii^—Ca1—O2^viii^	41.3 (4)	Ca1^xviii^—Yb2—Ca1^xx^	84.03 (5)
Yb2^viii^—Ca1—Si1	59.15 (8)	Ca1^xviii^—Yb2—Ca1^xxi^	49.54 (10)
Yb2^viii^—Ca1—Si1^ix^	143.8 (2)	Ca1^xviii^—Yb2—Yb1^xviii^	0
Yb2^viii^—Ca1—Si1^x^	111.00 (13)	Ca1^xviii^—Yb2—Yb1^xix^	104.16 (6)
Yb2^viii^—Ca1—O1	93.2 (3)	Ca1^xviii^—Yb2—Yb1^xx^	84.03 (5)
Yb2^viii^—Ca1—O1^ix^	85.0 (4)	Ca1^xviii^—Yb2—Yb1^xxi^	49.54 (10)
Yb2^viii^—Ca1—O1^x^	158.0 (3)	Ca1^xviii^—Yb2—Ca2	0
Yb2^viii^—Ca1—O2^vi^	41.3 (4)	Ca1^xviii^—Yb2—Ca2^iv^	150.19 (6)
Yb2^viii^—Ca1—O2^vii^	62.9 (4)	Ca1^xviii^—Yb2—Ca2^xi^	101.65 (6)
Yb2^viii^—Ca1—O2^viii^	108.1 (3)	Ca1^xviii^—Yb2—Ca2^xii^	60.19 (8)
Si1—Ca1—Si1^ix^	95.2 (2)	Ca1^xviii^—Yb2—Ca2^xiii^	101.81 (10)
Si1—Ca1—Si1^x^	95.2 (2)	Ca1^xviii^—Yb2—Ca2^viii^	97.63 (8)
Si1—Ca1—O1	66.1 (4)	Ca1^xviii^—Yb2—Ca2^xiv^	146.57 (9)
Si1—Ca1—O1^ix^	29.2 (4)	Ca1^xviii^—Yb2—Yb2^iv^	150.19 (6)
Si1—Ca1—O1^x^	98.9 (4)	Ca1^xviii^—Yb2—Yb2^xi^	101.65 (6)
Si1—Ca1—O2^vi^	96.5 (3)	Ca1^xviii^—Yb2—Yb2^xii^	60.19 (8)
Si1—Ca1—O2^vii^	92.6 (4)	Ca1^xviii^—Yb2—Yb2^xiii^	101.81 (10)
Si1—Ca1—O2^viii^	165.3 (4)	Ca1^xviii^—Yb2—Yb2^viii^	97.63 (8)
Si1^ix^—Ca1—Si1^x^	95.2 (2)	Ca1^xviii^—Yb2—Yb2^xiv^	146.57 (9)
Si1^ix^—Ca1—O1	98.9 (2)	Ca1^xviii^—Yb2—Si1	139.51 (13)
Si1^ix^—Ca1—O1^ix^	66.1 (4)	Ca1^xviii^—Yb2—Si1^xi^	52.1 (2)
Si1^ix^—Ca1—O1^x^	29.2 (4)	Ca1^xviii^—Yb2—O3^xiii^	115.3 (3)
Si1^ix^—Ca1—O2^vi^	165.3 (3)	Ca1^xviii^—Yb2—O3^xv^	69.9 (3)
Si1^ix^—Ca1—O2^vii^	96.5 (3)	Ca1^xviii^—Yb2—O4	128.14 (6)
Si1^ix^—Ca1—O2^viii^	92.6 (3)	Ca1^xix^—Yb2—Ca1^xx^	49.19 (10)
Si1^x^—Ca1—O1	29.2 (4)	Ca1^xix^—Yb2—Ca1^xxi^	84.03 (5)
Si1^x^—Ca1—O1^ix^	98.9 (3)	Ca1^xix^—Yb2—Yb1^xviii^	104.16 (6)
Si1^x^—Ca1—O1^x^	66.1 (4)	Ca1^xix^—Yb2—Yb1^xix^	0
Si1^x^—Ca1—O2^vi^	92.6 (3)	Ca1^xix^—Yb2—Yb1^xx^	49.19 (10)
Si1^x^—Ca1—O2^vii^	165.3 (3)	Ca1^xix^—Yb2—Yb1^xxi^	84.03 (5)
Si1^x^—Ca1—O2^viii^	96.5 (4)	Ca1^xix^—Yb2—Ca2	0
O1—Ca1—O1^ix^	74.3 (4)	Ca1^xix^—Yb2—Ca2^iv^	101.13 (6)
O1—Ca1—O1^x^	74.3 (4)	Ca1^xix^—Yb2—Ca2^xi^	150.00 (8)
O1—Ca1—O2^vi^	93.9 (2)	Ca1^xix^—Yb2—Ca2^xii^	146.51 (8)
O1—Ca1—O2^vii^	154.6 (5)	Ca1^xix^—Yb2—Ca2^xiii^	97.87 (7)
O1—Ca1—O2^viii^	124.9 (5)	Ca1^xix^—Yb2—Ca2^viii^	101.37 (8)
O1^ix^—Ca1—O1^x^	74.3 (6)	Ca1^xix^—Yb2—Ca2^xiv^	60.00 (7)
O1^ix^—Ca1—O2^vi^	124.9 (5)	Ca1^xix^—Yb2—Yb2^iv^	101.13 (6)
O1^ix^—Ca1—O2^vii^	93.9 (4)	Ca1^xix^—Yb2—Yb2^xi^	150.00 (8)
O1^ix^—Ca1—O2^viii^	154.6 (4)	Ca1^xix^—Yb2—Yb2^xii^	146.51 (8)
O1^x^—Ca1—O2^vi^	154.6 (6)	Ca1^xix^—Yb2—Yb2^xiii^	97.87 (7)
O1^x^—Ca1—O2^vii^	124.9 (4)	Ca1^xix^—Yb2—Yb2^viii^	101.37 (8)
O1^x^—Ca1—O2^viii^	93.9 (4)	Ca1^xix^—Yb2—Yb2^xiv^	60.00 (7)
O2^vi^—Ca1—O2^vii^	74.1 (4)	Ca1^xix^—Yb2—Si1	55.95 (14)
O2^vi^—Ca1—O2^viii^	74.1 (4)	Ca1^xix^—Yb2—Si1^xi^	136.0 (2)
O2^vii^—Ca1—O2^viii^	74.1 (6)	Ca1^xix^—Yb2—O3^xiii^	42.54 (16)
Ca1—Yb1—Ca1^i^	0	Ca1^xix^—Yb2—O3^xv^	91.72 (16)
Ca1—Yb1—Ca1^ii^	0	Ca1^xix^—Yb2—O4	127.70 (9)
Ca1—Yb1—Yb1^i^	0	Ca1^xx^—Yb2—Ca1^xxi^	104.16 (6)
Ca1—Yb1—Yb1^ii^	0	Ca1^xx^—Yb2—Yb1^xviii^	84.03 (5)
Ca1—Yb1—Ca2^iii^	0	Ca1^xx^—Yb2—Yb1^xix^	49.19 (10)
Ca1—Yb1—Ca2^iv^	0	Ca1^xx^—Yb2—Yb1^xx^	0
Ca1—Yb1—Ca2^v^	0	Ca1^xx^—Yb2—Yb1^xxi^	104.16 (6)
Ca1—Yb1—Ca2^vi^	0	Ca1^xx^—Yb2—Ca2	0
Ca1—Yb1—Ca2^vii^	0	Ca1^xx^—Yb2—Ca2^iv^	101.13 (6)
Ca1—Yb1—Ca2^viii^	0	Ca1^xx^—Yb2—Ca2^xi^	150.00 (8)
Ca1—Yb1—Yb2^iii^	0	Ca1^xx^—Yb2—Ca2^xii^	97.87 (7)
Ca1—Yb1—Yb2^iv^	0	Ca1^xx^—Yb2—Ca2^xiii^	146.51 (8)
Ca1—Yb1—Yb2^v^	0	Ca1^xx^—Yb2—Ca2^viii^	60.00 (7)
Ca1—Yb1—Yb2^vi^	0	Ca1^xx^—Yb2—Ca2^xiv^	101.37 (8)
Ca1—Yb1—Yb2^vii^	0	Ca1^xx^—Yb2—Yb2^iv^	101.13 (6)
Ca1—Yb1—Yb2^viii^	0	Ca1^xx^—Yb2—Yb2^xi^	150.00 (8)
Ca1—Yb1—Si1	0	Ca1^xx^—Yb2—Yb2^xii^	97.87 (7)
Ca1—Yb1—Si1^ix^	0	Ca1^xx^—Yb2—Yb2^xiii^	146.51 (8)
Ca1—Yb1—Si1^x^	0	Ca1^xx^—Yb2—Yb2^viii^	60.00 (7)
Ca1—Yb1—O2^vi^	0	Ca1^xx^—Yb2—Yb2^xiv^	101.37 (8)
Ca1—Yb1—O2^vii^	0	Ca1^xx^—Yb2—Si1	55.95 (14)
Ca1—Yb1—O2^viii^	0	Ca1^xx^—Yb2—Si1^xi^	136.0 (2)
Ca1^i^—Yb1—Ca1^ii^	180	Ca1^xx^—Yb2—O3^xiii^	91.72 (16)
Ca1^i^—Yb1—Yb1^i^	0	Ca1^xx^—Yb2—O3^xv^	42.54 (16)
Ca1^i^—Yb1—Yb1^ii^	180	Ca1^xx^—Yb2—O4	127.70 (9)
Ca1^i^—Yb1—Ca2^iii^	114.77 (7)	Ca1^xxi^—Yb2—Yb1^xviii^	49.54 (10)
Ca1^i^—Yb1—Ca2^iv^	114.77 (7)	Ca1^xxi^—Yb2—Yb1^xix^	84.03 (5)
Ca1^i^—Yb1—Ca2^v^	114.77 (7)	Ca1^xxi^—Yb2—Yb1^xx^	104.16 (6)
Ca1^i^—Yb1—Ca2^vi^	65.41 (7)	Ca1^xxi^—Yb2—Yb1^xxi^	0
Ca1^i^—Yb1—Ca2^vii^	65.41 (7)	Ca1^xxi^—Yb2—Ca2	0
Ca1^i^—Yb1—Ca2^viii^	65.41 (7)	Ca1^xxi^—Yb2—Ca2^iv^	150.19 (6)
Ca1^i^—Yb1—Yb2^iii^	114.77 (7)	Ca1^xxi^—Yb2—Ca2^xi^	101.65 (6)
Ca1^i^—Yb1—Yb2^iv^	114.77 (7)	Ca1^xxi^—Yb2—Ca2^xii^	101.81 (10)
Ca1^i^—Yb1—Yb2^v^	114.77 (7)	Ca1^xxi^—Yb2—Ca2^xiii^	60.19 (8)
Ca1^i^—Yb1—Yb2^vi^	65.41 (7)	Ca1^xxi^—Yb2—Ca2^viii^	146.57 (9)
Ca1^i^—Yb1—Yb2^vii^	65.41 (7)	Ca1^xxi^—Yb2—Ca2^xiv^	97.63 (8)
Ca1^i^—Yb1—Yb2^viii^	65.41 (7)	Ca1^xxi^—Yb2—Yb2^iv^	150.19 (6)
Ca1^i^—Yb1—Si1	121.53 (12)	Ca1^xxi^—Yb2—Yb2^xi^	101.65 (6)
Ca1^i^—Yb1—Si1^ix^	121.53 (11)	Ca1^xxi^—Yb2—Yb2^xii^	101.81 (10)
Ca1^i^—Yb1—Si1^x^	121.53 (14)	Ca1^xxi^—Yb2—Yb2^xiii^	60.19 (8)
Ca1^i^—Yb1—O2^vi^	44.11 (18)	Ca1^xxi^—Yb2—Yb2^viii^	146.57 (9)
Ca1^i^—Yb1—O2^vii^	44.1 (3)	Ca1^xxi^—Yb2—Yb2^xiv^	97.63 (8)
Ca1^i^—Yb1—O2^viii^	44.1 (3)	Ca1^xxi^—Yb2—Si1	139.51 (13)
Ca1^ii^—Yb1—Yb1^i^	180	Ca1^xxi^—Yb2—Si1^xi^	52.1 (2)
Ca1^ii^—Yb1—Yb1^ii^	0	Ca1^xxi^—Yb2—O3^xiii^	69.9 (3)
Ca1^ii^—Yb1—Ca2^iii^	65.23 (7)	Ca1^xxi^—Yb2—O3^xv^	115.3 (3)
Ca1^ii^—Yb1—Ca2^iv^	65.23 (7)	Ca1^xxi^—Yb2—O4	128.14 (6)
Ca1^ii^—Yb1—Ca2^v^	65.23 (7)	Yb1^xviii^—Yb2—Yb1^xix^	104.16 (6)
Ca1^ii^—Yb1—Ca2^vi^	114.59 (7)	Yb1^xviii^—Yb2—Yb1^xx^	84.03 (5)
Ca1^ii^—Yb1—Ca2^vii^	114.59 (7)	Yb1^xviii^—Yb2—Yb1^xxi^	49.54 (10)
Ca1^ii^—Yb1—Ca2^viii^	114.59 (7)	Yb1^xviii^—Yb2—Ca2	0
Ca1^ii^—Yb1—Yb2^iii^	65.23 (7)	Yb1^xviii^—Yb2—Ca2^iv^	150.19 (6)
Ca1^ii^—Yb1—Yb2^iv^	65.23 (7)	Yb1^xviii^—Yb2—Ca2^xi^	101.65 (6)
Ca1^ii^—Yb1—Yb2^v^	65.23 (7)	Yb1^xviii^—Yb2—Ca2^xii^	60.19 (8)
Ca1^ii^—Yb1—Yb2^vi^	114.59 (7)	Yb1^xviii^—Yb2—Ca2^xiii^	101.81 (10)
Ca1^ii^—Yb1—Yb2^vii^	114.59 (7)	Yb1^xviii^—Yb2—Ca2^viii^	97.63 (8)
Ca1^ii^—Yb1—Yb2^viii^	114.59 (7)	Yb1^xviii^—Yb2—Ca2^xiv^	146.57 (9)
Ca1^ii^—Yb1—Si1	58.47 (12)	Yb1^xviii^—Yb2—Yb2^iv^	150.19 (6)
Ca1^ii^—Yb1—Si1^ix^	58.47 (11)	Yb1^xviii^—Yb2—Yb2^xi^	101.65 (6)
Ca1^ii^—Yb1—Si1^x^	58.47 (14)	Yb1^xviii^—Yb2—Yb2^xii^	60.19 (8)
Ca1^ii^—Yb1—O2^vi^	135.89 (18)	Yb1^xviii^—Yb2—Yb2^xiii^	101.81 (10)
Ca1^ii^—Yb1—O2^vii^	135.9 (3)	Yb1^xviii^—Yb2—Yb2^viii^	97.63 (8)
Ca1^ii^—Yb1—O2^viii^	135.9 (3)	Yb1^xviii^—Yb2—Yb2^xiv^	146.57 (9)
Yb1^i^—Yb1—Yb1^ii^	180	Yb1^xviii^—Yb2—Si1	139.51 (13)
Yb1^i^—Yb1—Ca2^iii^	114.77 (7)	Yb1^xviii^—Yb2—Si1^xi^	52.1 (2)
Yb1^i^—Yb1—Ca2^iv^	114.77 (7)	Yb1^xviii^—Yb2—O3^xiii^	115.3 (3)
Yb1^i^—Yb1—Ca2^v^	114.77 (7)	Yb1^xviii^—Yb2—O3^xv^	69.9 (3)
Yb1^i^—Yb1—Ca2^vi^	65.41 (7)	Yb1^xviii^—Yb2—O4	128.14 (6)
Yb1^i^—Yb1—Ca2^vii^	65.41 (7)	Yb1^xix^—Yb2—Yb1^xx^	49.19 (10)
Yb1^i^—Yb1—Ca2^viii^	65.41 (7)	Yb1^xix^—Yb2—Yb1^xxi^	84.03 (5)
Yb1^i^—Yb1—Yb2^iii^	114.77 (7)	Yb1^xix^—Yb2—Ca2	0
Yb1^i^—Yb1—Yb2^iv^	114.77 (7)	Yb1^xix^—Yb2—Ca2^iv^	101.13 (6)
Yb1^i^—Yb1—Yb2^v^	114.77 (7)	Yb1^xix^—Yb2—Ca2^xi^	150.00 (8)
Yb1^i^—Yb1—Yb2^vi^	65.41 (7)	Yb1^xix^—Yb2—Ca2^xii^	146.51 (8)
Yb1^i^—Yb1—Yb2^vii^	65.41 (7)	Yb1^xix^—Yb2—Ca2^xiii^	97.87 (7)
Yb1^i^—Yb1—Yb2^viii^	65.41 (7)	Yb1^xix^—Yb2—Ca2^viii^	101.37 (8)
Yb1^i^—Yb1—Si1	121.53 (12)	Yb1^xix^—Yb2—Ca2^xiv^	60.00 (7)
Yb1^i^—Yb1—Si1^ix^	121.53 (11)	Yb1^xix^—Yb2—Yb2^iv^	101.13 (6)
Yb1^i^—Yb1—Si1^x^	121.53 (14)	Yb1^xix^—Yb2—Yb2^xi^	150.00 (8)
Yb1^i^—Yb1—O2^vi^	44.11 (18)	Yb1^xix^—Yb2—Yb2^xii^	146.51 (8)
Yb1^i^—Yb1—O2^vii^	44.1 (3)	Yb1^xix^—Yb2—Yb2^xiii^	97.87 (7)
Yb1^i^—Yb1—O2^viii^	44.1 (3)	Yb1^xix^—Yb2—Yb2^viii^	101.37 (8)
Yb1^ii^—Yb1—Ca2^iii^	65.23 (7)	Yb1^xix^—Yb2—Yb2^xiv^	60.00 (7)
Yb1^ii^—Yb1—Ca2^iv^	65.23 (7)	Yb1^xix^—Yb2—Si1	55.95 (14)
Yb1^ii^—Yb1—Ca2^v^	65.23 (7)	Yb1^xix^—Yb2—Si1^xi^	136.0 (2)
Yb1^ii^—Yb1—Ca2^vi^	114.59 (7)	Yb1^xix^—Yb2—O3^xiii^	42.54 (16)
Yb1^ii^—Yb1—Ca2^vii^	114.59 (7)	Yb1^xix^—Yb2—O3^xv^	91.72 (16)
Yb1^ii^—Yb1—Ca2^viii^	114.59 (7)	Yb1^xix^—Yb2—O4	127.70 (9)
Yb1^ii^—Yb1—Yb2^iii^	65.23 (7)	Yb1^xx^—Yb2—Yb1^xxi^	104.16 (6)
Yb1^ii^—Yb1—Yb2^iv^	65.23 (7)	Yb1^xx^—Yb2—Ca2	0
Yb1^ii^—Yb1—Yb2^v^	65.23 (7)	Yb1^xx^—Yb2—Ca2^iv^	101.13 (6)
Yb1^ii^—Yb1—Yb2^vi^	114.59 (7)	Yb1^xx^—Yb2—Ca2^xi^	150.00 (8)
Yb1^ii^—Yb1—Yb2^vii^	114.59 (7)	Yb1^xx^—Yb2—Ca2^xii^	97.87 (7)
Yb1^ii^—Yb1—Yb2^viii^	114.59 (7)	Yb1^xx^—Yb2—Ca2^xiii^	146.51 (8)
Yb1^ii^—Yb1—Si1	58.47 (12)	Yb1^xx^—Yb2—Ca2^viii^	60.00 (7)
Yb1^ii^—Yb1—Si1^ix^	58.47 (11)	Yb1^xx^—Yb2—Ca2^xiv^	101.37 (8)
Yb1^ii^—Yb1—Si1^x^	58.47 (14)	Yb1^xx^—Yb2—Yb2^iv^	101.13 (6)
Yb1^ii^—Yb1—O2^vi^	135.89 (18)	Yb1^xx^—Yb2—Yb2^xi^	150.00 (8)
Yb1^ii^—Yb1—O2^vii^	135.9 (3)	Yb1^xx^—Yb2—Yb2^xii^	97.87 (7)
Yb1^ii^—Yb1—O2^viii^	135.9 (3)	Yb1^xx^—Yb2—Yb2^xiii^	146.51 (8)
Ca2^iii^—Yb1—Ca2^iv^	103.69 (10)	Yb1^xx^—Yb2—Yb2^viii^	60.00 (7)
Ca2^iii^—Yb1—Ca2^v^	103.69 (9)	Yb1^xx^—Yb2—Yb2^xiv^	101.37 (8)
Ca2^iii^—Yb1—Ca2^vi^	95.97 (4)	Yb1^xx^—Yb2—Si1	55.95 (14)
Ca2^iii^—Yb1—Ca2^vii^	59.81 (4)	Yb1^xx^—Yb2—Si1^xi^	136.0 (2)
Ca2^iii^—Yb1—Ca2^viii^	157.21 (5)	Yb1^xx^—Yb2—O3^xiii^	91.72 (16)
Ca2^iii^—Yb1—Yb2^iii^	0	Yb1^xx^—Yb2—O3^xv^	42.54 (16)
Ca2^iii^—Yb1—Yb2^iv^	103.69 (10)	Yb1^xx^—Yb2—O4	127.70 (9)
Ca2^iii^—Yb1—Yb2^v^	103.69 (9)	Yb1^xxi^—Yb2—Ca2	0
Ca2^iii^—Yb1—Yb2^vi^	95.97 (4)	Yb1^xxi^—Yb2—Ca2^iv^	150.19 (6)
Ca2^iii^—Yb1—Yb2^vii^	59.81 (4)	Yb1^xxi^—Yb2—Ca2^xi^	101.65 (6)
Ca2^iii^—Yb1—Yb2^viii^	157.21 (5)	Yb1^xxi^—Yb2—Ca2^xii^	101.81 (10)
Ca2^iii^—Yb1—Si1	123.45 (18)	Yb1^xxi^—Yb2—Ca2^xiii^	60.19 (8)
Ca2^iii^—Yb1—Si1^ix^	57.4 (2)	Yb1^xxi^—Yb2—Ca2^viii^	146.57 (9)
Ca2^iii^—Yb1—Si1^x^	47.98 (14)	Yb1^xxi^—Yb2—Ca2^xiv^	97.63 (8)
Ca2^iii^—Yb1—O2^vi^	121.5 (4)	Yb1^xxi^—Yb2—Yb2^iv^	150.19 (6)
Ca2^iii^—Yb1—O2^vii^	134.6 (4)	Yb1^xxi^—Yb2—Yb2^xi^	101.65 (6)
Ca2^iii^—Yb1—O2^viii^	71.2 (3)	Yb1^xxi^—Yb2—Yb2^xii^	101.81 (10)
Ca2^iv^—Yb1—Ca2^v^	103.69 (9)	Yb1^xxi^—Yb2—Yb2^xiii^	60.19 (8)
Ca2^iv^—Yb1—Ca2^vi^	157.21 (7)	Yb1^xxi^—Yb2—Yb2^viii^	146.57 (9)
Ca2^iv^—Yb1—Ca2^vii^	95.97 (5)	Yb1^xxi^—Yb2—Yb2^xiv^	97.63 (8)
Ca2^iv^—Yb1—Ca2^viii^	59.81 (4)	Yb1^xxi^—Yb2—Si1	139.51 (13)
Ca2^iv^—Yb1—Yb2^iii^	103.69 (10)	Yb1^xxi^—Yb2—Si1^xi^	52.1 (2)
Ca2^iv^—Yb1—Yb2^iv^	0	Yb1^xxi^—Yb2—O3^xiii^	69.9 (3)
Ca2^iv^—Yb1—Yb2^v^	103.69 (9)	Yb1^xxi^—Yb2—O3^xv^	115.3 (3)
Ca2^iv^—Yb1—Yb2^vi^	157.21 (7)	Yb1^xxi^—Yb2—O4	128.14 (6)
Ca2^iv^—Yb1—Yb2^vii^	95.97 (5)	Ca2—Yb2—Ca2^iv^	0
Ca2^iv^—Yb1—Yb2^viii^	59.81 (4)	Ca2—Yb2—Ca2^xi^	0
Ca2^iv^—Yb1—Si1	47.98 (14)	Ca2—Yb2—Ca2^xii^	0
Ca2^iv^—Yb1—Si1^ix^	123.45 (17)	Ca2—Yb2—Ca2^xiii^	0
Ca2^iv^—Yb1—Si1^x^	57.38 (16)	Ca2—Yb2—Ca2^viii^	0
Ca2^iv^—Yb1—O2^vi^	71.17 (17)	Ca2—Yb2—Ca2^xiv^	0
Ca2^iv^—Yb1—O2^vii^	121.5 (4)	Ca2—Yb2—Yb2^iv^	0
Ca2^iv^—Yb1—O2^viii^	134.6 (3)	Ca2—Yb2—Yb2^xi^	0
Ca2^v^—Yb1—Ca2^vi^	59.81 (3)	Ca2—Yb2—Yb2^xii^	0
Ca2^v^—Yb1—Ca2^vii^	157.21 (4)	Ca2—Yb2—Yb2^xiii^	0
Ca2^v^—Yb1—Ca2^viii^	95.97 (5)	Ca2—Yb2—Yb2^viii^	0
Ca2^v^—Yb1—Yb2^iii^	103.69 (9)	Ca2—Yb2—Yb2^xiv^	0
Ca2^v^—Yb1—Yb2^iv^	103.69 (9)	Ca2—Yb2—Si1	0
Ca2^v^—Yb1—Yb2^v^	0	Ca2—Yb2—Si1^xi^	0
Ca2^v^—Yb1—Yb2^vi^	59.81 (3)	Ca2—Yb2—O3^xiii^	0
Ca2^v^—Yb1—Yb2^vii^	157.21 (4)	Ca2—Yb2—O3^xv^	0
Ca2^v^—Yb1—Yb2^viii^	95.97 (5)	Ca2—Yb2—O4	0
Ca2^v^—Yb1—Si1	57.38 (12)	Ca2^iv^—Yb2—Ca2^xi^	60.00 (6)
Ca2^v^—Yb1—Si1^ix^	48.0 (2)	Ca2^iv^—Yb2—Ca2^xii^	90.00 (7)
Ca2^v^—Yb1—Si1^x^	123.45 (19)	Ca2^iv^—Yb2—Ca2^xiii^	90.00 (7)
Ca2^v^—Yb1—O2^vi^	134.6 (4)	Ca2^iv^—Yb2—Ca2^viii^	61.76 (6)
Ca2^v^—Yb1—O2^vii^	71.2 (2)	Ca2^iv^—Yb2—Ca2^xiv^	61.76 (6)
Ca2^v^—Yb1—O2^viii^	121.5 (3)	Ca2^iv^—Yb2—Yb2^iv^	0
Ca2^vi^—Yb1—Ca2^vii^	103.90 (9)	Ca2^iv^—Yb2—Yb2^xi^	60.00 (6)
Ca2^vi^—Yb1—Ca2^viii^	103.90 (9)	Ca2^iv^—Yb2—Yb2^xii^	90.00 (7)
Ca2^vi^—Yb1—Yb2^iii^	95.97 (4)	Ca2^iv^—Yb2—Yb2^xiii^	90.00 (7)
Ca2^vi^—Yb1—Yb2^iv^	157.21 (7)	Ca2^iv^—Yb2—Yb2^viii^	61.76 (6)
Ca2^vi^—Yb1—Yb2^v^	59.81 (3)	Ca2^iv^—Yb2—Yb2^xiv^	61.76 (6)
Ca2^vi^—Yb1—Yb2^vi^	0	Ca2^iv^—Yb2—Si1	50.27 (16)
Ca2^vi^—Yb1—Yb2^vii^	103.90 (9)	Ca2^iv^—Yb2—Si1^xi^	115.4 (2)
Ca2^vi^—Yb1—Yb2^viii^	103.90 (9)	Ca2^iv^—Yb2—O3^xiii^	94.0 (3)
Ca2^vi^—Yb1—Si1	111.00 (13)	Ca2^iv^—Yb2—O3^xv^	94.0 (3)
Ca2^vi^—Yb1—Si1^ix^	59.15 (8)	Ca2^iv^—Yb2—O4	30.00 (5)
Ca2^vi^—Yb1—Si1^x^	143.83 (15)	Ca2^xi^—Yb2—Ca2^xii^	61.76 (4)
Ca2^vi^—Yb1—O2^vi^	108.1 (2)	Ca2^xi^—Yb2—Ca2^xiii^	61.76 (4)
Ca2^vi^—Yb1—O2^vii^	41.3 (3)	Ca2^xi^—Yb2—Ca2^viii^	90.00 (6)
Ca2^vi^—Yb1—O2^viii^	62.9 (4)	Ca2^xi^—Yb2—Ca2^xiv^	90.00 (6)
Ca2^vii^—Yb1—Ca2^viii^	103.90 (10)	Ca2^xi^—Yb2—Yb2^iv^	60.00 (6)
Ca2^vii^—Yb1—Yb2^iii^	59.81 (4)	Ca2^xi^—Yb2—Yb2^xi^	0
Ca2^vii^—Yb1—Yb2^iv^	95.97 (5)	Ca2^xi^—Yb2—Yb2^xii^	61.76 (4)
Ca2^vii^—Yb1—Yb2^v^	157.21 (4)	Ca2^xi^—Yb2—Yb2^xiii^	61.76 (4)
Ca2^vii^—Yb1—Yb2^vi^	103.90 (9)	Ca2^xi^—Yb2—Yb2^viii^	90.00 (6)
Ca2^vii^—Yb1—Yb2^vii^	0	Ca2^xi^—Yb2—Yb2^xiv^	90.00 (6)
Ca2^vii^—Yb1—Yb2^viii^	103.90 (10)	Ca2^xi^—Yb2—Si1	110.27 (17)
Ca2^vii^—Yb1—Si1	143.83 (13)	Ca2^xi^—Yb2—Si1^xi^	55.4 (2)
Ca2^vii^—Yb1—Si1^ix^	111.0 (2)	Ca2^xi^—Yb2—O3^xiii^	111.47 (15)
Ca2^vii^—Yb1—Si1^x^	59.15 (14)	Ca2^xi^—Yb2—O3^xv^	111.47 (15)
Ca2^vii^—Yb1—O2^vi^	62.9 (4)	Ca2^xi^—Yb2—O4	30.00 (4)
Ca2^vii^—Yb1—O2^vii^	108.1 (3)	Ca2^xii^—Yb2—Ca2^xiii^	113.77 (5)
Ca2^vii^—Yb1—O2^viii^	41.3 (4)	Ca2^xii^—Yb2—Ca2^viii^	56.48 (4)
Ca2^viii^—Yb1—Yb2^iii^	157.21 (5)	Ca2^xii^—Yb2—Ca2^xiv^	148.29 (9)
Ca2^viii^—Yb1—Yb2^iv^	59.81 (4)	Ca2^xii^—Yb2—Yb2^iv^	90.00 (7)
Ca2^viii^—Yb1—Yb2^v^	95.97 (5)	Ca2^xii^—Yb2—Yb2^xi^	61.76 (4)
Ca2^viii^—Yb1—Yb2^vi^	103.90 (9)	Ca2^xii^—Yb2—Yb2^xii^	0
Ca2^viii^—Yb1—Yb2^vii^	103.90 (10)	Ca2^xii^—Yb2—Yb2^xiii^	113.77 (5)
Ca2^viii^—Yb1—Yb2^viii^	0	Ca2^xii^—Yb2—Yb2^viii^	56.48 (4)
Ca2^viii^—Yb1—Si1	59.15 (8)	Ca2^xii^—Yb2—Yb2^xiv^	148.29 (9)
Ca2^viii^—Yb1—Si1^ix^	143.8 (2)	Ca2^xii^—Yb2—Si1	114.84 (9)
Ca2^viii^—Yb1—Si1^x^	111.00 (13)	Ca2^xii^—Yb2—Si1^xi^	60.44 (8)
Ca2^viii^—Yb1—O2^vi^	41.3 (4)	Ca2^xii^—Yb2—O3^xiii^	168.7 (2)
Ca2^viii^—Yb1—O2^vii^	62.9 (4)	Ca2^xii^—Yb2—O3^xv^	55.76 (15)
Ca2^viii^—Yb1—O2^viii^	108.1 (3)	Ca2^xii^—Yb2—O4	74.15 (5)
Yb2^iii^—Yb1—Yb2^iv^	103.69 (10)	Ca2^xiii^—Yb2—Ca2^viii^	148.29 (9)
Yb2^iii^—Yb1—Yb2^v^	103.69 (9)	Ca2^xiii^—Yb2—Ca2^xiv^	56.48 (4)
Yb2^iii^—Yb1—Yb2^vi^	95.97 (4)	Ca2^xiii^—Yb2—Yb2^iv^	90.00 (7)
Yb2^iii^—Yb1—Yb2^vii^	59.81 (4)	Ca2^xiii^—Yb2—Yb2^xi^	61.76 (4)
Yb2^iii^—Yb1—Yb2^viii^	157.21 (5)	Ca2^xiii^—Yb2—Yb2^xii^	113.77 (5)
Yb2^iii^—Yb1—Si1	123.45 (18)	Ca2^xiii^—Yb2—Yb2^xiii^	0
Yb2^iii^—Yb1—Si1^ix^	57.4 (2)	Ca2^xiii^—Yb2—Yb2^viii^	148.29 (9)
Yb2^iii^—Yb1—Si1^x^	47.98 (14)	Ca2^xiii^—Yb2—Yb2^xiv^	56.48 (4)
Yb2^iii^—Yb1—O2^vi^	121.5 (4)	Ca2^xiii^—Yb2—Si1	114.84 (9)
Yb2^iii^—Yb1—O2^vii^	134.6 (4)	Ca2^xiii^—Yb2—Si1^xi^	60.44 (8)
Yb2^iii^—Yb1—O2^viii^	71.2 (3)	Ca2^xiii^—Yb2—O3^xiii^	55.76 (15)
Yb2^iv^—Yb1—Yb2^v^	103.69 (9)	Ca2^xiii^—Yb2—O3^xv^	168.7 (2)
Yb2^iv^—Yb1—Yb2^vi^	157.21 (7)	Ca2^xiii^—Yb2—O4	74.15 (5)
Yb2^iv^—Yb1—Yb2^vii^	95.97 (5)	Ca2^viii^—Yb2—Ca2^xiv^	113.77 (8)
Yb2^iv^—Yb1—Yb2^viii^	59.81 (4)	Ca2^viii^—Yb2—Yb2^iv^	61.76 (6)
Yb2^iv^—Yb1—Si1	47.98 (14)	Ca2^viii^—Yb2—Yb2^xi^	90.00 (6)
Yb2^iv^—Yb1—Si1^ix^	123.45 (17)	Ca2^viii^—Yb2—Yb2^xii^	56.48 (4)
Yb2^iv^—Yb1—Si1^x^	57.38 (16)	Ca2^viii^—Yb2—Yb2^xiii^	148.29 (9)
Yb2^iv^—Yb1—O2^vi^	71.17 (17)	Ca2^viii^—Yb2—Yb2^viii^	0
Yb2^iv^—Yb1—O2^vii^	121.5 (4)	Ca2^viii^—Yb2—Yb2^xiv^	113.77 (8)
Yb2^iv^—Yb1—O2^viii^	134.6 (3)	Ca2^viii^—Yb2—Si1	59.17 (5)
Yb2^v^—Yb1—Yb2^vi^	59.81 (3)	Ca2^viii^—Yb2—Si1^xi^	116.74 (9)
Yb2^v^—Yb1—Yb2^vii^	157.21 (4)	Ca2^viii^—Yb2—O3^xiii^	134.4 (2)
Yb2^v^—Yb1—Yb2^viii^	95.97 (5)	Ca2^viii^—Yb2—O3^xv^	32.5 (3)
Yb2^v^—Yb1—Si1	57.38 (12)	Ca2^viii^—Yb2—O4	74.15 (7)
Yb2^v^—Yb1—Si1^ix^	48.0 (2)	Ca2^xiv^—Yb2—Yb2^iv^	61.76 (6)
Yb2^v^—Yb1—Si1^x^	123.45 (19)	Ca2^xiv^—Yb2—Yb2^xi^	90.00 (6)
Yb2^v^—Yb1—O2^vi^	134.6 (4)	Ca2^xiv^—Yb2—Yb2^xii^	148.29 (9)
Yb2^v^—Yb1—O2^vii^	71.2 (2)	Ca2^xiv^—Yb2—Yb2^xiii^	56.48 (4)
Yb2^v^—Yb1—O2^viii^	121.5 (3)	Ca2^xiv^—Yb2—Yb2^viii^	113.77 (8)
Yb2^vi^—Yb1—Yb2^vii^	103.90 (9)	Ca2^xiv^—Yb2—Yb2^xiv^	0
Yb2^vi^—Yb1—Yb2^viii^	103.90 (9)	Ca2^xiv^—Yb2—Si1	59.17 (5)
Yb2^vi^—Yb1—Si1	111.00 (13)	Ca2^xiv^—Yb2—Si1^xi^	116.74 (9)
Yb2^vi^—Yb1—Si1^ix^	59.15 (8)	Ca2^xiv^—Yb2—O3^xiii^	32.5 (3)
Yb2^vi^—Yb1—Si1^x^	143.83 (15)	Ca2^xiv^—Yb2—O3^xv^	134.4 (2)
Yb2^vi^—Yb1—O2^vi^	108.1 (2)	Ca2^xiv^—Yb2—O4	74.15 (7)
Yb2^vi^—Yb1—O2^vii^	41.3 (3)	Yb2^iv^—Yb2—Yb2^xi^	60.00 (6)
Yb2^vi^—Yb1—O2^viii^	62.9 (4)	Yb2^iv^—Yb2—Yb2^xii^	90.00 (7)
Yb2^vii^—Yb1—Yb2^viii^	103.90 (10)	Yb2^iv^—Yb2—Yb2^xiii^	90.00 (7)
Yb2^vii^—Yb1—Si1	143.83 (13)	Yb2^iv^—Yb2—Yb2^viii^	61.76 (6)
Yb2^vii^—Yb1—Si1^ix^	111.0 (2)	Yb2^iv^—Yb2—Yb2^xiv^	61.76 (6)
Yb2^vii^—Yb1—Si1^x^	59.15 (14)	Yb2^iv^—Yb2—Si1	50.27 (16)
Yb2^vii^—Yb1—O2^vi^	62.9 (4)	Yb2^iv^—Yb2—Si1^xi^	115.4 (2)
Yb2^vii^—Yb1—O2^vii^	108.1 (3)	Yb2^iv^—Yb2—O3^xiii^	94.0 (3)
Yb2^vii^—Yb1—O2^viii^	41.3 (4)	Yb2^iv^—Yb2—O3^xv^	94.0 (3)
Yb2^viii^—Yb1—Si1	59.15 (8)	Yb2^iv^—Yb2—O4	30.00 (5)
Yb2^viii^—Yb1—Si1^ix^	143.8 (2)	Yb2^xi^—Yb2—Yb2^xii^	61.76 (4)
Yb2^viii^—Yb1—Si1^x^	111.00 (13)	Yb2^xi^—Yb2—Yb2^xiii^	61.76 (4)
Yb2^viii^—Yb1—O2^vi^	41.3 (4)	Yb2^xi^—Yb2—Yb2^viii^	90.00 (6)
Yb2^viii^—Yb1—O2^vii^	62.9 (4)	Yb2^xi^—Yb2—Yb2^xiv^	90.00 (6)
Yb2^viii^—Yb1—O2^viii^	108.1 (3)	Yb2^xi^—Yb2—Si1	110.27 (17)
Si1—Yb1—Si1^ix^	95.2 (2)	Yb2^xi^—Yb2—Si1^xi^	55.4 (2)
Si1—Yb1—Si1^x^	95.2 (2)	Yb2^xi^—Yb2—O3^xiii^	111.47 (15)
Si1—Yb1—O2^vi^	96.5 (3)	Yb2^xi^—Yb2—O3^xv^	111.47 (15)
Si1—Yb1—O2^vii^	92.6 (4)	Yb2^xi^—Yb2—O4	30.00 (4)
Si1—Yb1—O2^viii^	165.3 (4)	Yb2^xii^—Yb2—Yb2^xiii^	113.77 (5)
Si1^ix^—Yb1—Si1^x^	95.2 (2)	Yb2^xii^—Yb2—Yb2^viii^	56.48 (4)
Si1^ix^—Yb1—O2^vi^	165.3 (3)	Yb2^xii^—Yb2—Yb2^xiv^	148.29 (9)
Si1^ix^—Yb1—O2^vii^	96.5 (3)	Yb2^xii^—Yb2—Si1	114.84 (9)
Si1^ix^—Yb1—O2^viii^	92.6 (3)	Yb2^xii^—Yb2—Si1^xi^	60.44 (8)
Si1^x^—Yb1—O2^vi^	92.6 (3)	Yb2^xii^—Yb2—O3^xiii^	168.7 (2)
Si1^x^—Yb1—O2^vii^	165.3 (3)	Yb2^xii^—Yb2—O3^xv^	55.76 (15)
Si1^x^—Yb1—O2^viii^	96.5 (4)	Yb2^xii^—Yb2—O4	74.15 (5)
O2^vi^—Yb1—O2^vii^	74.1 (4)	Yb2^xiii^—Yb2—Yb2^viii^	148.29 (9)
O2^vi^—Yb1—O2^viii^	74.1 (4)	Yb2^xiii^—Yb2—Yb2^xiv^	56.48 (4)
O2^vii^—Yb1—O2^viii^	74.1 (6)	Yb2^xiii^—Yb2—Si1	114.84 (9)
Ca1^xviii^—Ca2—Ca1^xix^	104.16 (6)	Yb2^xiii^—Yb2—Si1^xi^	60.44 (8)
Ca1^xviii^—Ca2—Ca1^xx^	84.03 (5)	Yb2^xiii^—Yb2—O3^xiii^	55.76 (15)
Ca1^xviii^—Ca2—Ca1^xxi^	49.54 (10)	Yb2^xiii^—Yb2—O3^xv^	168.7 (2)
Ca1^xviii^—Ca2—Yb1^xviii^	0	Yb2^xiii^—Yb2—O4	74.15 (5)
Ca1^xviii^—Ca2—Yb1^xix^	104.16 (6)	Yb2^viii^—Yb2—Yb2^xiv^	113.77 (8)
Ca1^xviii^—Ca2—Yb1^xx^	84.03 (5)	Yb2^viii^—Yb2—Si1	59.17 (5)
Ca1^xviii^—Ca2—Yb1^xxi^	49.54 (10)	Yb2^viii^—Yb2—Si1^xi^	116.74 (9)
Ca1^xviii^—Ca2—Ca2^iv^	150.19 (6)	Yb2^viii^—Yb2—O3^xiii^	134.4 (2)
Ca1^xviii^—Ca2—Ca2^xi^	101.65 (6)	Yb2^viii^—Yb2—O3^xv^	32.5 (3)
Ca1^xviii^—Ca2—Ca2^xii^	60.19 (8)	Yb2^viii^—Yb2—O4	74.15 (7)
Ca1^xviii^—Ca2—Ca2^xiii^	101.81 (10)	Yb2^xiv^—Yb2—Si1	59.17 (5)
Ca1^xviii^—Ca2—Ca2^viii^	97.63 (8)	Yb2^xiv^—Yb2—Si1^xi^	116.74 (9)
Ca1^xviii^—Ca2—Ca2^xiv^	146.57 (9)	Yb2^xiv^—Yb2—O3^xiii^	32.5 (3)
Ca1^xviii^—Ca2—Yb2	0	Yb2^xiv^—Yb2—O3^xv^	134.4 (2)
Ca1^xviii^—Ca2—Yb2^iv^	150.19 (6)	Yb2^xiv^—Yb2—O4	74.15 (7)
Ca1^xviii^—Ca2—Yb2^xi^	101.65 (6)	Si1—Yb2—Si1^xi^	165.7 (3)
Ca1^xviii^—Ca2—Yb2^xii^	60.19 (8)	Si1—Yb2—O3^xiii^	75.5 (2)
Ca1^xviii^—Ca2—Yb2^xiii^	101.81 (10)	Si1—Yb2—O3^xv^	75.5 (2)
Ca1^xviii^—Ca2—Yb2^viii^	97.63 (8)	Si1—Yb2—O4	80.27 (18)
Ca1^xviii^—Ca2—Yb2^xiv^	146.57 (9)	Si1^xi^—Yb2—O3^xiii^	108.4 (2)
Ca1^xviii^—Ca2—Si1	139.51 (13)	Si1^xi^—Yb2—O3^xv^	108.4 (2)
Ca1^xviii^—Ca2—Si1^xi^	52.1 (2)	Si1^xi^—Yb2—O4	85.4 (2)
Ca1^xviii^—Ca2—O1^xi^	32.19 (17)	O3^xiii^—Yb2—O3^xv^	134.2 (2)
Ca1^xviii^—Ca2—O3^xi^	43.3 (3)	O3^xiii^—Yb2—O4	104.6 (2)
Ca1^xviii^—Ca2—O3^xiii^	115.3 (3)	O3^xv^—Yb2—O4	104.6 (2)
Ca1^xviii^—Ca2—O3^xv^	69.9 (3)	Ca1—Si1—Ca1^ii^	63.1 (2)
Ca1^xviii^—Ca2—O3^xvi^	72.6 (2)	Ca1—Si1—Yb1	0
Ca1^xviii^—Ca2—O4	128.14 (6)	Ca1—Si1—Yb1^ii^	63.1 (2)
Ca1^xix^—Ca2—Ca1^xx^	49.19 (10)	Ca1—Si1—Ca2	139.10 (16)
Ca1^xix^—Ca2—Ca1^xxi^	84.03 (5)	Ca1—Si1—Ca2^iv^	79.9 (2)
Ca1^xix^—Ca2—Yb1^xviii^	104.16 (6)	Ca1—Si1—Yb2	139.10 (16)
Ca1^xix^—Ca2—Yb1^xix^	0	Ca1—Si1—Yb2^iv^	79.9 (2)
Ca1^xix^—Ca2—Yb1^xx^	49.19 (10)	Ca1—Si1—O1^ix^	44.4 (4)
Ca1^xix^—Ca2—Yb1^xxi^	84.03 (5)	Ca1—Si1—O2^xvii^	137.7 (3)
Ca1^xix^—Ca2—Ca2^iv^	101.13 (6)	Ca1—Si1—O3	60.5 (4)
Ca1^xix^—Ca2—Ca2^xi^	150.00 (8)	Ca1—Si1—O3^ii^	109.4 (6)
Ca1^xix^—Ca2—Ca2^xii^	146.51 (8)	Ca1^ii^—Si1—Yb1	63.1 (2)
Ca1^xix^—Ca2—Ca2^xiii^	97.87 (7)	Ca1^ii^—Si1—Yb1^ii^	0
Ca1^xix^—Ca2—Ca2^viii^	101.37 (8)	Ca1^ii^—Si1—Ca2	139.10 (16)
Ca1^xix^—Ca2—Ca2^xiv^	60.00 (7)	Ca1^ii^—Si1—Ca2^iv^	79.9 (2)
Ca1^xix^—Ca2—Yb2	0	Ca1^ii^—Si1—Yb2	139.10 (16)
Ca1^xix^—Ca2—Yb2^iv^	101.13 (6)	Ca1^ii^—Si1—Yb2^iv^	79.9 (2)
Ca1^xix^—Ca2—Yb2^xi^	150.00 (8)	Ca1^ii^—Si1—O1^ix^	44.4 (4)
Ca1^xix^—Ca2—Yb2^xii^	146.51 (8)	Ca1^ii^—Si1—O2^xvii^	137.7 (3)
Ca1^xix^—Ca2—Yb2^xiii^	97.87 (7)	Ca1^ii^—Si1—O3	109.4 (6)
Ca1^xix^—Ca2—Yb2^viii^	101.37 (8)	Ca1^ii^—Si1—O3^ii^	60.5 (4)
Ca1^xix^—Ca2—Yb2^xiv^	60.00 (7)	Yb1—Si1—Yb1^ii^	63.1 (2)
Ca1^xix^—Ca2—Si1	55.95 (14)	Yb1—Si1—Ca2	139.10 (16)
Ca1^xix^—Ca2—Si1^xi^	136.0 (2)	Yb1—Si1—Ca2^iv^	79.9 (2)
Ca1^xix^—Ca2—O1^xi^	75.2 (2)	Yb1—Si1—Yb2	139.10 (16)
Ca1^xix^—Ca2—O3^xi^	147.4 (3)	Yb1—Si1—Yb2^iv^	79.9 (2)
Ca1^xix^—Ca2—O3^xiii^	42.54 (16)	Yb1—Si1—O1^ix^	44.4 (4)
Ca1^xix^—Ca2—O3^xv^	91.72 (16)	Yb1—Si1—O2^xvii^	137.7 (3)
Ca1^xix^—Ca2—O3^xvi^	114.5 (2)	Yb1—Si1—O3	60.5 (4)
Ca1^xix^—Ca2—O4	127.70 (9)	Yb1—Si1—O3^ii^	109.4 (6)
Ca1^xx^—Ca2—Ca1^xxi^	104.16 (6)	Yb1^ii^—Si1—Ca2	139.10 (16)
Ca1^xx^—Ca2—Yb1^xviii^	84.03 (5)	Yb1^ii^—Si1—Ca2^iv^	79.9 (2)
Ca1^xx^—Ca2—Yb1^xix^	49.19 (10)	Yb1^ii^—Si1—Yb2	139.10 (16)
Ca1^xx^—Ca2—Yb1^xx^	0	Yb1^ii^—Si1—Yb2^iv^	79.9 (2)
Ca1^xx^—Ca2—Yb1^xxi^	104.16 (6)	Yb1^ii^—Si1—O1^ix^	44.4 (4)
Ca1^xx^—Ca2—Ca2^iv^	101.13 (6)	Yb1^ii^—Si1—O2^xvii^	137.7 (3)
Ca1^xx^—Ca2—Ca2^xi^	150.00 (8)	Yb1^ii^—Si1—O3	109.4 (6)
Ca1^xx^—Ca2—Ca2^xii^	97.87 (7)	Yb1^ii^—Si1—O3^ii^	60.5 (4)
Ca1^xx^—Ca2—Ca2^xiii^	146.51 (8)	Ca2—Si1—Ca2^iv^	74.29 (15)
Ca1^xx^—Ca2—Ca2^viii^	60.00 (7)	Ca2—Si1—Yb2	0
Ca1^xx^—Ca2—Ca2^xiv^	101.37 (8)	Ca2—Si1—Yb2^iv^	74.29 (15)
Ca1^xx^—Ca2—Yb2	0	Ca2—Si1—O1^ix^	174.5 (7)
Ca1^xx^—Ca2—Yb2^iv^	101.13 (6)	Ca2—Si1—O2^xvii^	57.3 (5)
Ca1^xx^—Ca2—Yb2^xi^	150.00 (8)	Ca2—Si1—O3	78.6 (4)
Ca1^xx^—Ca2—Yb2^xii^	97.87 (7)	Ca2—Si1—O3^ii^	78.6 (4)
Ca1^xx^—Ca2—Yb2^xiii^	146.51 (8)	Ca2^iv^—Si1—Yb2	74.29 (15)
Ca1^xx^—Ca2—Yb2^viii^	60.00 (7)	Ca2^iv^—Si1—Yb2^iv^	0
Ca1^xx^—Ca2—Yb2^xiv^	101.37 (8)	Ca2^iv^—Si1—O1^ix^	111.2 (7)
Ca1^xx^—Ca2—Si1	55.95 (14)	Ca2^iv^—Si1—O2^xvii^	131.6 (6)
Ca1^xx^—Ca2—Si1^xi^	136.0 (2)	Ca2^iv^—Si1—O3	52.1 (3)
Ca1^xx^—Ca2—O1^xi^	75.2 (2)	Ca2^iv^—Si1—O3^ii^	52.1 (3)
Ca1^xx^—Ca2—O3^xi^	114.5 (2)	Yb2—Si1—Yb2^iv^	74.29 (15)
Ca1^xx^—Ca2—O3^xiii^	91.72 (16)	Yb2—Si1—O1^ix^	174.5 (7)
Ca1^xx^—Ca2—O3^xv^	42.54 (16)	Yb2—Si1—O2^xvii^	57.3 (5)
Ca1^xx^—Ca2—O3^xvi^	147.4 (3)	Yb2—Si1—O3	78.6 (4)
Ca1^xx^—Ca2—O4	127.70 (9)	Yb2—Si1—O3^ii^	78.6 (4)
Ca1^xxi^—Ca2—Yb1^xviii^	49.54 (10)	Yb2^iv^—Si1—O1^ix^	111.2 (7)
Ca1^xxi^—Ca2—Yb1^xix^	84.03 (5)	Yb2^iv^—Si1—O2^xvii^	131.6 (6)
Ca1^xxi^—Ca2—Yb1^xx^	104.16 (6)	Yb2^iv^—Si1—O3	52.1 (3)
Ca1^xxi^—Ca2—Yb1^xxi^	0	Yb2^iv^—Si1—O3^ii^	52.1 (3)
Ca1^xxi^—Ca2—Ca2^iv^	150.19 (6)	O1^ix^—Si1—O2^xvii^	117.2 (8)
Ca1^xxi^—Ca2—Ca2^xi^	101.65 (6)	O1^ix^—Si1—O3	104.6 (6)
Ca1^xxi^—Ca2—Ca2^xii^	101.81 (10)	O1^ix^—Si1—O3^ii^	104.6 (6)
Ca1^xxi^—Ca2—Ca2^xiii^	60.19 (8)	O2^xvii^—Si1—O3	112.6 (6)
Ca1^xxi^—Ca2—Ca2^viii^	146.57 (9)	O2^xvii^—Si1—O3^ii^	112.6 (6)
Ca1^xxi^—Ca2—Ca2^xiv^	97.63 (8)	O3—Si1—O3^ii^	104.1 (5)
Ca1^xxi^—Ca2—Yb2	0	Ca1—O1—Ca1^ii^	91.6 (4)
Ca1^xxi^—Ca2—Yb2^iv^	150.19 (6)	Ca1—O1—Ca2^iv^	114.3 (5)
Ca1^xxi^—Ca2—Yb2^xi^	101.65 (6)	Ca1—O1—Si1^x^	106.4 (5)
Ca1^xxi^—Ca2—Yb2^xii^	101.81 (10)	Ca1^ii^—O1—Ca2^iv^	114.3 (5)
Ca1^xxi^—Ca2—Yb2^xiii^	60.19 (8)	Ca1^ii^—O1—Si1^x^	106.4 (5)
Ca1^xxi^—Ca2—Yb2^viii^	146.57 (9)	Ca2^iv^—O1—Si1^x^	119.9 (6)
Ca1^xxi^—Ca2—Yb2^xiv^	97.63 (8)	Ca1^xix^—O2—Ca1^xx^	91.8 (3)
Ca1^xxi^—Ca2—Si1	139.51 (13)	Ca1^xix^—O2—Yb1^xix^	0
Ca1^xxi^—Ca2—Si1^xi^	52.1 (2)	Ca1^xix^—O2—Yb1^xx^	91.8 (3)
Ca1^xxi^—Ca2—O1^xi^	32.19 (17)	Ca1^xix^—O2—Si1^v^	128.6 (4)
Ca1^xxi^—Ca2—O3^xi^	72.6 (2)	Ca1^xx^—O2—Yb1^xix^	91.8 (3)
Ca1^xxi^—Ca2—O3^xiii^	69.9 (3)	Ca1^xx^—O2—Yb1^xx^	0
Ca1^xxi^—Ca2—O3^xv^	115.3 (3)	Ca1^xx^—O2—Si1^v^	128.6 (4)
Ca1^xxi^—Ca2—O3^xvi^	43.3 (3)	Yb1^xix^—O2—Yb1^xx^	91.8 (3)
Ca1^xxi^—Ca2—O4	128.14 (6)	Yb1^xix^—O2—Si1^v^	128.6 (4)
Yb1^xviii^—Ca2—Yb1^xix^	104.16 (6)	Yb1^xx^—O2—Si1^v^	128.6 (4)
Yb1^xviii^—Ca2—Yb1^xx^	84.03 (5)	Ca2^iv^—O3—Ca2^viii^	116.6 (2)
Yb1^xviii^—Ca2—Yb1^xxi^	49.54 (10)	Ca2^iv^—O3—Yb2^viii^	116.6 (2)
Yb1^xviii^—Ca2—Ca2^iv^	150.19 (6)	Ca2^iv^—O3—Si1	97.0 (4)
Yb1^xviii^—Ca2—Ca2^xi^	101.65 (6)	Ca2^viii^—O3—Yb2^viii^	0
Yb1^xviii^—Ca2—Ca2^xii^	60.19 (8)	Ca2^viii^—O3—Si1	139.7 (6)
Yb1^xviii^—Ca2—Ca2^xiii^	101.81 (10)	Yb2^viii^—O3—Si1	139.7 (6)
Yb1^xviii^—Ca2—Ca2^viii^	97.63 (8)	Ca2—O4—Ca2^iv^	120.00 (9)
Yb1^xviii^—Ca2—Ca2^xiv^	146.57 (9)	Ca2—O4—Ca2^xi^	120.00 (10)
Yb1^xviii^—Ca2—Yb2	0	Ca2—O4—Yb2	0
Yb1^xviii^—Ca2—Yb2^iv^	150.19 (6)	Ca2—O4—Yb2^iv^	120.00 (9)
Yb1^xviii^—Ca2—Yb2^xi^	101.65 (6)	Ca2—O4—Yb2^xi^	120.00 (10)
Yb1^xviii^—Ca2—Yb2^xii^	60.19 (8)	Ca2^iv^—O4—Ca2^xi^	120.00 (10)
Yb1^xviii^—Ca2—Yb2^xiii^	101.81 (10)	Ca2^iv^—O4—Yb2	120.00 (9)
Yb1^xviii^—Ca2—Yb2^viii^	97.63 (8)	Ca2^iv^—O4—Yb2^iv^	0
Yb1^xviii^—Ca2—Yb2^xiv^	146.57 (9)	Ca2^iv^—O4—Yb2^xi^	120.00 (10)
Yb1^xviii^—Ca2—Si1	139.51 (13)	Ca2^xi^—O4—Yb2	120.00 (10)
Yb1^xviii^—Ca2—Si1^xi^	52.1 (2)	Ca2^xi^—O4—Yb2^iv^	120.00 (10)
Yb1^xviii^—Ca2—O1^xi^	32.19 (17)	Ca2^xi^—O4—Yb2^xi^	0
Yb1^xviii^—Ca2—O3^xi^	43.3 (3)	Yb2—O4—Yb2^iv^	120.00 (9)
Yb1^xviii^—Ca2—O3^xiii^	115.3 (3)	Yb2—O4—Yb2^xi^	120.00 (10)
Yb1^xviii^—Ca2—O3^xv^	69.9 (3)	Yb2^iv^—O4—Yb2^xi^	120.00 (10)

Symmetry codes: (i) *x*, *y*, −*z*−1/2; (ii) *x*, *y*, −*z*+1/2; (iii) *x*, *y*+1, *z*; (iv) −*y*, *x*−*y*, *z*; (v) −*x*+*y*+1, −*x*+1, *z*; (vi) −*x*+1, −*y*+1, *z*−1/2; (vii) *y*, −*x*+*y*+1, *z*−1/2; (viii) *x*−*y*, *x*, *z*−1/2; (ix) −*y*+1, *x*−*y*+1, *z*; (x) −*x*+*y*, −*x*+1, *z*; (xi) −*x*+*y*, −*x*, *z*; (xii) *y*, −*x*+*y*, *z*−1/2; (xiii) *y*, −*x*+*y*, *z*+1/2; (xiv) *x*−*y*, *x*, *z*+1/2; (xv) *y*, −*x*+*y*, −*z*; (xvi) −*x*+*y*, −*x*, −*z*+1/2; (xvii) −*y*+1, *x*−*y*, *z*; (xviii) *x*, *y*−1, *z*; (xix) −*x*+1, −*y*+1, *z*+1/2; (xx) −*x*+1, −*y*+1, −*z*; (xxi) *x*, *y*−1, −*z*+1/2.

### Sodium lanthanum silicate oxyapatite (Na-La) Crystal data

**Table:**

NaLa_9_(SiO_4_)_6_O_2_	*Z* = 1
*M**_r_* = 1857.63	*D*_x_ = 5.279 Mg m^−^^3^
Hexagonal, *P*6_3_/*m*	Cu *K*α radiation, λ = 1.54188 Å
*a* = 9.69061 (7) Å	*T* = 295 K
*c* = 7.18567 (6) Å	white
*V* = 584.39 (1) Å^3^	flat_sheet, 25 × 25 mm

### Sodium lanthanum silicate oxyapatite (Na-La) Data collection

**Table:**

Bruker D8 Advance diffractometer	Data collection mode: reflection
Radiation source: sealed X-ray tube	Scan method: step
Specimen mounting: packed powder pellet	2θ_min_ = 10°, 2θ_max_ = 70°, 2θ_step_ = 0.009°

### Sodium lanthanum silicate oxyapatite (Na-La) Refinement

**Table:**

*R*_p_ = 0.04	29 parameters
*R*_wp_ = 0.06	Weighting scheme based on measured s.u.'s
*R*_exp_ = 0.03	(Δ/σ)_max_ = 0.037
*R*_Bragg_ = 0.05	Background function: Chebychev
6994 data points	Preferred orientation correction: spherical harmonic
Profile function: pseudo-Voigt	

### Sodium lanthanum silicate oxyapatite (Na-La) Special details

**Table:**

Refinement. Beq were fixed as 1Å squared during refinement as they result high errors

### Sodium lanthanum silicate oxyapatite (Na-La) Fractional atomic coordinates and isotropic or equivalent isotropic displacement parameters (Å^2^)

**Table:**

	*x*	*y*	*z*	*U*_iso_*/*U*_eq_	Occ. (<1)
Na1	0.333333	0.666667	−0.002779	0.0127*	0.25
La1	0.333333	0.666667	−0.002779	0.0127*	0.75
La2	0.231568	−0.013605	0.25	0.0127*	
Si1	0.402659	0.370809	0.25	0.0127*	
O1	0.328041	0.481344	0.25	0.0127*	
O2	0.588006	0.463109	0.25	0.0127*	
O3	0.336277	0.252542	0.074479	0.0127*	
O4	0	0	0.25	0.0127*	

### Sodium lanthanum silicate oxyapatite (Na-La) Atomic displacement parameters (Å^2^)

**Table:**

	*U*^11^	*U*^22^	*U*^33^	*U*^12^	*U*^13^	*U*^23^
?	?	?	?	?	?	?

### Sodium lanthanum silicate oxyapatite (Na-La) Geometric parameters (Å, º)

**Table:**

Na1—Na1^i^	3.5529	La1—O2^v^	2.5054
Na1—Na1^ii^	3.6328	La1—O2^vi^	2.5054
Na1—La1	0	La1—O2^vii^	2.5054
Na1—La1^i^	3.5529	La2—O3^viii^	2.4653
Na1—La1^ii^	3.6328	La2—O3^ix^	2.4653
La1—La1^i^	3.5529	La2—O4	2.3128
La1—La1^ii^	3.6328	Si1—O1	1.5636
La1—O1	2.5367	Si1—O2	1.5555
La1—O1^iii^	2.5367	Si1—O3	1.6065
La1—O1^iv^	2.5367	Si1—O3^ii^	1.6065
			
Na1^i^—Na1—Na1^ii^	180	La1^i^—La1—O2^vi^	44.84
Na1^i^—Na1—La1	0	La1^i^—La1—O2^vii^	44.84
Na1^i^—Na1—La1^i^	0	La1^ii^—La1—O1	44.27
Na1^i^—Na1—La1^ii^	180	La1^ii^—La1—O1^iii^	44.27
Na1^ii^—Na1—La1	0	La1^ii^—La1—O1^iv^	44.27
Na1^ii^—Na1—La1^i^	180	La1^ii^—La1—O2^v^	135.16
Na1^ii^—Na1—La1^ii^	0	La1^ii^—La1—O2^vi^	135.16
La1—Na1—La1^i^	0	La1^ii^—La1—O2^vii^	135.16
La1—Na1—La1^ii^	0	O1—La1—O1^iii^	74.39
La1^i^—Na1—La1^ii^	180	O1—La1—O1^iv^	74.39
Na1—La1—Na1^i^	0	O1—La1—O2^v^	93.2
Na1—La1—Na1^ii^	0	O1—La1—O2^vi^	154.53
Na1—La1—La1^i^	0	O1—La1—O2^vii^	124.37
Na1—La1—La1^ii^	0	O1^iii^—La1—O1^iv^	74.39
Na1—La1—O1	0	O1^iii^—La1—O2^v^	124.36
Na1—La1—O1^iii^	0	O1^iii^—La1—O2^vi^	93.2
Na1—La1—O1^iv^	0	O1^iii^—La1—O2^vii^	154.53
Na1—La1—O2^v^	0	O1^iv^—La1—O2^v^	154.53
Na1—La1—O2^vi^	0	O1^iv^—La1—O2^vi^	124.37
Na1—La1—O2^vii^	0	O1^iv^—La1—O2^vii^	93.2
Na1^i^—La1—Na1^ii^	180	O2^v^—La1—O2^vi^	75.28
Na1^i^—La1—La1^i^	0	O2^v^—La1—O2^vii^	75.28
Na1^i^—La1—La1^ii^	180	O2^vi^—La1—O2^vii^	75.28
Na1^i^—La1—O1	135.73	O3^viii^—La2—O3^ix^	142.09
Na1^i^—La1—O1^iii^	135.73	O3^viii^—La2—O4	103.4
Na1^i^—La1—O1^iv^	135.73	O3^ix^—La2—O4	103.4
Na1^i^—La1—O2^v^	44.84	O1—Si1—O2	113.74
Na1^i^—La1—O2^vi^	44.84	O1—Si1—O3	109.35
Na1^i^—La1—O2^vii^	44.84	O1—Si1—O3^ii^	109.35
Na1^ii^—La1—La1^i^	180	O2—Si1—O3	110.22
Na1^ii^—La1—La1^ii^	0	O2—Si1—O3^ii^	110.22
Na1^ii^—La1—O1	44.27	O3—Si1—O3^ii^	103.46
Na1^ii^—La1—O1^iii^	44.27	La1—O1—La1^ii^	91.46
Na1^ii^—La1—O1^iv^	44.27	La1—O1—Si1	129.23
Na1^ii^—La1—O2^v^	135.16	La1^ii^—O1—Si1	129.23
Na1^ii^—La1—O2^vi^	135.16	La1^x^—O2—La1^xi^	90.32
Na1^ii^—La1—O2^vii^	135.16	La1^x^—O2—Si1	105.37
La1^i^—La1—La1^ii^	180	La1^xi^—O2—Si1	105.37
La1^i^—La1—O1	135.73	La2^vii^—O3—Si1	143.18
La1^i^—La1—O1^iii^	135.73	La2—O4—La2^xii^	120
La1^i^—La1—O1^iv^	135.73	La2—O4—La2^xiii^	120
La1^i^—La1—O2^v^	44.84	La2^xii^—O4—La2^xiii^	120

Symmetry codes: (i) *x*, *y*, −*z*−1/2; (ii) *x*, *y*, −*z*+1/2; (iii) −*y*+1, *x*−*y*+1, *z*; (iv) −*x*+*y*, −*x*+1, *z*; (v) −*x*+1, −*y*+1, *z*−1/2; (vi) *y*, −*x*+*y*+1, *z*−1/2; (vii) *x*−*y*, *x*, *z*−1/2; (viii) *y*, −*x*+*y*, *z*+1/2; (ix) *y*, −*x*+*y*, −*z*; (x) −*x*+1, −*y*+1, *z*+1/2; (xi) −*x*+1, −*y*+1, −*z*; (xii) −*y*, *x*−*y*, *z*; (xiii) −*x*+*y*, −*x*, *z*.
